# ISG15^+^ Müller glial subtype contributes to early blood–retinal barrier breakdown in diabetic retinopathy

**DOI:** 10.1172/JCI205800

**Published:** 2026-10-15

**Authors:** Tian Niu, Yan Jiang, Xindan Xing, Qian Zhu, Xinyi Liu, Kangjia Lv, Junwei Fang, Kun Liu

**Affiliations:** Department of Ophthalmology, National Clinical Research Center for Eye Diseases, Shanghai Key Laboratory of Ocular Fundus Diseases, Shanghai Engineering Center for Visual Science and Photomedicine, Shanghai Engineering Center for Precise Diagnosis and Treatment of Eye Diseases, Shanghai General Hospital, Shanghai Jiao Tong University School of Medicine, Shanghai, China.

**Keywords:** Cell biology, Ophthalmology, Vascular biology, Cell stress, Innate immunity, Retinopathy

## Abstract

Diabetic retinopathy features early glial stress and neurovascular dysfunction, yet Müller glial programs linking inflammatory cues to oxidative injury remain unclear. We identify a spatially clustered interferon-stimulated gene 15 (ISG15)^+^ Müller glial subtype expanded in streptozotocin-induced diabetic mice. High glucose alone did not increase basal free ISG15 or detectable ISGylation (ISG15-dependent protein modification) in primary Müller glia, whereas conditioned medium from high glucose–exposed microglia induced both, recapitulated by IFN-β and attenuated by type I interferon receptor 1 inhibition. In vivo, *Ifnb1* RNAscope signals were associated with microglia, retinal *Ifnb1* expression increased in diabetic retinas, and microglial depletion reduced *Ifnb1* expression and limited the expansion of ISG15^+^ Müller glia. Müller glia–specific *Isg15* deletion preserved blood–retinal barrier integrity, reduced acellular capillaries and Evans blue leakage, and mitigated oxidative stress, lipid peroxidation, and glutathione redox imbalance. Loss of ISG15 or disruption of ISGylation blunted interferon-driven oxidative stress in Müller glia and reduced Müller glia–conditioned medium–dependent endothelial migration and tube formation. Mechanistically, IFN-β promoted peroxiredoxin 6 (PRDX6) ISGylation and proteasome-dependent degradation without altering *Prdx6* transcripts. Thus, microglial IFN-β promotes an ISG15^+^ Müller glial subtype that compromises antioxidant defense and promotes early barrier injury in diabetic retinopathy.

## Abstract 2

We discovered a diabetes-induced ISG15-positive Müller glial subtype, triggered by microglial immune signaling, that weakens antioxidant defenses and contributes to early retinal vascular damage.

## Graphical abstract

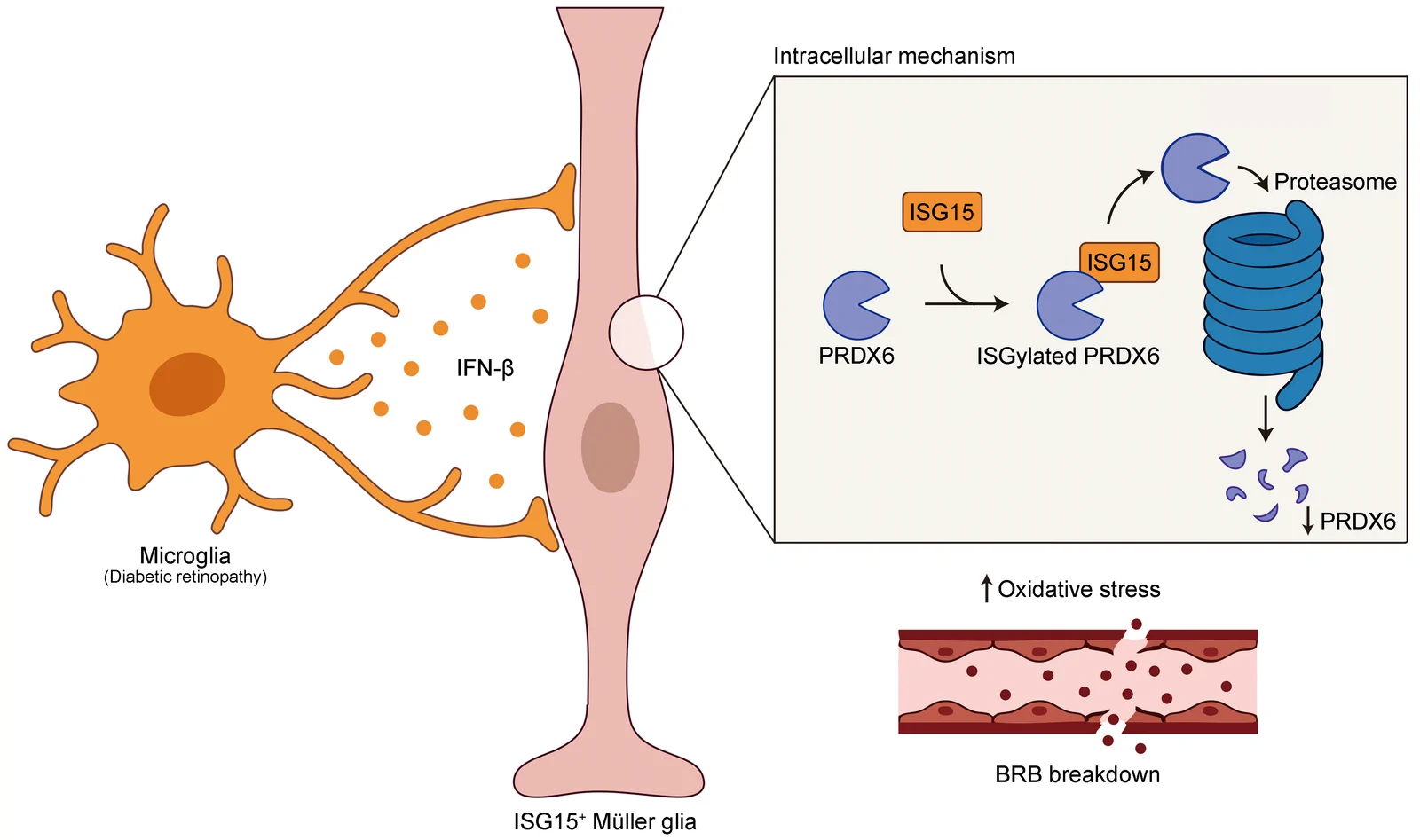

## Introduction

Diabetic retinopathy (DR) remains a leading cause of vision loss worldwide, and its prevalence continues to rise alongside the global increase in diabetes (1). Although traditionally viewed as a microvascular disease, mounting clinical and experimental evidence indicates that neuronal and glial abnormalities emerge early in the diabetic retina and may precede detectable vascular lesions (1), highlighting a critical window in which early cellular dysfunction may shape subsequent disease progression. Understanding the cellular processes that underlie these early changes is essential for improving both diagnosis and intervention.

Among retinal glia, Müller glia play indispensable roles in metabolic support, antioxidant defense, and neurovascular coupling (2). However, how Müller glia respond to chronic diabetic stress remains incompletely defined. In particular, it remains unknown whether diabetes actively drives the emergence of distinct Müller glial subtypes with defined molecular identities and functional consequences, rather than eliciting a uniform reactive response. This gap has limited the understanding of early pathogenic events in DR and constrained efforts to identify cell-type–specific therapeutic targets.

Innate immune pathways have recently emerged as potential contributors to diabetic retinal injury. Type I IFNs (IFN-I), best known for antiviral defense, are increasingly implicated in sterile inflammatory and neurodegenerative disorders of the CNS (3–5). IFN-β, in particular, has been associated with oxidative stress, microglial activation, and tissue fragility. Notably, systemic IFN-I therapy has been associated with retinal vascular complications, most commonly cotton wool spots and retinal hemorrhages (6, 7). Elevated IFN-β has also been detected in the vitreous of patients with proliferative DR (8), suggesting that dysregulated interferon activity may contribute directly to retinal pathology. Despite these observations, the downstream cellular targets of IFN-I within the retina — and whether Müller glia integrate interferon signals in ways relevant to DR progression — remain poorly defined.

Interferon-stimulated gene 15 (ISG15), one of the most robust interferon-stimulated proteins, functions both as a free molecule and as a ubiquitin-like modifier that alters protein stability through ISGylation (9). Although ISG15 has been implicated in inflammatory and metabolic diseases, its role in retinal glia has not been established, and whether ISG15 marks a stress-induced Müller glial subtype with pathogenic relevance to diabetes has not been explored.

In this study, we identify an ISG15^+^ Müller glial subtype that expands in diabetic retinas in response to microglia-derived IFN-β. We show that this subtype exhibits impaired antioxidant capacity and promotes retinal microvascular instability through ISGylation-dependent degradation of PRDX6, a key cytosolic antioxidant enzyme. Using complementary in vivo and in vitro approaches, we delineate a mechanistic pathway linking IFN-I signaling to Müller glial reprogramming and oxidative neurovascular injury in DR. These findings reveal an unrecognized interferon-driven Müller glial program and provide a mechanistic foundation for targeting the IFN/ISG15 axis in early DR.

## Results

### The ISG15^+^ Müller glial subtype is expanded in diabetic retinas.

Given the importance and distinctiveness of ISG15 among ISG proteins, we aimed to identify which cell types express ISG15. At 6 months after streptozotocin (STZ) induction, immunofluorescence staining showed ISG15 signal in multiple retinal compartments, including the glutamine synthetase–associated (GS-associated) Müller glial compartment, CD31^+^ vascular structures, and ionized calcium-binding adapter molecule 1 (IBA-1)^+^ microglia (Figure 1A). Importantly, ISG15^+^ Müller glia did not appear uniformly throughout the retina; instead, they formed spatially restricted clusters. This clustered organization suggests a spatially restricted Müller-associated ISG15^+^ state rather than a generalized pan-Müller reactive phenotype. To provide orthogonal transcriptomic support for this observation, we reanalyzed a published scRNA-seq dataset from control and STZ diabetic mouse retinas. At the whole-retina level, parent clusters 0 and 12 were annotated as Müller glia based on canonical marker expression, including *Glul* and *Rlbp1* (Supplemental Figure 2, A and B; supplemental material available online with this article; https://doi.org/10.1172/JCI205800DS1). Focused reclustering of these Müller glial cells resolved 6 Müller subclusters (Supplemental Figure 2C). One subcluster (cluster 5) was characterized by an interferon-stimulated gene signature, including *Isg15*, *Ifit1*, *Ifit3*, *Irf7*, *Usp18*, and *Rsad2* (Supplemental Figure 2D). We further observed higher cell-level IFN/ISG module scores in the STZ sample than in the control sample (Supplemental Figure 2E). These data support the presence of a transcriptionally distinct ISG15^+^ Müller glial subtype in diabetic retinas. In diabetic retinas, the spatially clustered pattern of ISG15^+^GS^+^ Müller-associated signals was retained, while the extent of this compartment appeared increased compared with control retinas (Figure 1B). Quantification of the ISG15^+^GS^+^ area normalized to the GS^+^ area further confirmed significant expansion of the Müller-associated ISG15^+^ compartment in STZ-diabetic retinas (Figure 1C). Together with the single-cell transcriptomic reanalysis, these findings support diabetes-associated expansion of an ISG15^+^, IFN-responsive Müller glial state.

### Microglia-derived IFN-β induces the ISG15^+^ Müller glial subtype.

High-glucose stimulation alone did not increase basal free ISG15 abundance or induce detectable ISGylated conjugates in primary Müller glia (Figure 2A), suggesting that hyperglycemia is insufficient to activate this pathway in a cell-autonomous manner. Because several studies in the brain have shown that microglia can drive astrocytes to adopt distinct reactive subtypes (10, 11), we next examined whether microglia could similarly influence ISG15 expression in Müller glia. Primary retinal microglia were cultured under normal glucose (NG) or high glucose (HG) conditions, and the resulting microglia-conditioned media (MCM) were applied to Müller glia (Figure 2B). Whereas NG-MCM produced little effect, HG-MCM robustly increased ISG15 expression and ISGylated proteins in Müller glia, indicating that microglial factors, rather than glucose itself, mediate this response. Next, we screened candidate cytokines reported to be elevated in ocular fluids of DR patients, including TNF-α, IL-1β, IFN-γ, and IFN-β (8, 12–15). Before identifying candidate mediators, we first confirmed that HG exposure upregulated these cytokines in primary retinal microglial supernatants (Figure 2C). Most cytokines yielded minimal changes, whereas IFN-β produced a strong, dose- and time-dependent induction of ISG15 in Müller glia (Figure 2, D–F). We further characterized the temporal profile of microglial IFN-β release under HG conditions. Secreted IFN-β was detectable at 24 hours, increased at 48 hours, and remained at a similar level at 72 hours (Supplemental Figure 3). We also evaluated LPS, which can induce interferon-responsive astrocytes (16); however, LPS failed to induce ISG15 or ISGylation in Müller glia (Figure 2D). This limited responsiveness likely reflects the absence of TIR domain–containing adapter-inducing interferon-β, a key adapter required for Toll-like receptor 4–mediated IFN-I signaling in Müller glia (10, 17). Interferon-α/β receptor subunit 1 (IFNAR1) blockade attenuated HG-MCM–induced ISG15 expression and ISGylation in Müller glia (Figure 2G), supporting a role for IFN-I receptor signaling in the microglia-conditioned medium response. To examine the in vivo localization of *Ifnb1* expression, we performed *Ifnb1* RNAscope combined with IBA-1 immunostaining in STZ-diabetic retinas. *Ifnb1* signals were detected in close association with microglia (Figure 3A). Retinal *Ifnb1* mRNA abundance was then quantified by RT-qPCR, which showed increased *Ifnb1* expression in STZ-diabetic retinas compared with controls (Figure 3B). To further assess the contribution of microglia to this response, we depleted microglia with PLX5622 in STZ-diabetic mice. PLX5622 treatment visibly reduced IBA-1^+^ microglia, decreased retinal *Ifnb1* mRNA expression, and attenuated expansion of the Müller-associated ISG15^+^ compartment, quantified as ISG15^+^GS^+^ area/GS^+^ area (Figure 3, C and D). These data support microglia as an important contributor to retinal IFN-β signaling and Müller-associated ISG15 induction under diabetic conditions.

### Müller glia–specific ISG15 deletion preserves retinal vascular integrity in diabetes.

At 6 months after STZ induction, to assess the role of ISG15^+^ Müller glia in diabetes-induced vasculopathy, we generated Müller glia–specific ISG15 conditional knockout mice by crossing *Isg15*^fl/fl^ mice with *Pdgfra*-Cre transgenic mice and induced diabetes using STZ. The specificity of ISG15 deletion in Müller glia was confirmed by coimmunostaining for ISG15 and the Müller glial marker GS, which demonstrated a marked loss of ISG15 signal selectively within GS^+^ Müller glia in *Isg15*^MG-KO^ retinas. At the same time, overall retinal architecture was preserved (Supplemental Figure 4). Representative baseline fundus images also showed no obvious gross abnormalities in fundus appearance or major retinal vascular pattern in *Isg15*^MG-KO^ mice compared with littermate controls (Supplemental Figure 5). Vascular leakage in DR is commonly driven by breakdown of the blood–retinal barrier (BRB), which depends on tight junction integrity within the retinal microvasculature (18). Consistent with BRB impairment, diabetic *Isg15*^fl/fl^ mice displayed reduced expression of Claudin-5 and Occludin (19), whereas Müller glia–specific ISG15 deletion preserved the expression of both proteins (Figure 4, A and B). Diabetic *Isg15*^fl/fl^ retinas also exhibited an increased number of acellular capillaries, a structural indicator of microvascular degeneration caused by endothelial and pericyte loss (20); this increase was significantly attenuated in *Isg15*^MG-KO^ mice (Figure 4, C and D). In Evans blue assays, diabetes induced robust retinal vascular leakage in *Isg15*^fl/fl^ mice, while *Isg15*^MG-KO^ mice showed markedly reduced leakage under diabetic conditions (Figure 4, E and F). Collectively, these results indicate that Müller glial ISG15 contributes to early diabetes-induced BRB dysfunction and microvascular injury.

### Müller glia–specific ISG15 deletion attenuates diabetes-induced retinal oxidative stress.

Müller glia are central to retinal antioxidant defense (21). Therefore, at 6 months after STZ induction, we tested whether expansion of ISG15^+^ Müller glia in diabetes is associated with impaired redox homeostasis. Retinal oxidative stress was visualized by dihydroethidium (DHE) staining, and antioxidant capacity was evaluated by measuring SOD and catalase (CAT) activity. Diabetic *Isg15*^fl/fl^ retinas showed increased DHE fluorescence accompanied by reduced SOD and CAT activity, whereas *Isg15*^MG-KO^ mice exhibited lower oxidative stress and preserved antioxidant enzyme activity under diabetic conditions (Figure 5, A–C).

To further characterize oxidative injury, we quantified the lipid peroxidation product malondialdehyde (MDA) and examined 4-hydroxynonenal (4-HNE)–modified proteins, which accumulate in retinal pathology and are closely linked to Müller glial oxidative vulnerability (22, 23). Diabetes increased MDA levels and enhanced 4-HNE–modified protein signals in *Isg15*^fl/fl^ retinas; these changes were attenuated in *Isg15*^MG-KO^ mice (Figure 6, A and B). In parallel, diabetes reduced retinal glutathione (GSH) abundance and the GSH/oxidized glutathione (GSSG) ratio, accompanied by altered GSSG levels, whereas *Isg15*^MG-KO^ mice showed partial restoration of these redox measures (Figure 6, C–E). These data support a role for Müller glial ISG15 in amplifying oxidative stress and lipid peroxidation in diabetic retinas. In parallel, scotopic electroretinography (ERG) performed 6 months after STZ induction showed improved retinal function in diabetic *Isg15*^MG-KO^ mice compared with diabetic *Isg15*^fl/fl^ controls, as reflected by higher a- and b-wave amplitudes, particularly at intermediate-to-high stimulus intensities (Supplemental Figure 6).

### ISG15 and ISGylation in Müller glia promote endothelial proangiogenic behaviors in vitro.

To examine whether Müller glial ISG15 influences endothelial behavior, we treated human retinal microvascular endothelial cells (HRMECs) with Müller glia–conditioned medium (MGCM) collected under the indicated conditions (Figure 7A). Primary Müller glia were isolated from *Isg15*^fl/fl^ and *Isg15*^MG-KO^ mice to assess the contribution of ISG15. To interrogate the role of ISGylation, we silenced ubiquitin-like modifier activating enzyme 7 (*Uba7*), which encodes the E1 enzyme required for initiating ISG15 conjugation, using siRNA in primary Müller glia from *Isg15*^fl/fl^ mice (24); knockdown efficiency was confirmed at both mRNA and protein levels (Supplemental Figure 7). MGCM from IFN-β–stimulated Müller glia significantly increased HRMEC tube formation (Figure 7, B and C) and migration (Figure 8, A and B) relative to controls. In contrast, MGCM from IFN-β–stimulated ISG15-deficient Müller glia showed a reduced ability to promote tube formation (Figure 7, B and C) and migration (Figure 8, A and B). A similar reduction was observed when MGCM was collected from UBA7-silenced Müller glia following IFN-β stimulation (Figure 7, B and C, and Figure 8, A and B). Together, these results indicate that ISG15 expression and ISGylation in Müller glia contribute to an IFN-β–induced oxidative stress–associated Müller glial state that promotes endothelial proangiogenic behaviors in the conditioned-medium system.

### ISG15 and ISGylation potentiate IFN-β–induced oxidative stress in Müller glia in vitro.

To validate the in vivo findings and quantify Müller glial redox responses in a controlled system, we assessed oxidative stress in primary Müller glia isolated from *Isg15*^MG-KO^ mice and in *Isg15*^fl/fl^ Müller glia following UBA7 knockdown. Intracellular ROS levels were measured using CM-H_2_DCFDA, and antioxidant capacity was evaluated by SOD and CAT activity. IFN-β stimulation increased ROS levels and reduced antioxidant enzyme activity in primary Müller glia, whereas these effects were markedly attenuated in ISG15-deficient Müller glia (Figure 9, A–D). UBA7 knockdown similarly reduced IFN-β–induced oxidative stress (Figure 9, A–D). IFN-β also increased lipid peroxidation, as measured by MDA, and this effect was reduced in ISG15-deficient Müller glia and following UBA7 knockdown (Figure 10A). Redox imbalance, assessed by GSH and GSSG levels and the GSH/GSSG ratio, showed a consistent pattern (Figure 10, B–D). These results support a model in which ISG15 and ISGylation amplify IFN-β–driven oxidative injury in Müller glia.

### ISGylation promotes PRDX6 proteasomal degradation in Müller glia.

PRDX6 has been reported as a candidate ISGylation target in proteomic datasets (25, 26). In the retina, PRDX6 is predominantly expressed in Müller glia (21, 27) and contributes to antioxidant defense and protection against lipid peroxidation (27). In vivo, diabetic retinas exhibited reduced PRDX6 protein abundance, whereas Müller glia–specific ISG15 deletion restored PRDX6 levels (Figure 11A). In vitro, IFN-β suppressed PRDX6 protein expression in primary Müller glia, and this reduction was largely rescued in ISG15-deficient Müller glia or following UBA7 knockdown (Figure 11, B and C), while *Prdx6* mRNA levels remained unchanged (Figure 11D), indicating posttranscriptional regulation.

Because PRDX6 function may depend on subcellular localization, we examined PRDX6 distribution in Müller glia (28). PRDX6 signal was predominantly cytosolic in both control and IFN-β–treated Müller glia, with no detectable enrichment in mitochondria or lysosomes (Figure 11E). This suggests that PRDX6 in Müller glia primarily exerts its antioxidant function through cytoplasmic peroxidase activity. We next tested whether ISGylation promotes PRDX6 degradation, consistent with prior reports of PRDX6 turnover via proteasomal pathways following other posttranslational modifications (29). In cycloheximide (CHX) chase assays, IFN-β accelerated PRDX6 degradation and reduced its apparent half-life (Figure 11F). MG132, but not chloroquine (CQ), restored PRDX6 levels during IFN-β treatment (Figure 11G), supporting proteasome-dependent degradation. Co-IP in primary Müller glia further showed increased PRDX6 ISGylation following IFN-β stimulation (Figure 12A). In HEK293T cells, reconstitution of the ISGylation machinery (ISG15, UBA7, UBE2L6) together with FLAG-PRDX6 reduced PRDX6 protein abundance; MG132, but not CQ, rescued PRDX6 levels (Figure 12D). Additional co-IP experiments supported association between PRDX6 and ISG15/ISGylation components (Figure 12, B and C). Collectively, these findings support PRDX6 as an ISGylation-associated substrate that is subjected to proteasome-dependent degradation in response to IFN-β, thereby reducing antioxidant capacity in Müller glia.

## Discussion

DR is increasingly recognized as a disorder that extends beyond microvascular deterioration, with early glial alterations and neuroinflammatory signaling contributing to disease progression. Although Müller glia provide essential structural, metabolic, and neurovascular support, emerging evidence indicates that their responses to diabetic stress are heterogeneous rather than uniformly reactive. In this study, we identify a distinct ISG15^+^ Müller glial subtype that expands under diabetic conditions, suggesting that a subset of Müller glia undergoes targeted transcriptional reprogramming rather than broad gliotic activation. This subtype emerges in parallel with early oxidative disequilibrium and subtle vascular instability. Functional studies further demonstrate that Müller glial ISG15 contributes to oxidative stress and microvascular abnormalities, underscoring the contribution of glial cell–intrinsic stress programs to early DR pathogenesis.

Microglia-derived IFN-β appears to contribute to the induction of this ISG15^+^ Müller glial subtype. Our in vitro findings further suggest that this pathway can be engaged early under high-glucose stress conditions. Although type I interferons are classically associated with antiviral immunity, they are now recognized as key regulators of sterile inflammatory and degenerative processes across the CNS, including aging, ischemic retinal injury, and neurodegeneration (30, 31). In traumatic brain injury, Alzheimer’s disease, and stroke, sustained IFN-I signaling enhances microglial activation, disrupts neuro-supportive pathways, and promotes long-term neurodegeneration (32–34). Across these conditions, prolonged IFN-β signaling consistently destabilizes glial homeostasis and increases tissue vulnerability.

Converging evidence indicates that microglia represent the predominant cellular source of IFN-β in neuroinflammatory contexts. In Alzheimer’s disease and autoimmune encephalomyelitis models, oligomeric Aβ or inflammatory stimulation induces robust IFN-β expression in microglia but elicits minimal responses in astrocytes or neurons (35, 36). This distinction underscores a conserved division of labor, wherein microglia serve as the principal producers of IFN-β. Parallel observations have been reported in the retina: single-cell transcriptomic profiling after optic nerve injury revealed broad IFN response signatures in Müller glia and astrocytes, with microglia acting as the primary source of *Ifnb1* (37). Together, these studies delineate a conserved communication axis in which microglial IFN-β shapes macroglial stress programs across CNS tissues.

These mechanistic themes converge with emerging insights in diabetes. The IFN-β response observed in primary retinal microglia suggests that this pathway can be engaged under diabetes-related stress conditions. In vivo, overt vasculopathy likely reflects the cumulative effects of chronic retinal stress, glial dysfunction, inflammatory and oxidative injury, and progressive impairment of the retinal neurovascular unit (1). DR is characterized by mitochondrial dysfunction and activation of the cGAS/STING/IFN-I pathway (8, 38), and aging retinas likewise exhibit heightened IFN-I activity triggered by cytosolic mitochondrial DNA accumulation, resulting in glial activation and tissue injury (31). Within this broader framework, the ISG15^+^ Müller glial subtype identified in our study aligns with a conserved interferon-driven stress response shared across retinal and CNS pathologies, establishing a biologically coherent link between microglial IFN-β activity and Müller glial reprogramming in DR. The upstream triggers that sustain retinal IFN-I activation, the temporal hierarchy between early glial stress and subsequent vascular injury, and the mechanisms linking early interferon activation to the delayed development of overt diabetic vasculopathy remain to be defined.

Mechanistically, this ISG15^+^ Müller glial subtype represents a convergence point between IFN-I signaling and disrupted redox regulation. The marked reduction in PRDX6 — a key cytosolic antioxidant enzyme — together with increased ISGylation suggest that interferon-driven posttranslational modification compromises Müller glial antioxidant defenses. Prior proteomic analyses have reported decreased PRDX6 abundance in diabetic retinas (39), consistent with its susceptibility to oxidative and inflammatory stress. ISG15 is known to modulate protein stability or activity, including ADAMTS1 and FOXO3a, often through ISGylation-dependent proteasomal pathways (40, 41). Our data extend this paradigm by identifying PRDX6 as an ISGylation substrate and linking its accelerated degradation to oxidative stress in diabetic Müller glia.

Insights from other organs further highlight ISG15 as a pathogenic amplifier of oxidative and vascular injury. In diabetic kidney disease, ISG15 enhances inflammatory pyroptosis through cGAS/STING/NLRP3 activation; in acute kidney injury, ISGylation stabilizes NOX4 and promotes ROS generation (42, 43). ISG15-mediated oxidative injury has similarly been implicated in endothelial dysfunction and vascular remodeling in hypertension (44). Collectively, these studies position ISGylation as a conserved stress-amplifying pathway across organ systems. Given the radial architecture of Müller glia and their central roles in retinal metabolic support, antioxidant homeostasis, neurotransmitter regulation, and maintenance of the inner BRB, impairment of a Müller cell–intrinsic antioxidant pathway could reasonably contribute to broader tissue-level oxidative and neurovascular disturbances beyond the Müller compartment itself (21, 45).

Our results support a working model in which persistent microglial IFN-β signaling promotes expansion of an ISG15^+^ Müller glial subtype with diminished antioxidant resilience and heightened inflammatory sensitivity. Through ISGylation-dependent degradation of PRDX6 and related functional reprogramming, this subtype may contribute to early neurovascular instability in DR. These findings support a functional contribution of Müller glial ISG15 to diabetic retinal injury, while not excluding parallel contributions from other retinal cell types and pathways. Evidence that inhibition of ISG15 or ISGylation mitigates oxidative and vascular injury in other organs provides a conceptual basis for exploring targeted modulation of the IFN/ISG15 axis in DR.

## Methods

### Sex as a biological variable.

Male mice were used because male C57BL/6J mice show more consistent susceptibility to STZ-induced diabetes under the dosing regimen used in this study, whereas female C57BL/6J mice are relatively resistant to the diabetogenic effects of STZ and may require higher STZ doses to achieve comparable hyperglycemia. Previous work has shown that, once comparable STZ-induced diabetes is established, male and female mice develop broadly similar retinal oxidative stress and capillary degeneration (46). However, because female mice were not examined in the present study, whether the specific mechanisms identified here are conserved across sexes remains to be determined.

### Animals.

WT C57BL/6J male mice were purchased from Shanghai Model Organisms Center. *Isg15*^fl/fl^ mice were established by the Shanghai Model Organisms Center on a C57BL/6J background. Mice expressing a Müller glial cell–specific Cre recombinase [C57BL/6-Tg(*Pdgfra*-cre)1Clc/J] were purchased from The Jackson Laboratory (stock 013148).

The *Isg15*^fl/fl^ mice were then crossed with *Pdgfra*-Cre mice, which have been used to direct Cre activity to retinal Müller glia. *Pdgfra*-Cre^+^ *Isg15*^fl/fl^ mice were designated as *Isg15*^MG-KO^ mice. Littermate *Isg15*^fl/fl^ mice without Cre expression were used as controls. Genotyping was performed using PCR analysis of genomic DNA extracted from mouse tail biopsies. The following primer sequences were used: *Isg15* flox: forward, 5′-TGTGGATCAGAGGAGAAAGCAG-3′, reverse, 5′-GAGTGCTTTGTCTGTCCACAG-3′; *Pdgfra*-Cre: forward, 5′-GCGGTCTGGCAGTAAAAACTATC-3′, reverse, 5′-GTGAAACAGCATTGCTGTCACTT-3′.

To induce diabetes, 8-week-old male mice received intraperitoneal STZ (Sigma-Aldrich, 50 mg/kg) for 5 consecutive days, and mice with fasting blood glucose > 250 mg/dL 7 days after the final injection were considered diabetic. For microglial depletion, mice were fed a PLX5622-containing diet (1,200 ppm; Research Diets) starting 14 days before STZ administration and were maintained on this diet throughout the experimental period; control animals received matched AIN-76A chow (Research Diets). Unless otherwise indicated, all retinal phenotypic, histological, and molecular analyses were performed 6 months after STZ induction. Baseline characterization of the genetic model, including validation immunostaining and fundus imaging, was performed in 8-week-old mice before STZ administration.

### Reanalysis of published scRNA-seq data.

A published scRNA-seq dataset from control and STZ-diabetic mouse retinas (GSE178121) was reanalyzed in Seurat (version 5.4.0). Cells were filtered using standard quality control criteria (200 < nFeature_RNA < 6,000, nCount_RNA > 500, and mitochondrial transcripts < 20%). The data were normalized using LogNormalize, followed by principal component analysis–based dimensionality reduction, clustering, and UMAP visualization. Parent retinal clusters were annotated on the basis of canonical marker genes. Clusters 0 and 12 were identified as Müller glia by expression of *Glul* and *Rlbp1*, together with lack of enrichment for markers of other major retinal populations, and were therefore selected for Müller-focused reclustering.

The Müller subset was reprocessed using the same general workflow and reclustered at resolution of 0.50 to resolve Müller glial heterogeneity. Subcluster marker genes were identified using the Seurat differential expression pipeline. Interferon-related transcriptional activity was assessed by calculating an IFN/ISG module score with Seurat:AddModuleScore using an a priori defined panel of canonical interferon-stimulated genes (*Isg15*, *Ifit1*, *Ifit2*, *Ifit3*, *Stat1*, *Stat2*, *Irf7*, *Irf9*, *Usp18*, *Mx1*, *Mx2*, *Bst2*, *Oas1a*, *Oas1g*, *Oasl2*, *Ifi27l2a*, *Ifi44*, and *Ifi47*); only genes present in the expression matrix were included in the final score calculation. IFN/ISG module scores were visualized at the cell level within Müller subcluster 5 from control and STZ-diabetic retinal samples.

### Isolation of Müller glial culture.

Primary Müller glia were isolated from P5–P7 mice, following previously described protocols with minor adjustments (47). After enucleation, retinas were carefully dissected and enzymatically dissociated using the Papain Dissociation System (Worthington) according to the manufacturer’s instructions. The resulting single-cell suspension was seeded onto culture dishes precoated with 0.1% gelatin (Sigma-Aldrich). Cells were maintained in DMEM (Gibco) containing 10% FBS (Gibco) and 1% penicillin-streptomycin (Gibco). The culture medium was refreshed every other day. After reaching confluence, cells were passaged and allowed to grow for an additional 7 days. The adherent cell population consisted predominantly of Müller glia, as verified by immunocytochemical staining for Müller glial markers and potential contaminating cell markers, including GS, CRALBP, IBA-1, GFAP, and CD31 (Supplemental Figure 1).

### Isolation of primary retinal microglia.

Primary retinal microglia were prepared from P5–P7 mice using a mixed retinal glial culture approach adapted from a published method, with minor modifications. After enucleation, retinas were dissected and dissociated using the Papain Dissociation System according to the manufacturer’s instructions. The resulting cell suspension was transferred to culture flasks containing DMEM supplemented with GlutaMAX (Gibco), 10% FBS, and 1% penicillin/streptomycin. Mixed retinal glial cultures were maintained for 2–3 weeks, with medium replacement every 2–3 days, until a confluent adherent layer had formed. Microglia were then collected from the mixed cultures by orbital shaking at 200 rpm for 6 hours, replated, and used for subsequent experiments. Primary retinal microglial cultures were assessed by immunocytochemical staining for microglial markers and potential contaminating cell markers, including IBA-1, TMEM119, GS, GFAP, and CD31 (Supplemental Figure 1).

### Cell culture and treatment conditions.

Primary Müller glia and HEK293T cells were cultured in DMEM supplemented with 10% FBS and 1% penicillin/streptomycin. HEK293T cells were obtained from the American Type Culture Collection and cultured under the same conditions. Primary retinal microglia were cultured in DMEM supplemented with GlutaMAX, 10% FBS, and 1% penicillin/streptomycin. HRMECs (Cell Systems) were maintained in endothelial cell medium (ScienCell). Before formal stimulation experiments, all cells were switched to their corresponding serum-free basal media for 24 hours and were maintained in serum-free basal media throughout the subsequent treatment period unless otherwise indicated. To mimic diabetic or inflammatory conditions, cells were exposed to NG (5.5 mM) or HG (30 mM). When indicated, cells were treated with TNF-α (PeproTech), IL-1β (PeproTech), IFN-γ (PeproTech), or IFN-β (R&D Systems) at 20 ng/mL unless otherwise indicated; LPS (Sigma-Aldrich) was used at 100 ng/mL, and PBS served as the vehicle control for IFN-β treatment. For IFN-β dose–response experiments, Müller glia were treated with the indicated concentrations of IFN-β. IFNAR signaling was inhibited by pretreating cells for 1 hour with anti-mouse IFNAR1 antibody (10 μg/mL; clone MAR1-5A3, Thermo Fisher Scientific) or an isotype control (10 μg/mL; clone P3.6.2.8.1, Thermo Fisher Scientific).

### Conditioned media preparation and perturbations.

Primary microglia were cultured under NG or HG conditions for 24 hours and then switched to NG medium for an additional 24 hours. The resulting MCM was collected and applied to Müller glia. MGCM was generated from primary Müller glia under the indicated genotype or siRNA conditions. For UBA7 knockdown, 60%–70% confluent Müller glia were transfected with UBA7 siRNA (Silencer Select, s92367, Thermo Fisher Scientific) or scrambled siRNA (4390843, Thermo Fisher Scientific) using HyperFect (Qiagen). All MGCM generation steps were performed in serum-free basal medium. At 48 hours after transfection, the medium was replaced; cells were treated the next day with IFN-β (20 ng/mL) or PBS vehicle control for 24 hours and then switched to fresh serum-free basal medium. MGCM was collected 24 hours later, clarified, mixed 1:1 with endothelial cell medium, and applied to HRMECs.

### Protein stability assays and pathway inhibition.

To assess protein stability and degradation, Müller glia were pretreated with IFN-β (20 ng/mL) for 24 hours before the addition of CHX (100 μg/mL; S7418, Selleck). For pathway inhibition, IFN-β–pretreated cells were incubated with CHX together with MG132 (25 μM; S2619, Selleck) or CQ (25 μM; S6999, Selleck) for 6 hours to block proteasomal or lysosomal degradation, respectively.

### Plasmid transfection.

For ectopic expression experiments, HEK293T cells were transfected with pcDNA3.1(+)-FLAG-PRDX6, pcDNA3.1(+)-HA-ISG15, pcDNA3.1(+)-His-UBA7, or pcDNA3.1(+)-Myc-UBE2L6 expression constructs (Obiosh Biotechnology) using Lipofectamine 3000 (Invitrogen). Corresponding empty vectors served as controls. For degradation pathway analysis, 24 hours after transfection, cells were treated with CHX alone or together with MG132 or CQ for 6 hours before immunoblot analysis.

### Oxidative stress and antioxidant enzyme assays.

Retinal tissues and primary Müller glia lysates were collected, rinsed in cold PBS, and homogenized according to the manufacturer’s instructions for each assay. Total protein concentration was determined using a bicinchoninic acid protein assay kit (Sigma-Aldrich). SOD and CAT activities were measured using commercial colorimetric assay kits (S0101S and S0051; Beyotime). Lipid peroxidation was assessed by quantifying MDA using a dedicated assay kit (ab118970, Abcam). Redox status was determined by measuring GSH and GSSG using a glutathione ratio detection kit (ab138881, Abcam). Absorbance or fluorescence values were recorded on a microplate reader and normalized to total protein content.

Intracellular ROS levels in primary Müller glia were measured using CM-H_2_DCFDA (C6827, Invitrogen). Cells were washed with PBS and incubated with 5 μM CM-H_2_DCFDA at 37°C for 30 minutes in the dark. ROS fluorescence was quantified by flow cytometry, and mean fluorescence intensity was analyzed using FlowJo.

### Immunofluorescence.

Retinal cryosections were prepared as previously described (48). Cell coverslips were prepared and fixed according to established protocols (47).

Samples were blocked in 5% donkey serum and 0.3% Triton X-100 for 1 hour at room temperature and incubated overnight at 4°C with the following primary antibodies: anti-ISG15 (ab315281, Abcam), anti-CRALBP (ab15051, Abcam), anti-GS (OB-PRT016, Oasis Biofarm), anti-GFAP (Z0334, Dako), anti-TMEM119 (PA5-119902, Thermo Fisher Scientific), anti-CD31 (AF3628, R&D Systems), anti–IBA-1 (OB-PGP049, Oasis Biofarm), anti-LAMP1 (14-1071-82, Thermo Fisher Scientific), anti-PRDX6 (MA5-49212, Thermo Fisher Scientific), and anti-TOM20 (11802-1-AP, Proteintech). After washing, samples were incubated for 1 hour at room temperature with the appropriate Alexa Fluor–conjugated secondary antibodies (Jackson ImmunoResearch). Nuclei were counterstained with DAPI for 5 minutes before mounting. Fluorescent images were acquired using a confocal microscope under identical settings across experimental groups.

Müller-associated ISG15^+^ signal was quantified using ImageJ software (version 1.53a; NIH). Briefly, the neural retina was manually outlined as the region of interest, and the GS^+^ area within this region was used to define the Müller glial compartment. The ISG15^+^ area overlapping with the GS^+^ mask was measured and expressed as ISG15^+^GS^+^ area/GS^+^ area (%). Identical imaging settings and thresholding criteria were applied across groups.

### RNAscope in situ hybridization combined with immunofluorescence.

Retinal *Ifnb1* mRNA was detected using the RNAscope Multiplex Fluorescent Reagent Kit v2 (Advanced Cell Diagnostics) with the RNAscope Probe-Mm-*Ifnb1* (406531, Advanced Cell Diagnostics) according to the manufacturer’s instructions. After signal amplification, *Ifnb1* signals were visualized using TSA Vivid Fluorophore 650 (323273, Advanced Cell Diagnostics). Sections were then processed for IBA-1 immunofluorescence, counterstained with DAPI, and imaged by confocal microscopy.

### DHE staining.

Retinal oxidative stress was assessed using DHE. Fresh cryosections were incubated with DHE (5 μM in PBS) at 37°C for 30 minutes in the dark. Sections were rinsed with PBS, mounted using an antifade medium, and imaged by confocal microscopy to detect oxidized DHE emission (~594 nm).

### Western blot.

Retinal tissues were homogenized in a protein extraction buffer containing protease and phosphatase inhibitors (Sigma-Aldrich). Protein concentrations were determined by the bicinchoninic acid assay. Equal amounts of protein were separated by SDS-PAGE and transferred to PVDF membranes. After blocking with 5% BSA for 1 hour at room temperature, membranes were incubated overnight at 4°C with primary antibodies, including anti-ISG15 (ab315281, Abcam), anti–4-HNE (ab46545, Abcam), anti-Occludin (33-1500, Thermo Fisher Scientific), anti-Claudin-5 (35-2500, Thermo Fisher Scientific), anti-PRDX6 (MA5-49212, Thermo Fisher Scientific), anti-UBA7 (sc-390097, Santa Cruz Biotechnology), anti-FLAG (F7425, Sigma-Aldrich), anti-HA (3724, Cell Signaling Technology), and anti–β-actin (4967, Cell Signaling Technology), each diluted 1:1,000. After washing, membranes were incubated with horseradish peroxidase–conjugated secondary antibodies (1:10,000; Cell Signaling Technology). Immunoreactive bands were visualized using enhanced chemiluminescence (Millipore) and quantified using ImageJ under consistent exposure conditions. For co-IP, VeriBlot IP detection reagents (Abcam) were used to minimize IgG heavy- and light-chain signals.

### Co-IP.

Protein–protein co-IP was performed using the Pierce Classic Magnetic IP/Co-IP Kit (88804, Thermo Fisher Scientific) according to the manufacturer’s instructions. Lysates were prepared from Müller glia or HEK293T cells. For IP, cell extracts were incubated with antibodies against PRDX6 (MA5-49212, Thermo Fisher Scientific), FLAG (F7425, Sigma-Aldrich), or HA (3724, Cell Signaling Technology), with normal mouse or rabbit IgG serving as negative controls. Immune complexes were captured using magnetic beads, washed, and eluted for subsequent immunoblot analysis.

### RT-qPCR analysis.

Total RNA was extracted from primary Müller glia or retinal tissues using TRIzol reagent (Invitrogen), and 1 μg RNA was reverse transcribed using the PrimeScript RT kit (Takara). RT-qPCR was performed with TB Green Premix Ex Taq (Takara) on a QuantStudio 6 Flex system (Thermo Fisher Scientific) under the following conditions: 95°C for 30 seconds, followed by 40 cycles of 95°C for 5 seconds and 60°C for 30 seconds. *Actb* served as the internal reference. Primer sequences were as follows: *Uba7* forward, 5′-CTACGAGCGACTCCATATACCT-3′, and reverse, 5′-TACACACAGGGTAGGGAGCAT-3′; *Prdx6* forward, 5′-CCTGGGAGATTCGACATCAATG-3′, and reverse, 5′-CAAGGTCCCTGCCCTTATCA-3′; *Ifnb1* forward, 5′-AGCTCCAAGAAAGGACGAACA-3′, and reverse, 5′-GCCCTGTAGGTGAGGTTGATCT-3′; and *Actb* forward, 5′-GAGTCCTACGACATCATCGCT-3′, and reverse, 5′-CCGACATAGTTTGGGAAACAGT-3′. Relative mRNA expression was calculated using the 2^–ΔΔCt^ method.

### ELISA.

Cytokine concentrations in cell culture supernatants were determined using ELISA kits for TNF-α, IL-1β, IFN-γ, and IFN-β (MTA00B, MLB00C, MIF00, and MIFNB0; R&D Systems). Supernatants were collected after the indicated treatments, clarified by centrifugation, and assayed according to the manufacturer’s instructions.

### Fundus imaging.

Fundus photographs were obtained from 8-week-old mice before STZ administration using a Micron IV fundus camera for small animals (Phoenix Research Labs) after pupil dilation and anesthesia.

### ERG.

ERG was performed 6 months after STZ induction. After overnight dark adaptation, mice were anesthetized, with pupils dilated and corneas anesthetized before recording. Ground, reference, and corneal contact electrodes were placed in the standard configuration. Recordings were obtained using the Phoenix Ganzfeld System (Phoenix Research Labs) at stimulus intensities of 1.0–4.0 log_10_ cd·s/m^2^. Scotopic a- and b-wave amplitudes were quantified for the indicated groups.

### Retinal trypsin digestion.

Retinal vasculature was isolated using standard trypsin digestion. Whole eyes were fixed in 4% paraformaldehyde, and retinas were removed and incubated in 3% trypsin at 37°C for approximately 30–60 minutes. The vascular networks were mounted on slides and stained using a PAS staining kit (Abcam). Acellular capillaries were quantified by imaging 3 high-magnification fields in each retinal quadrant.

### Evans blue leakage measurement.

Evans blue dye (45 mg/kg) was administered to mice via tail vein injection and allowed to circulate for 2 hours. For qualitative assessment of vascular leakage, a subset of eyes were fixed in 4% paraformaldehyde for 30 minutes, and retinas were then dissected, flat-mounted, and examined by fluorescence microscopy.

For quantitative evaluation, mice were anesthetized and perfused trans-cardially with PBS to remove intravascular dye. Retinas were isolated, dried for 2 hours, and weighed. Evans blue was extracted by incubating each retina in formamide at 70°C overnight. After centrifugation, absorbance was measured at 620 nm using 740 nm as the reference wavelength. Dye concentration was determined from standard curves and normalized to retinal dry weight.

### Cell migration assay.

HRMECs were cultured to approximately 90% confluence, and a linear scratch was introduced using a sterile 200 μL pipette tip. Detached cells were removed by gentle PBS rinsing. Images of the wound area were captured at 0 and 6 hours using an inverted microscope. Gap closure was quantified in ImageJ.

### Tube formation assay.

For in vitro angiogenic analysis, HRMECs (2 × 10^4^ per well) were seeded onto a Matrigel-coated 96-well plate (ibidi). After 6 hours, endothelial network formation was imaged under an inverted microscope. Tube length and branching were quantified using the Angiogenesis Analyzer plug-in in ImageJ.

### Statistics.

Data were analyzed using GraphPad Prism 10.1.1 (GraphPad Software) and are presented as mean ± SD. Normality was assessed using the Kolmogorov-Smirnov test. Comparisons between 2 groups were performed using a 2-tailed unpaired Student’s *t* test. Comparisons among 3 or more groups were performed using 1-way ANOVA followed by Bonferroni’s multiple-comparison test. For CHX chase assays, data were analyzed using ordinary 2-way ANOVA followed by Šidák’s multiple-comparison test. For ERG analyses involving multiple stimulus intensities, data were analyzed using 2-way repeated-measures ANOVA followed by Šidák’s multiple-comparison test. A *P* value < 0.05 was considered statistically significant. Sample sizes (*n*) are provided in the corresponding figure legends.

### Study approval.

All animal procedures were approved by the Shanghai General Hospital Clinical Center Laboratory Animal Welfare and Ethics Committee (approval no. 2024AWS262) and adhered to the ARVO Statement for the Use of Animals in Ophthalmic and Vision Research.

### Data availability.

The scRNA-seq dataset reanalyzed in this study is publicly available in the Gene Expression Omnibus under accession number GSE178121. Data values for all graphs and reported means are provided in the Supporting Data Values file. Additional materials and analytic code are available from the corresponding author upon reasonable request.

## Author contributions

K Liu, TN, and YJ conceived and designed the study. TN and YJ performed experiments and analyzed data. XX, QZ, XL, K Lv, JF, and K Liu contributed to experiments, data acquisition, and analysis. TN and YJ drafted the manuscript. K Liu supervised the study and revised the manuscript. Author order among the co–first authors was determined by the extent of their contributions. All authors reviewed, edited, and approved the final manuscript.

## Conflict of interest

The authors have declared that no conflict of interest exists.

## Funding support

- National Natural Science Foundation of China (82201211 to TN and 82471109 to K Liu).

## Supplementary Material

Supplemental data

Unedited blot and gel images

Supporting data values

## Figures and Tables

**Figure 1:**
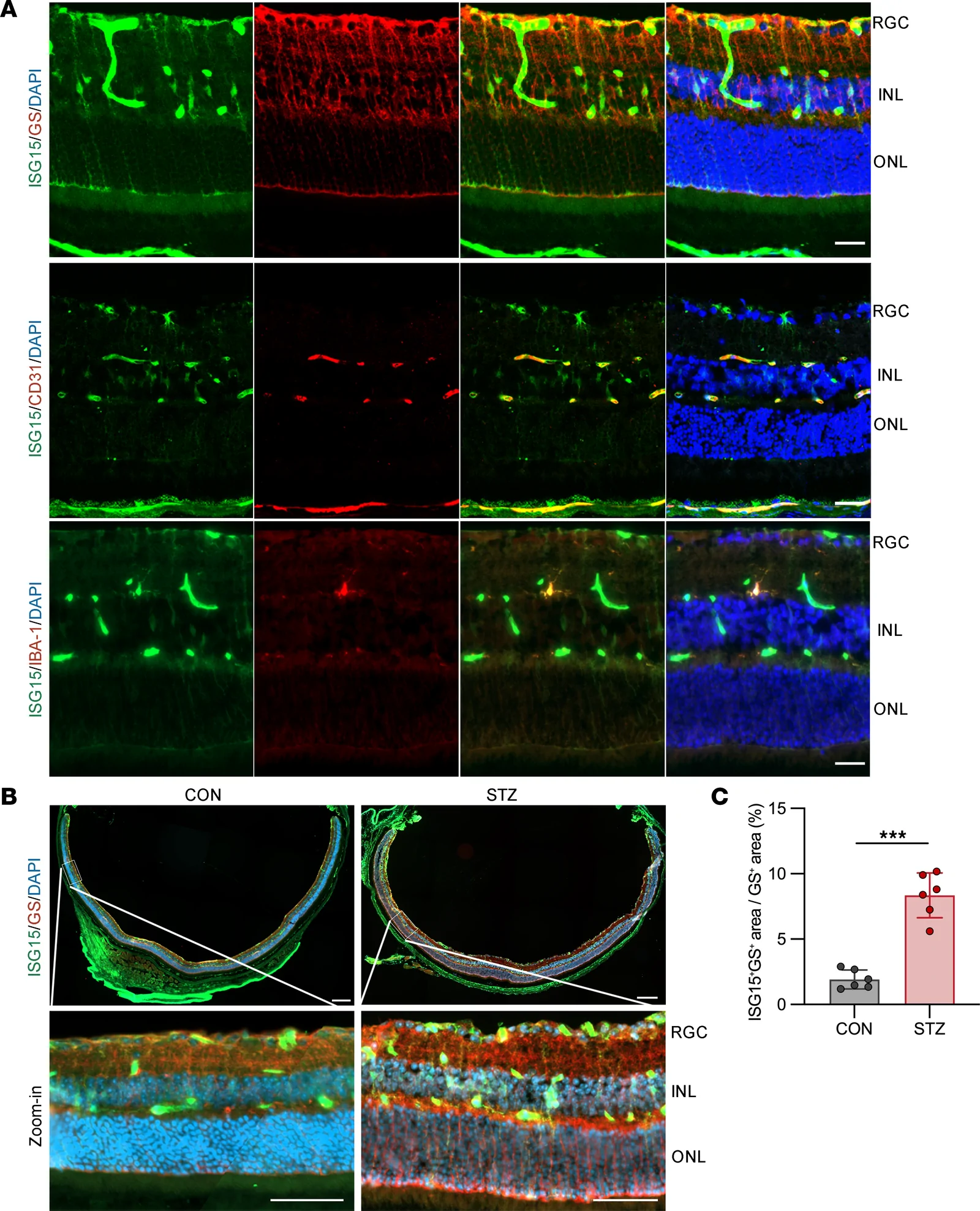
Retinal localization and quantification of ISG15^+^ Müller-associated signals in retinas analyzed 6 months after STZ induction. (**A**) Representative retinal sections costained for ISG15 (green) with the Müller glial marker GS (red, top), the endothelial marker CD31 (red, middle), or the microglia marker IBA-1 (red, bottom); nuclei were counterstained with DAPI (blue). Scale bars: 25 μm. Images are representative of 3 independent experiments. (**B**) Low-magnification retinal sections from CON and STZ-induced diabetic mice stained for ISG15 (green) and GS (red) with DAPI (blue), with corresponding higher-magnification views (Zoom-in) showing the distribution of ISG15 signals within the GS^+^ Müller glial compartment. Scale bars: 200 μm (low magnification), 25 μm (zoom). (**C**) Quantification of ISG15^+^GS^+^ area/GS^+^ area (%) in CON and STZ retinas (*n* = 6). Data are presented as the mean ± SD. *P* values were determined by 2-tailed unpaired Student’s *t* test. ***P < 0.001. CON, control; RGC, retinal ganglion cell layer; INL, inner nuclear layer; ONL, outer nuclear layer.

**Figure 2:**
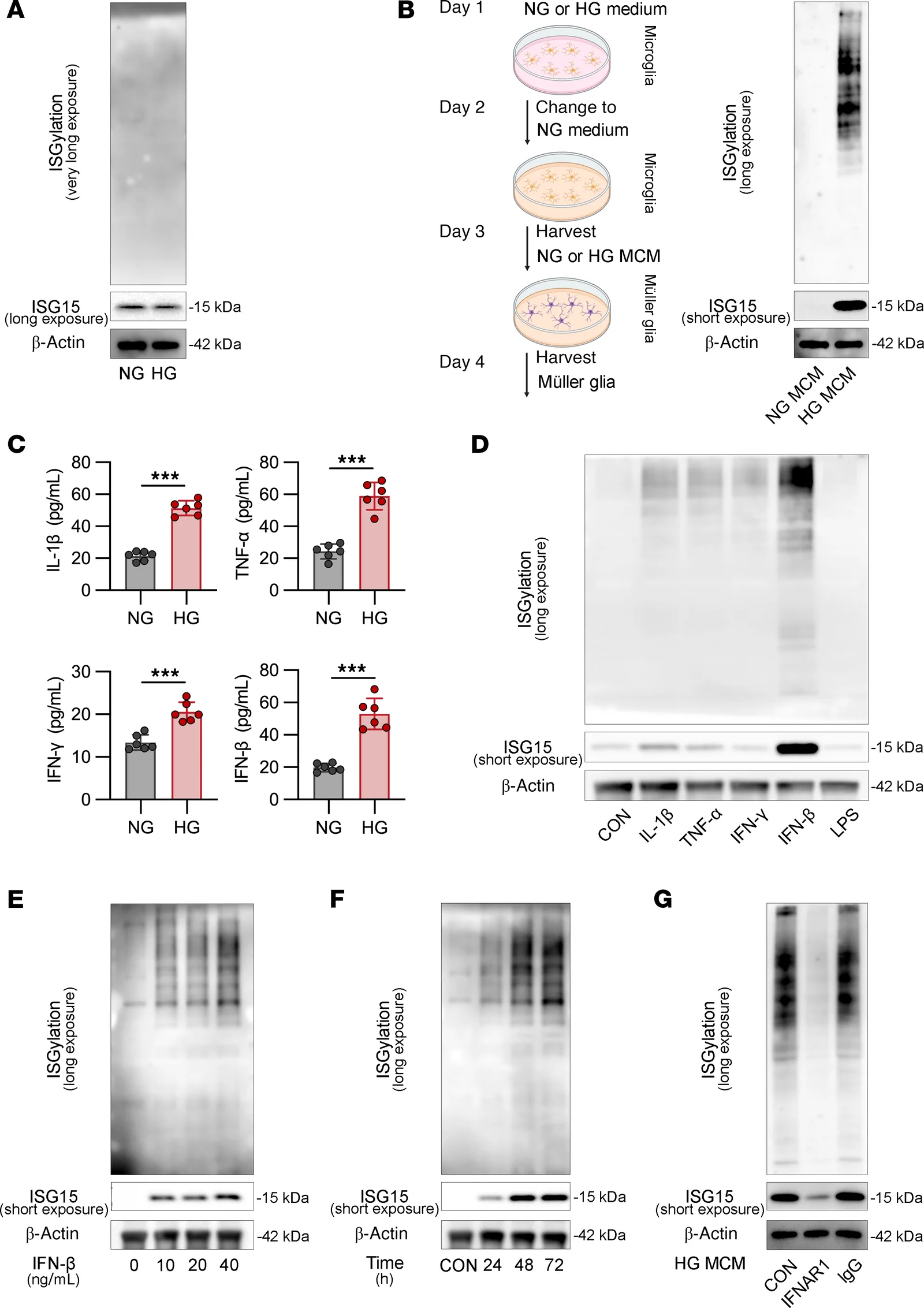
Microglia-derived IFN-β induces ISG15 expression and ISGylation in Müller glia. (**A**) Immunoblot analysis of free ISG15 and ISGylated proteins in primary Müller glia cultured under NG or HG. (**B**) Workflow for NG-/HG-MCM generation and Müller glial stimulation, created using FigDraw (https://www.figdraw.com/#/). (**C**) ELISA quantification of IL-1β, IFN-β, IFN-γ, and TNF-α in microglial supernatants under NG versus HG conditions (*n* = 6). (**D**–**F**) Immunoblots of ISG15/ISGylation in Müller glia treated with candidate cytokines or LPS (**D**), IFN-β doses (**E**), or IFN-β durations (**F**). (**G**) Immunoblot analysis of ISG15/ISGylation in Müller glia treated with HG-MCM in the presence of IFNAR1 blockade or IgG control. Exposure settings are indicated in the blot panels; immunoblots in **A** and **D**–**G** are representative of 3 independent experiments. Data are presented as the mean ± SD. *P* values were determined by 2-tailed unpaired Student’s *t* test. ***P < 0.001. CON, control.

**Figure 3:**
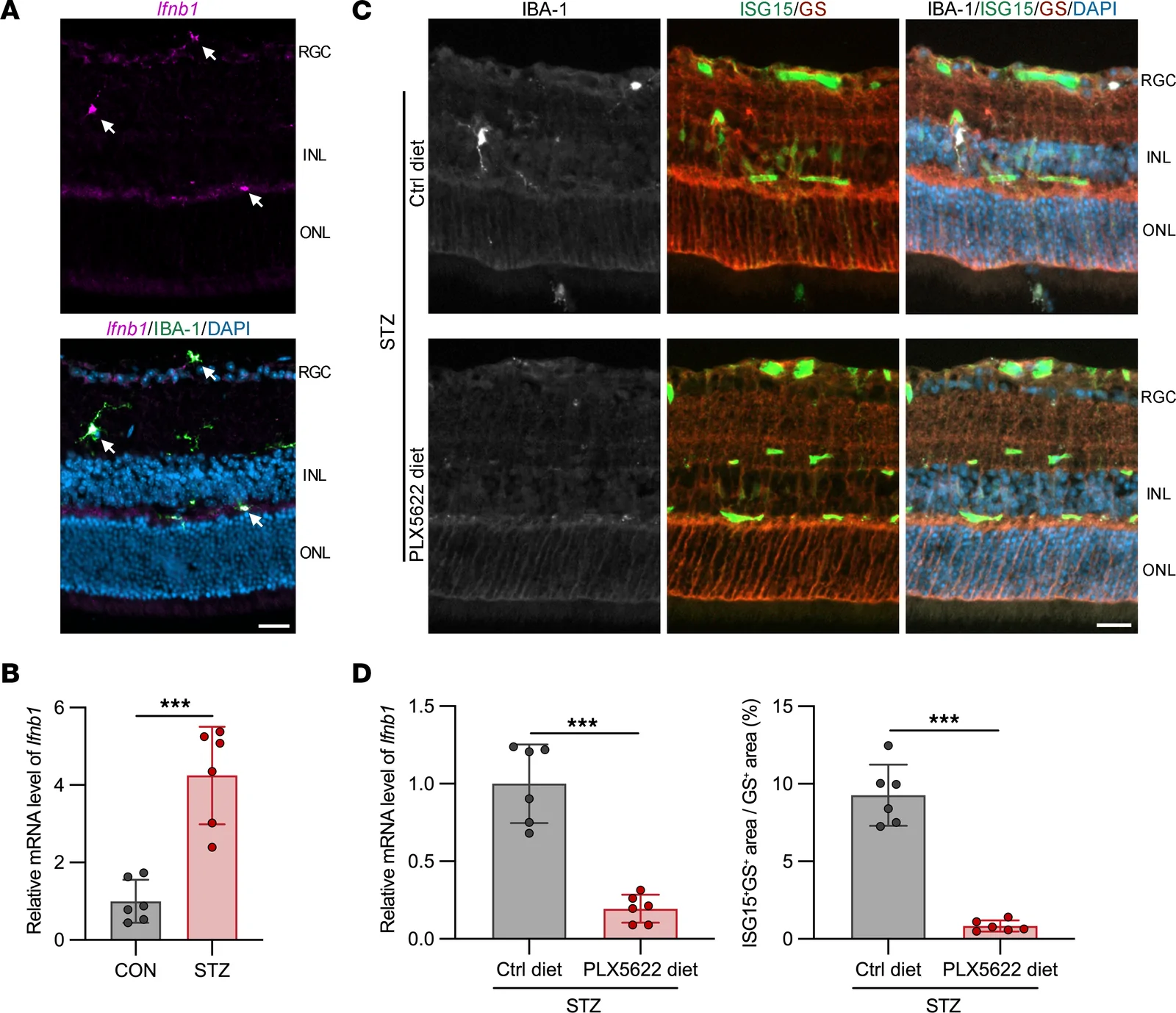
Microglia-derived IFN-β promotes Müller-associated ISG15 induction in diabetic retinas. (**A**) Representative *Ifnb1* RNAscope with IBA-1 immunostaining in STZ-diabetic retina, showing *Ifnb1* mRNA signals (magenta), IBA-1 (green), and DAPI (blue). Arrows indicate *Ifnb1* signals associated with IBA-1^+^ microglial cells. Scale bar: 25 μm. Images are representative of 3 independent experiments. (**B**) RT-qPCR analysis of retinal *Ifnb1* mRNA expression in CON and STZ mice (*n* = 6). (**C**) Representative STZ retinal sections after control or PLX5622 diet, stained for IBA-1 (gray), ISG15 (green), GS (red), and DAPI (blue). Scale bar: 25 μm. (**D**) Quantification of retinal *Ifnb1* mRNA expression (left) and ISG15^+^GS^+^ area/GS^+^ area (%) (right) in STZ-diabetic mice fed control or PLX5622-containing diet (*n* = 6). Data are presented as the mean ± SD. *P* values were determined by 2-tailed unpaired Student’s *t* test. ***P < 0.001. CON, control; RGC, retinal ganglion cell layer; INL, inner nuclear layer; ONL, outer nuclear layer.

**Figure 4:**
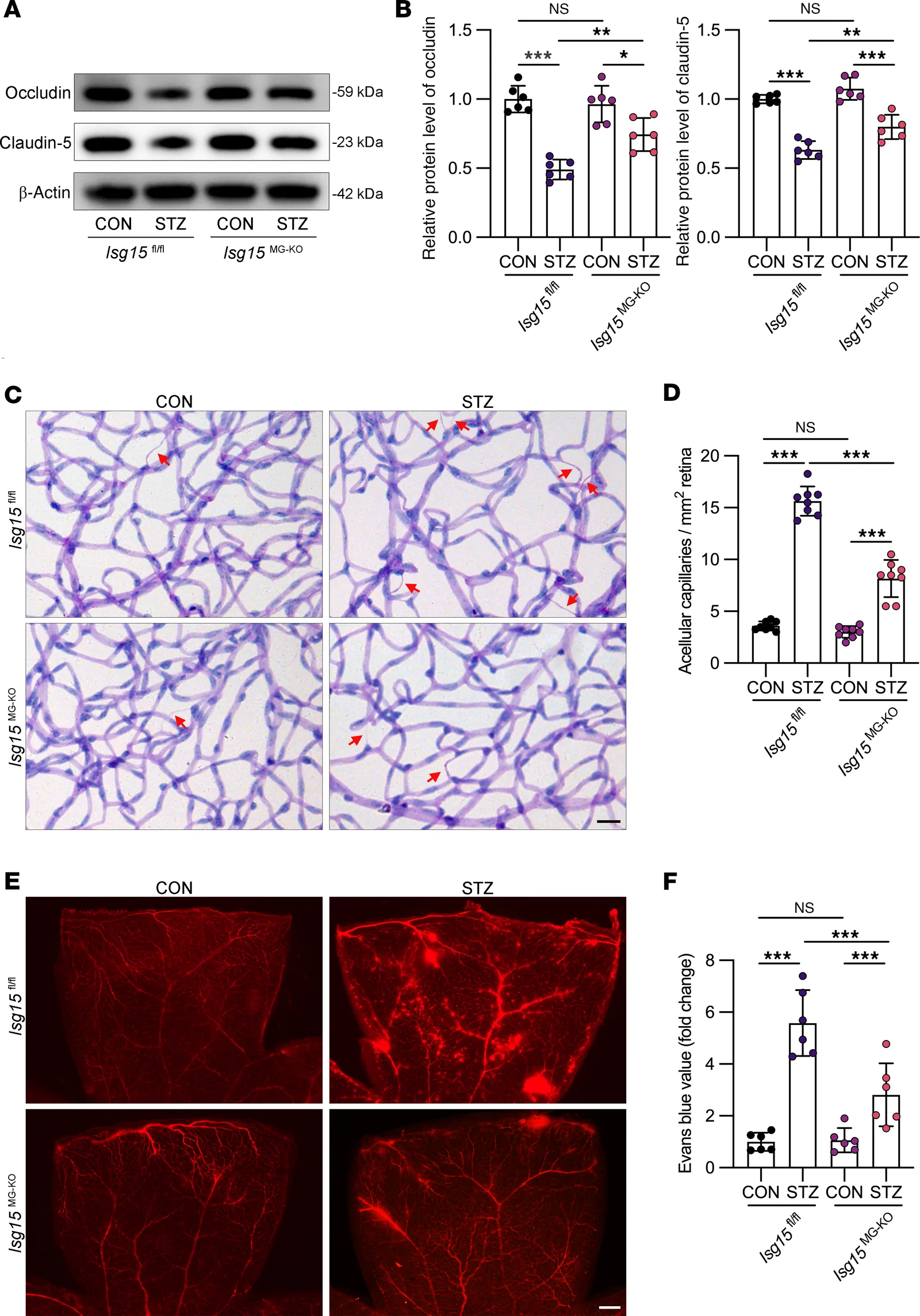
Müller glia–specific ISG15 deletion preserves retinal vascular barrier integrity and microvascular structure in retinas analyzed 6 months after STZ induction. (**A**) Immunoblot analysis of tight junction proteins Occludin and Claudin-5 in retinas from *Isg15*^fl/fl^ and *Isg15*^MG-KO^ mice under CON or STZ-induced diabetic conditions. (**B**) Densitometric quantification of Occludin and Claudin-5 protein levels (*n* = 6). (**C**) Representative images of retinal trypsin digests showing acellular capillaries (arrows) in *Isg15*^fl/fl^ and *Isg15*^MG-KO^ mice under CON or STZ conditions. Scale bar: 25 μm. (**D**) Quantification of acellular capillaries per retinal area (*n* = 8). (**E**) Representative Evans blue assay images from *Isg15*^fl/fl^ and *Isg15*^MG-KO^ mice under CON or STZ conditions. Scale bar: 200 μm. (**F**) Quantification of Evans blue extravasation (*n* = 6). Immunoblots were normalized to β-actin as the loading control. Data are presented as the mean ± SD. *P* values were determined by 1-way ANOVA with Bonferroni’s multiple-comparison test. *P < 0.05, **P < 0.01, ***P < 0.001. CON, control.

**Figure 5:**
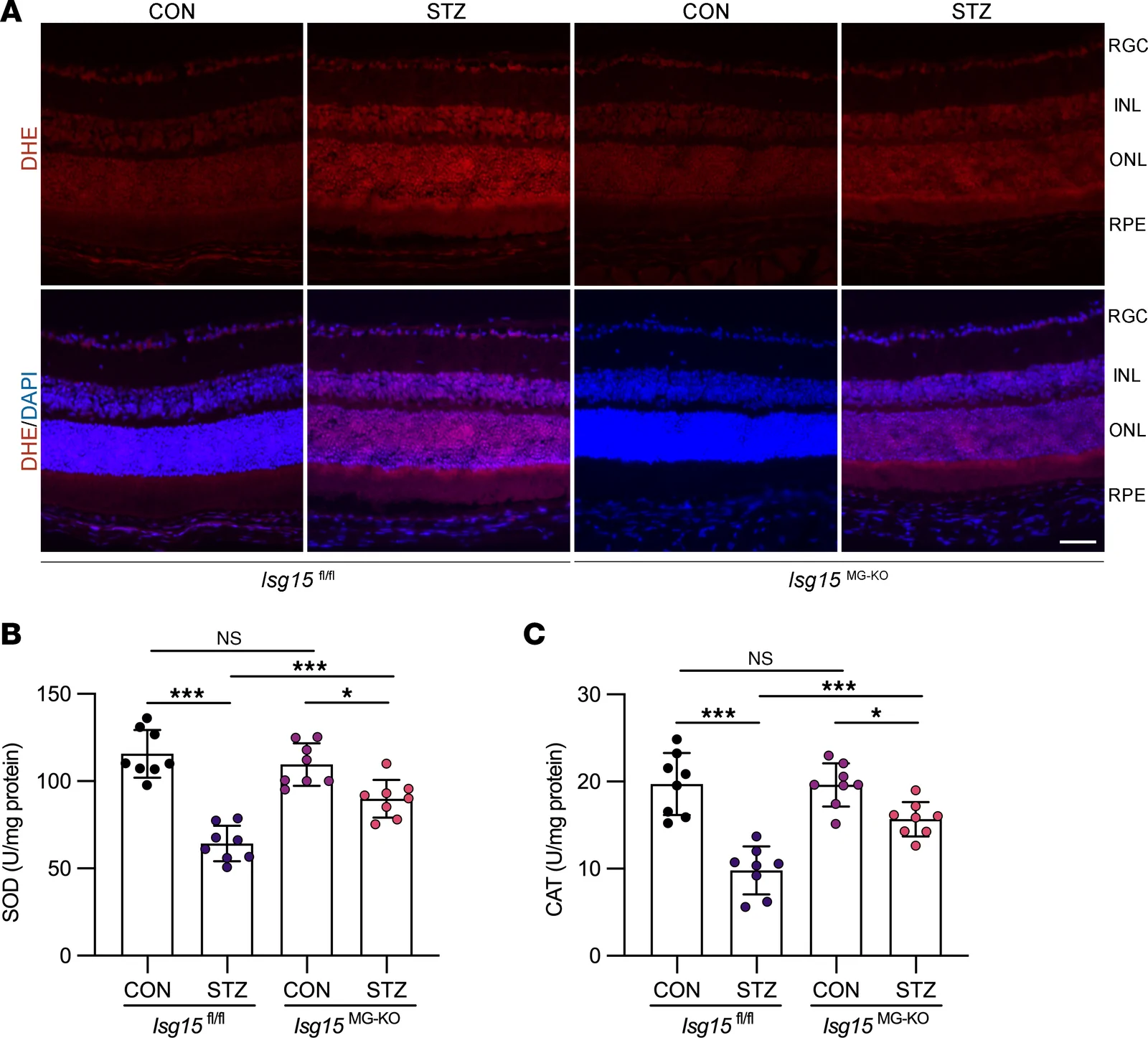
Müller glia–specific ISG15 deletion attenuates diabetes-induced retinal oxidative stress. (**A**) Representative retinal sections stained with DHE (red) to visualize superoxide-associated oxidative stress, with nuclei counterstained by DAPI (blue), in *Isg15*^fl/fl^ and *Isg15*^MG-KO^ mice under CON or STZ conditions. Scale bar: 50 μm. Images are representative of 3 independent experiments. (**B** and **C**) Quantification of SOD (**B**) and CAT (**C**) activity in retinal tissue (*n* = 8). Data are presented as the mean ± SD. *P* values were determined by 1-way ANOVA with Bonferroni’s multiple-comparison test. *P < 0.05, ***P < 0.001. CON, control; RGC, retinal ganglion cell layer; INL, inner nuclear layer; ONL, outer nuclear layer; RPE, retinal pigment epithelium.

**Figure 6:**
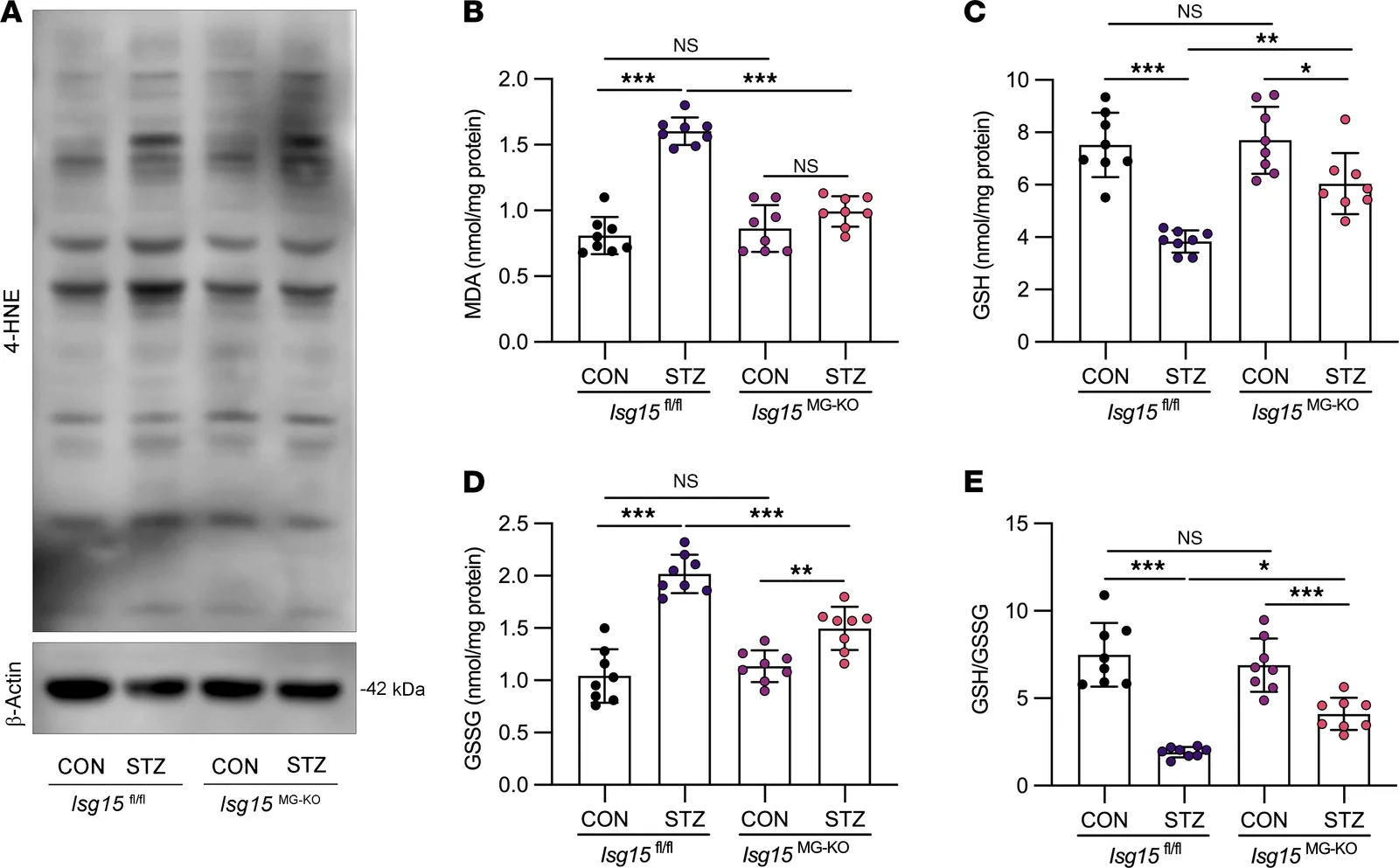
Müller glia–specific ISG15 deletion attenuates retinal lipid peroxidation and glutathione dysregulation in diabetes. (**A**) Representative immunoblot analysis of 4-HNE–modified proteins in retinal lysates from *Isg15*^fl/fl^ and *Isg15*^MG-KO^ mice under CON or STZ conditions. Immunoblots are representative of 3 independent experiments. (**B**–**E**) Quantification of MDA (**B**), GSH (**C**), GSSG (**D**), and the GSH/GSSG ratio (**E**) in retinal tissue (*n* = 8). Data are presented as the mean ± SD. *P* values were determined by 1-way ANOVA with Bonferroni’s multiple-comparison test. *P < 0.05, **P < 0.01, ***P < 0.001. CON, control.

**Figure 7:**
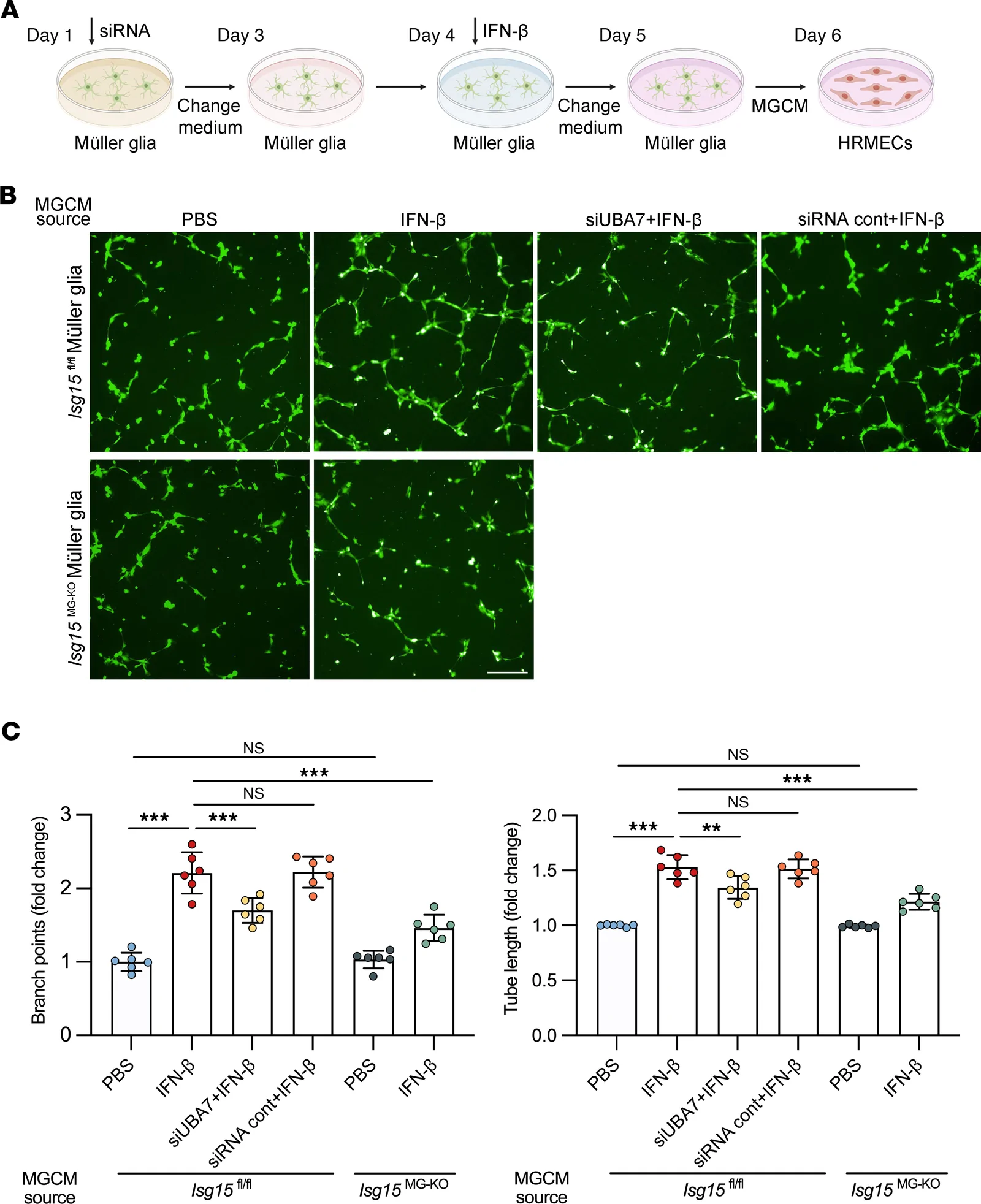
ISG15/ISGylation in Müller glia promotes endothelial tube formation in vitro. (**A**) Schematic of the serum-free MGCM generation workflow and subsequent HRMEC functional assays, created using FigDraw (https://www.figdraw.com/#/). (**B**) Representative images of tube formation by HRMECs after exposure to MGCM derived from the indicated Müller glia conditions. Scale bar: 250 μm. (**C**) Quantification of branch points (left) and total tube length (right) (*n* = 6). Data are presented as the mean ± SD. *P* values were determined by 1-way ANOVA with Bonferroni’s multiple-comparison test. **P < 0.01, ***P < 0.001.

**Figure 8:**
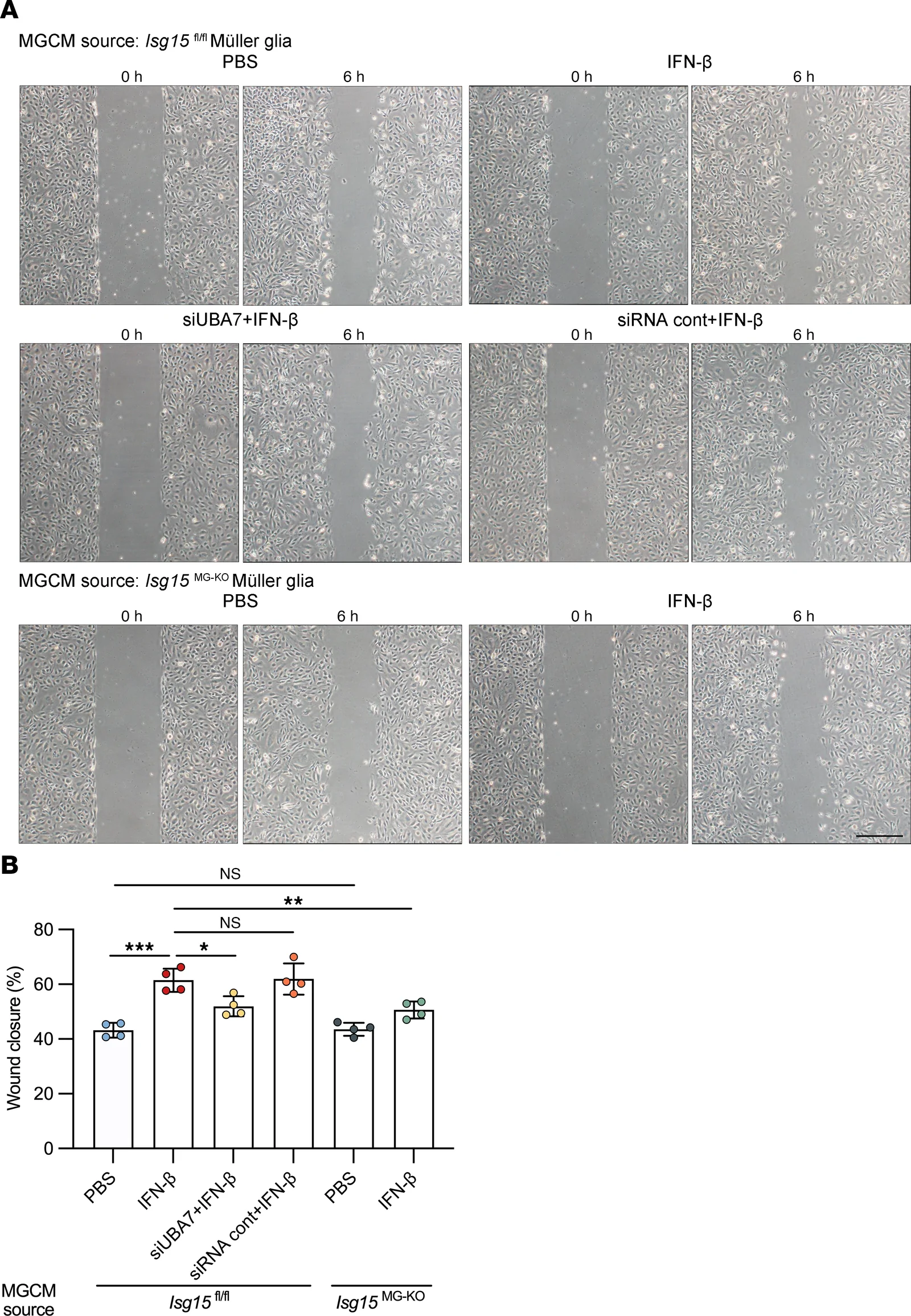
ISG15/ISGylation in Müller glia promotes endothelial migration in vitro. (**A**) Representative scratch-wound migration images of HRMECs at 0 and 6 hours following exposure to the indicated MGCM conditions. Scale bar: 200 μm. (**B**) Quantification of HRMEC wound closure (*n* = 4). Data are presented as the mean ± SD. *P* values were determined by 1-way ANOVA with Bonferroni’s multiple-comparison test. *P < 0.05, **P < 0.01, ***P < 0.001.

**Figure 9:**
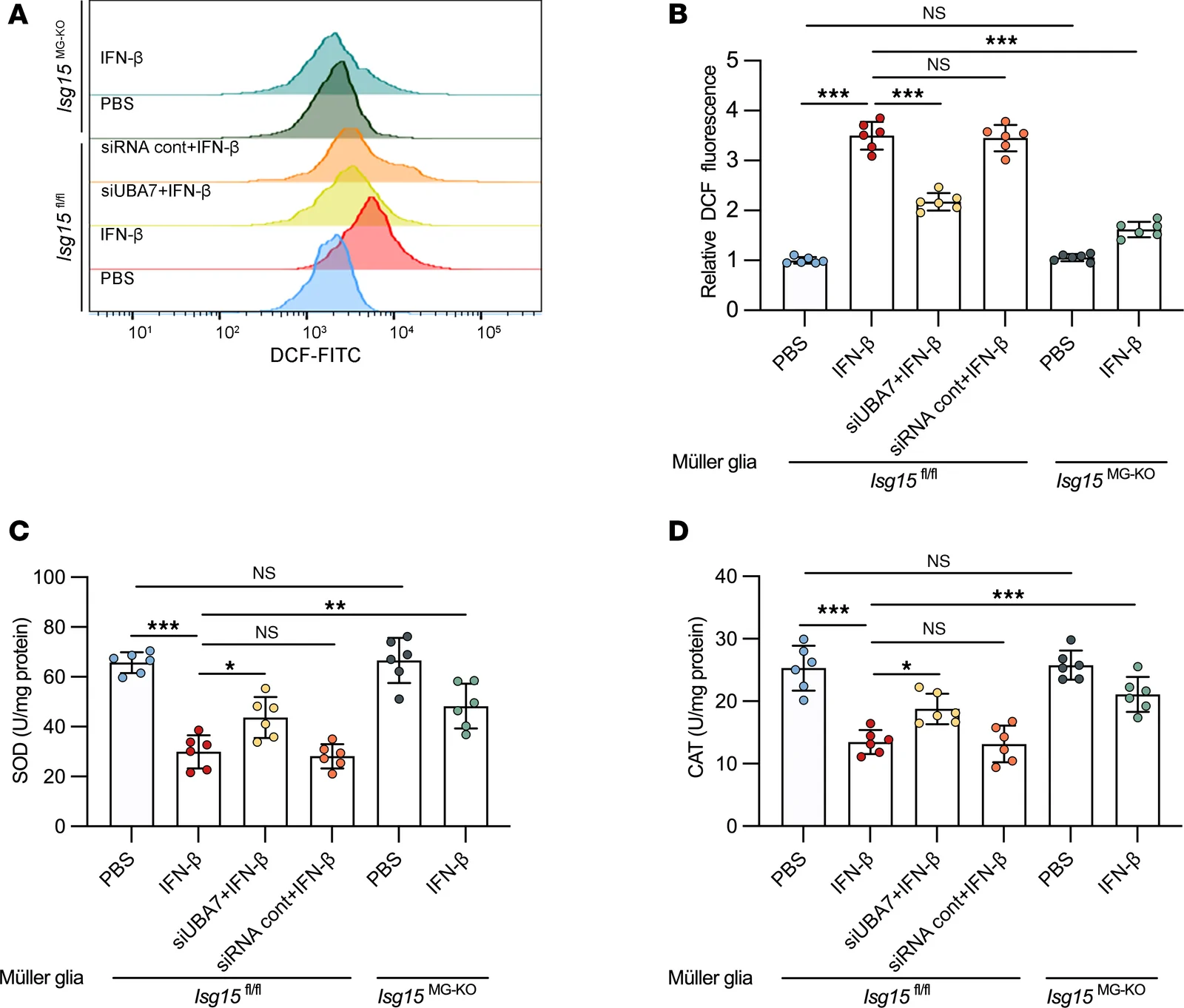
ISG15/ISGylation promotes IFN-β–induced oxidative stress in primary Müller glia. (**A**) Representative flow cytometry histograms of DCF fluorescence in primary Müller glia under the indicated conditions. (**B**–**D**) Quantification of relative DCF fluorescence intensity (**B**), SOD activity (**C**), and CAT activity (**D**) in primary Müller glia under the indicated conditions (*n* = 6). Data are presented as the mean ± SD. *P* values were determined by 1-way ANOVA with Bonferroni’s multiple-comparison test. *P < 0.05, **P < 0.01, ***P < 0.001. DCF, 2′,7′-dichlorofluorescein.

**Figure 10:**
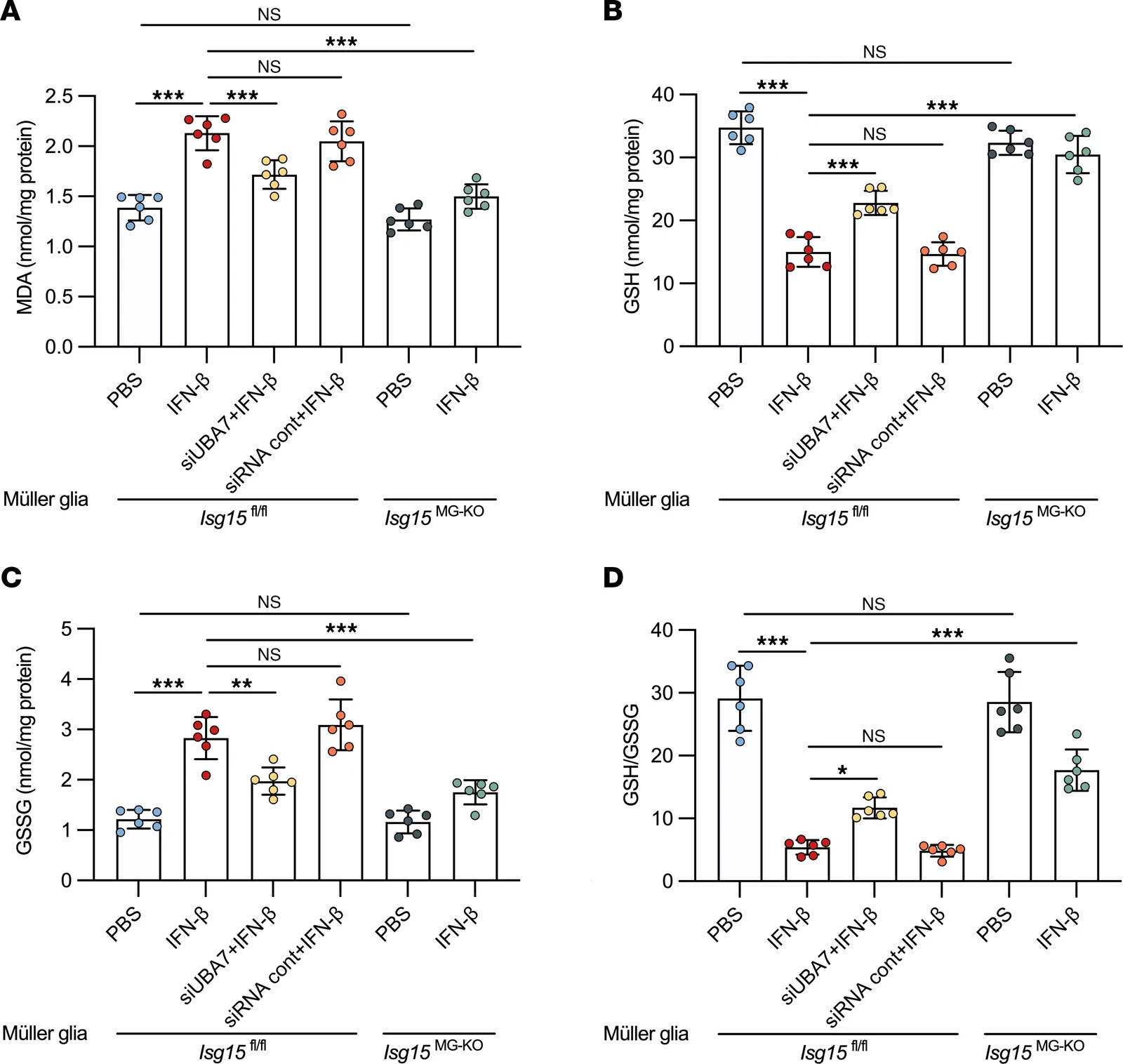
ISG15/ISGylation promotes IFN-β–induced lipid peroxidation and glutathione redox imbalance in primary Müller glia. (**A**–**D**) Quantification of MDA (**A**), GSH (**B**), GSSG (**C**), and the GSH/GSSG ratio (**D**) in primary Müller glia under the indicated conditions (*n* = 6). Data are presented as the mean ± SD. *P* values were determined by 1-way ANOVA with Bonferroni’s multiple-comparison test. *P < 0.05, **P < 0.01, ***P < 0.001.

**Figure 11:**
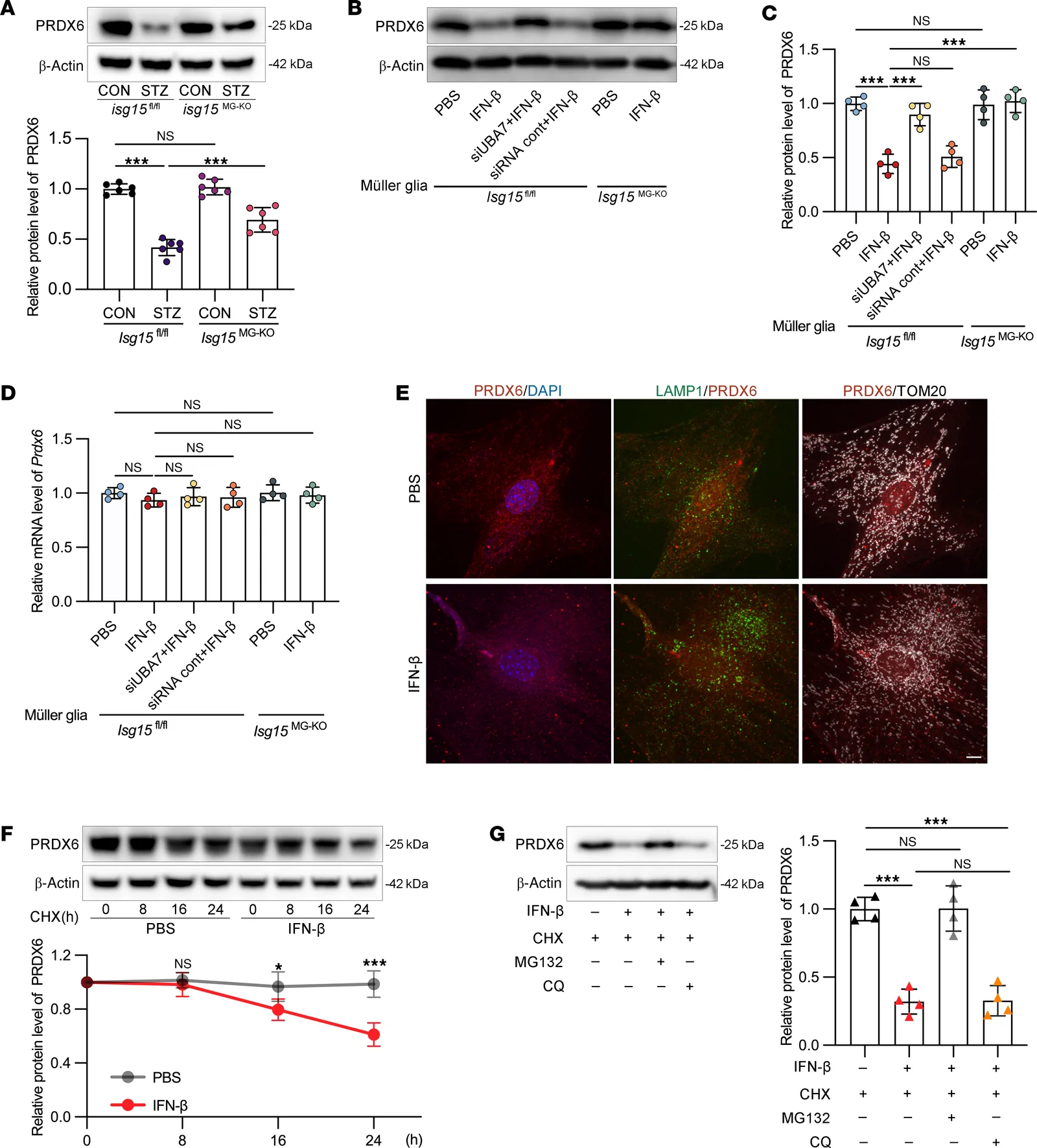
ISG15/ISGylation promotes proteasome-dependent degradation of PRDX6 in Müller glia. (**A**) Immunoblot analysis and quantification of retinal PRDX6 in *Isg15*^fl/fl^ and *Isg15*^MG-KO^ mice under CON or STZ conditions (*n* = 6). (**B**) Immunoblot analysis of PRDX6 in primary Müller glia under the indicated conditions. (**C**) Quantification of PRDX6 protein expression in **B** (*n* = 4). (**D**) RT-qPCR analysis of *Prdx6* mRNA in primary Müller glia under the indicated conditions (*n* = 4). (**E**) Immunofluorescence staining showing subcellular localization of PRDX6 in primary Müller glia under PBS or IFN-β conditions, with costaining for TOM20 (mitochondria marker; gray), LAMP1 (lysosome marker; green), and DAPI (blue). Scale bar: 10 μm. Images are representative of 3 independent experiments. (**F**) CHX chase assay of PRDX6 protein stability following PBS or IFN-β treatment, with representative immunoblots and quantification (*n* = 4). (**G**) Immunoblot analysis and quantification of PRDX6 after treatment with IFN-β and CHX in the presence or absence of MG132 or CQ (*n* = 4). Immunoblots were normalized to β-actin. Data are presented as the mean ± SD. *P* values were determined by 1-way ANOVA with Bonferroni’s multiple-comparison test, except for the CHX chase assay in **F**, which was analyzed by 2-way ANOVA with Šidák’s multiple-comparison test. ***P < 0.001. CON, control; MG132, proteasome inhibitor; LAMP1, lysosome-associated membrane protein 1; PRDX6, peroxiredoxin 6; TOM20, translocase of outer mitochondrial membrane 20.

**Figure 12:**
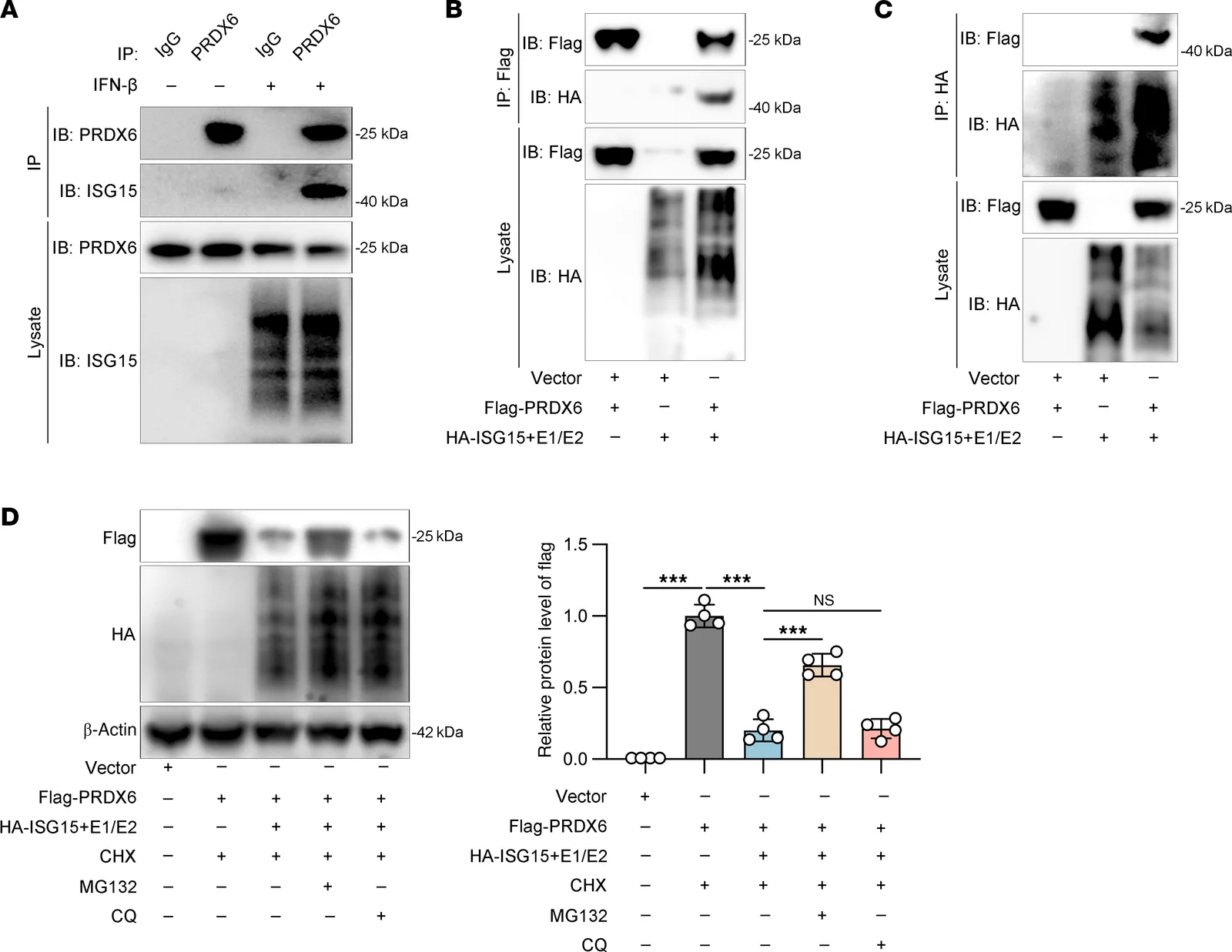
PRDX6 ISGylation promotes its proteasome-dependent degradation. (**A**) Endogenous co-IP analysis of PRDX6-associated ISG15/ISGylation in primary Müller glia under the indicated conditions. (**B**) Co-IP analysis of FLAG-PRDX6–associated HA-ISG15/ISGylation signals in HEK293T cells expressing the indicated components. (**C**) Reciprocal co-IP analysis of HA-ISG15/ISGylation–associated FLAG-PRDX6 in HEK293T cells. (**D**) Immunoblot analysis and quantification of FLAG-PRDX6 in a reconstituted ISGylation system containing HA-ISG15, E1, and E2 in HEK293T cells following CHX treatment, in the presence or absence of MG132 or CQ (*n* = 4). E1 and E2 correspond to UBA7 and UBE2L6, respectively. Co-IP experiments in **A**–**C** were independently repeated 3 times with similar results. Data are presented as the mean ± SD. *P* values were determined by 1-way ANOVA with Bonferroni’s multiple-comparison test. ***P < 0.001. MG132, proteasome inhibitor; PRDX6, peroxiredoxin 6.

## Version 1 10/15/2026

Electronic publication
