# Supplementary material for: ISG15+ Müller glial subtype contributes to early blood–retinal barrier breakdown in diabetic retinopathy
Source: J Clin Invest. 2026 Oct 15;136(20):e205800. doi: 10.1172/JCI205800 (PMC13668631; doi:10.1172/JCI205800)
Supplement: Supplementary file 1 [file jci-136-205800-s013.pdf]

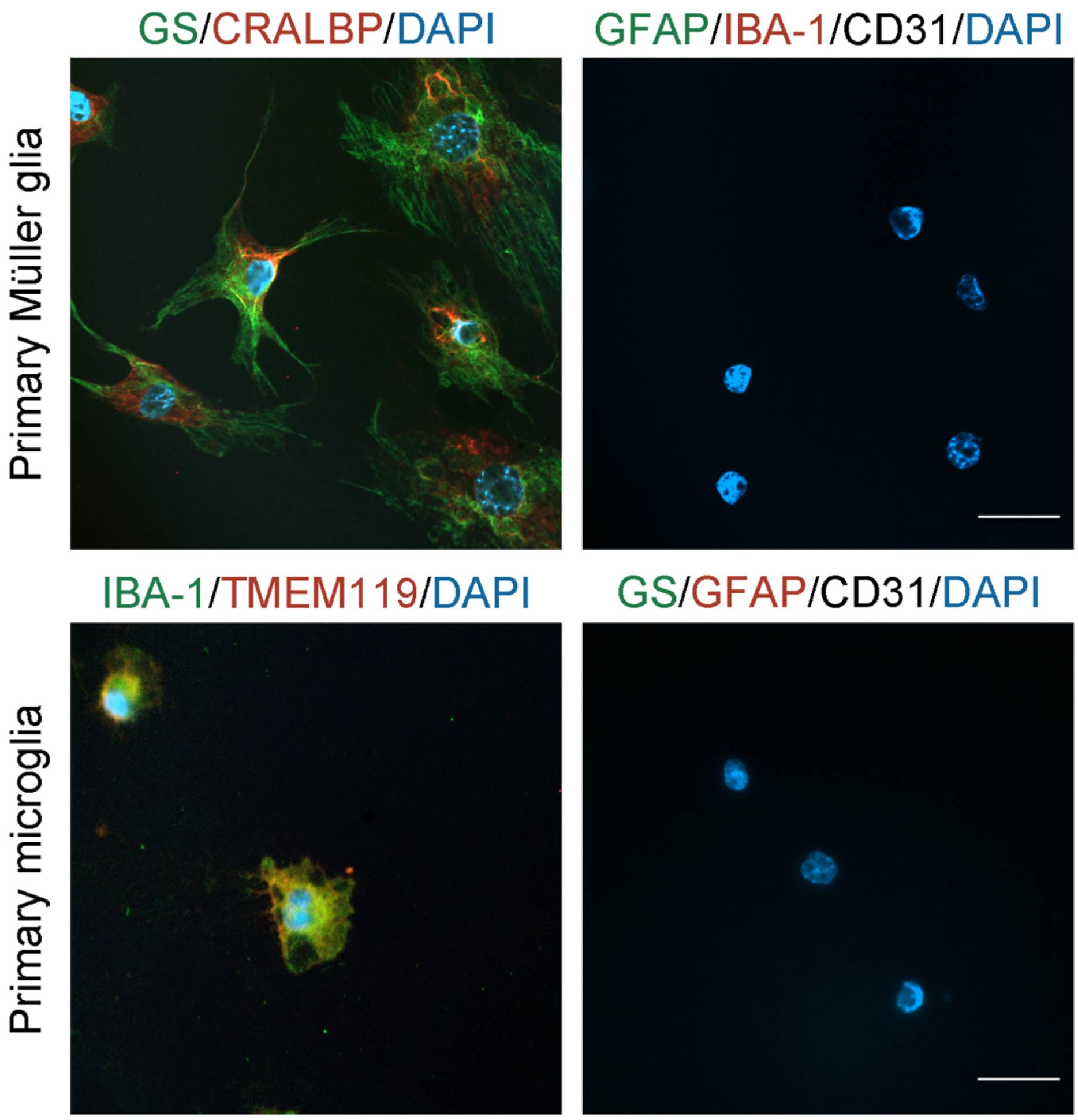

Supplemental Figure 1. Characterization of primary retinal glial cultures used for in vitro experiments.

Representative immunocytochemistry of primary Müller glial cultures showing GS (green), CRALBP (red), and DAPI (blue), with minimal staining for GFAP (green), IBA-1 (red), or CD31 (gray). Representative immunocytochemistry of primary retinal microglial cultures showing IBA-1 (green), TMEM119 (red), and DAPI (blue), with minimal staining for GS (green), GFAP (red), or CD31 (gray). Scale bars: 50 µm for Müller glial cultures and 25 µm for retinal microglial cultures. Representative images are from 3 independent experiments.

Abbreviations: CRALBP, cellular retinaldehyde-binding protein; GS, glutamine synthetase; GFAP, glial fibrillary acidic protein; IBA-1, ionized calcium-binding adapter molecule 1; TMEM119, transmembrane protein 119.

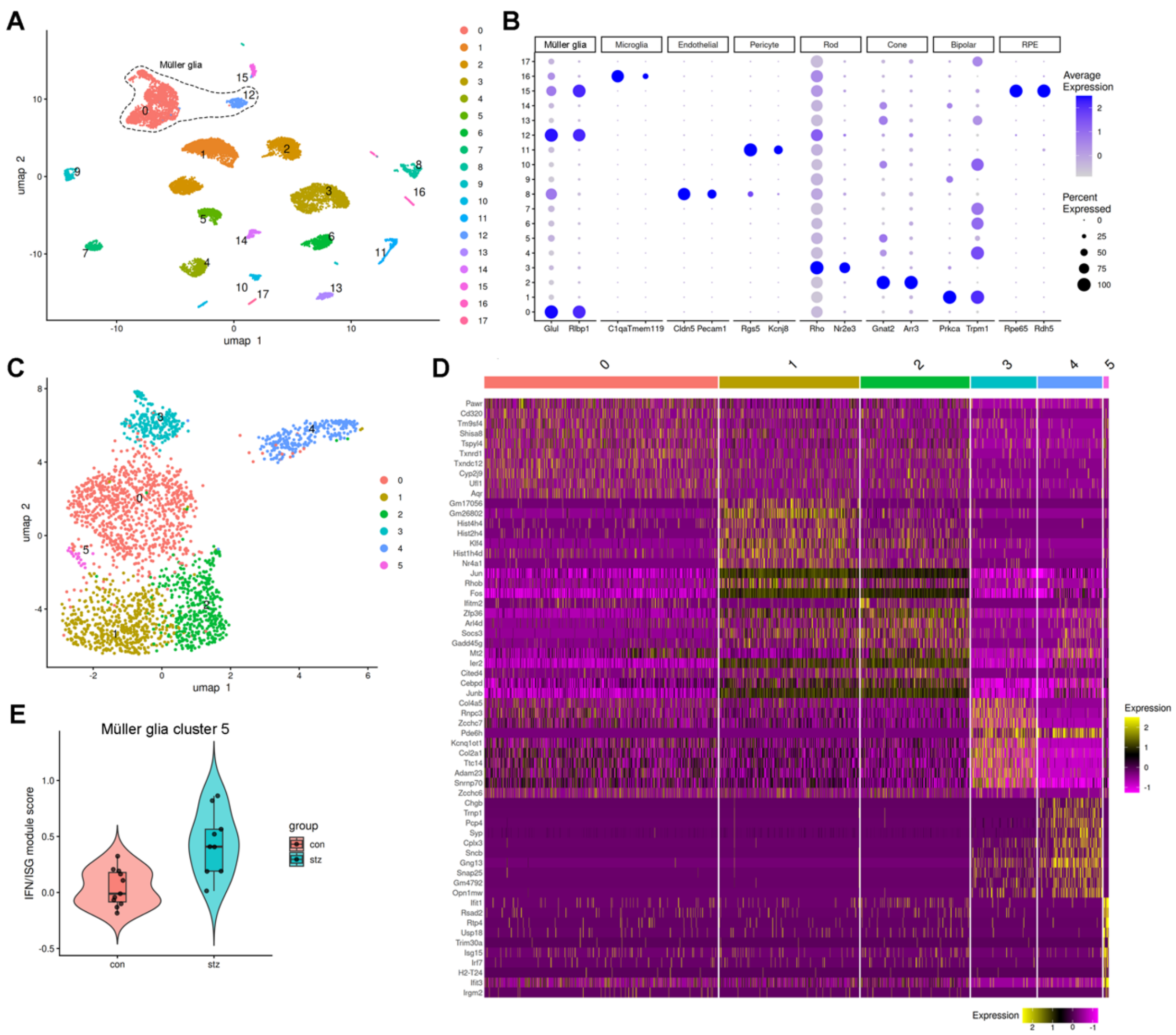

Supplemental Figure 2. Single-cell RNA-sequencing reanalysis identifies an IFN/ISG-enriched Müller glial subcluster in diabetic retinas.

(A) UMAP visualization of retinal cells from a published single-cell RNA-sequencing dataset of control and STZ-diabetic mouse retinas. Parent clusters 0 and 12 were annotated as Müller glia and selected for focused reclustering.

(B) Dot plot showing canonical marker genes used to annotate major retinal cell populations. Dot size indicates the percentage of cells expressing each gene, and color intensity indicates average expression.

(C) UMAP visualization of the focused Müller glial reclustering, resolving six Müller subclusters.

(D) Heatmap showing representative marker genes across Müller glial subclusters. Cluster 5 was enriched for interferon-stimulated and antiviral-response genes, including *Ifit1, Rsad2, Usp18, Isg15, Irf7,* and *Ifit3*.

(E) IFN/ISG module scores in Müller glial cluster 5 from control and STZ-diabetic retinas.

Abbreviations: CON, control; STZ, streptozotocin; UMAP, uniform manifold approximation and projection; IFN, interferon; ISG, interferon-stimulated gene; RPE, retinal pigment epithelium.

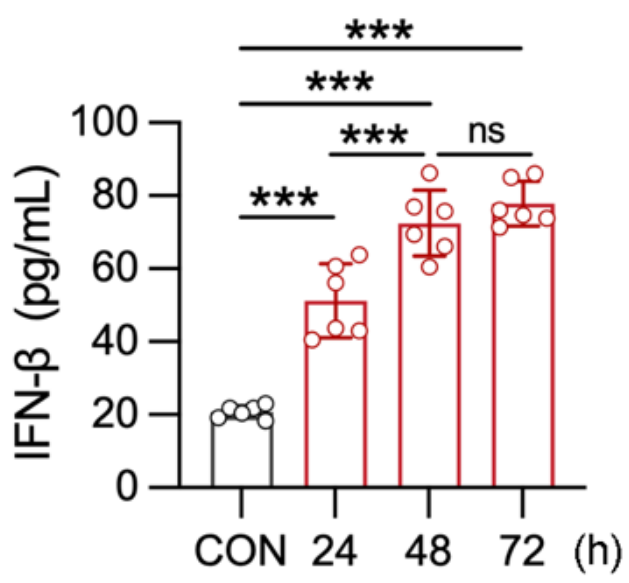

Supplemental Figure 3. Time-course analysis of IFN-β release from primary retinal microglia under HG conditions.

IFN-β concentrations in supernatants from primary retinal microglia cultured under HG conditions for 24, 48, or 72 h were measured by ELISA (n = 6). CON indicates the normal-glucose control condition.

Statistics: Data are mean ± SD. *P* values were determined by one-way ANOVA with Bonferroni's multiple-comparisons test. Significance as indicated (**P* < 0.05, ***P* < 0.01, ****P* < 0.001; ns, not significant).

Abbreviations: CON, control; ELISA, enzyme-linked immunosorbent assay; HG, high glucose.

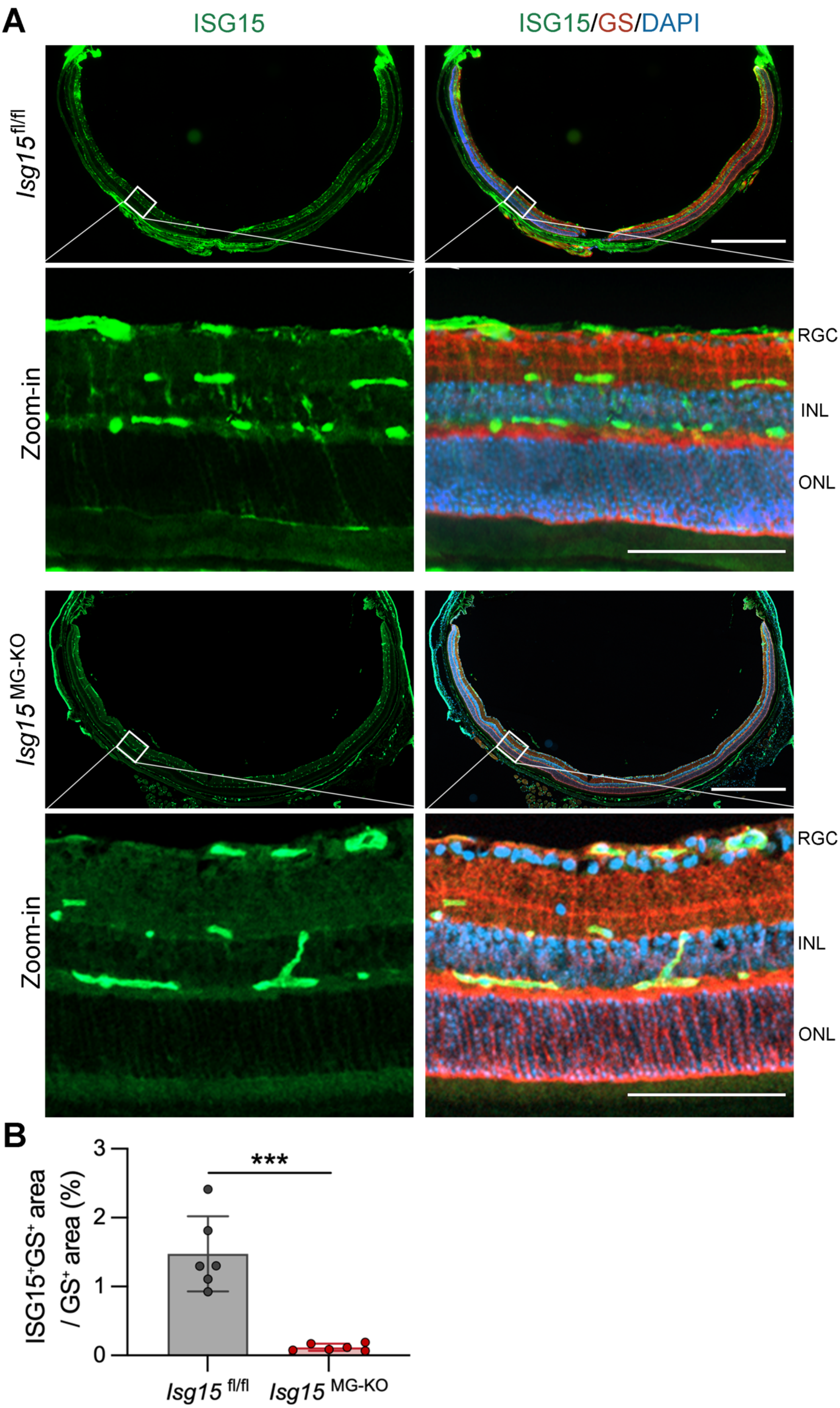
A
ISG15
ISG15/GS/DAPI
Isg15 fl/fl
Zoom-in
RGC
INL
ONL
Isg15 MG-KO
Zoom-in
RGC
INL
ONL
B
ISG15+GS+ area / GS+ area (%)
3
2
1
0
***
Isg15 fl/fl
Isg15 MG-KO

Supplemental Figure 4. Validation of Müller glia–specific ISG15 deletion in *Isg15*$^{\text{MG-KO}}$ retinas.

(A) Representative retinal sections from 8-week-old *Isg15*$^{\text{fl/fl}}$ and *Isg15*$^{\text{MG-KO}}$ mice before STZ administration, co-stained for ISG15 (green) and the Müller glial marker GS (red), with nuclei counterstained by DAPI (blue). Low-magnification images show the overall retinal distribution of ISG15, and boxed regions are shown at higher magnification ("Zoom-in") to show ISG15 signal in relation to the GS^{+} Müller glial compartment. Scale bars: 200 µm (low magnification), 100 µm (zoom-in).

(B) Quantification of the Müller-associated ISG15-positive compartment, expressed as ISG15^{+}GS^{+} area / GS^{+} area (%), in *Isg15*$^{\text{fl/fl}}$ and *Isg15*$^{\text{MG-KO}}$ retinas (n = 6).

Statistics: Data are mean ± SD. *P* values were determined by 2-tailed unpaired Student's t-test. Significance as indicated (*$P < 0.05$, **$P < 0.01$, ***$P < 0.001$; ns, not significant).

Abbreviations: STZ, streptozotocin; GS, glutamine synthetase; RGC, retinal ganglion cell layer; INL, inner nuclear layer; ONL, outer nuclear layer.

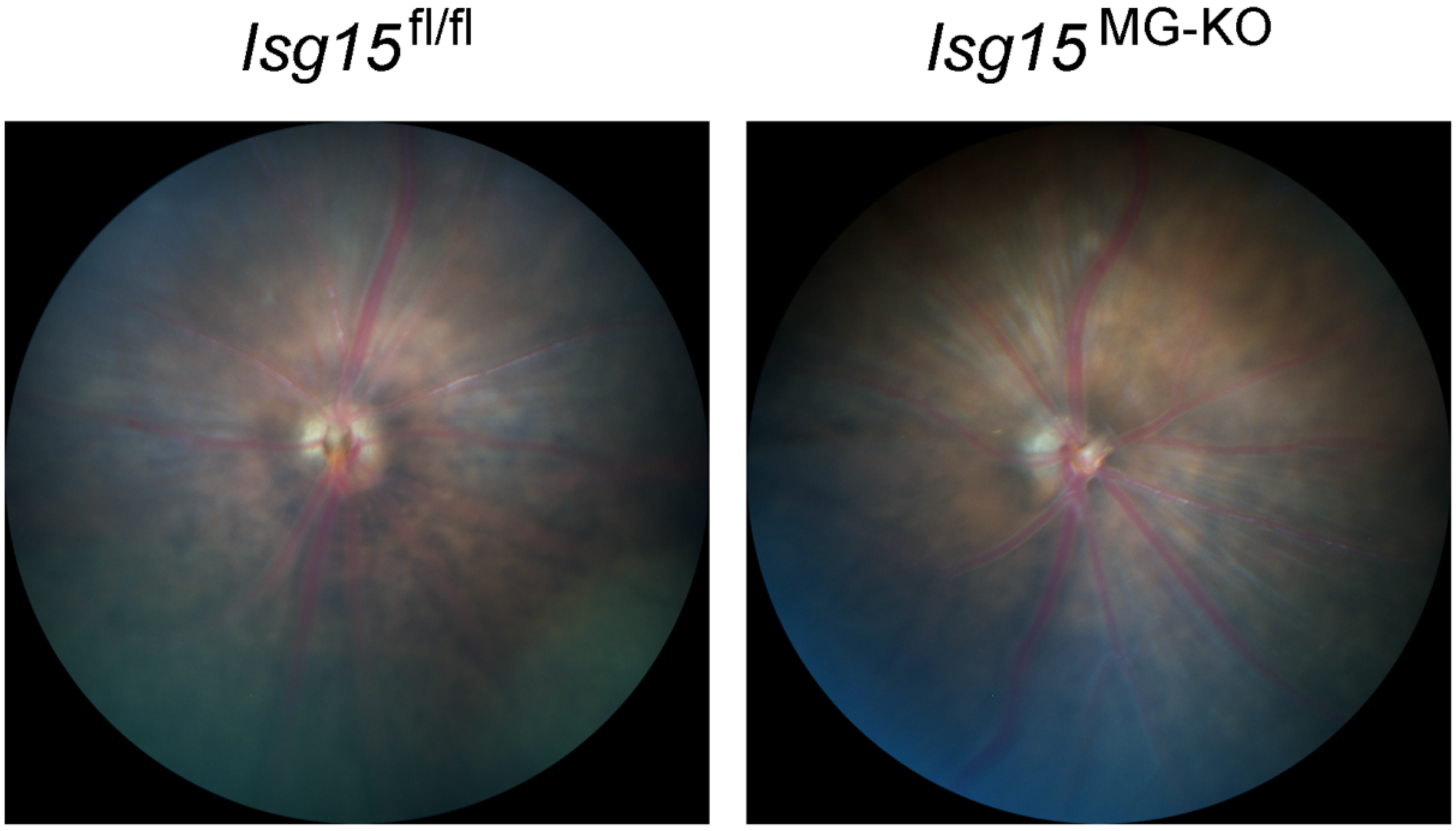

Supplemental Figure 5. Representative baseline fundus images of *Isg15*$^{fl/fl}$ and *Isg15*$^{MG\text{-}KO}$ mice. Fundus photographs were obtained from 8-week-old mice before STZ administration. Representative images from *Isg15*$^{fl/fl}$ and *Isg15*$^{MG\text{-}KO}$ mice showed no obvious gross abnormalities in fundus appearance or major retinal vascular pattern at baseline. Images are representative of n = 6 mice per genotype.

Abbreviations: STZ, streptozotocin.

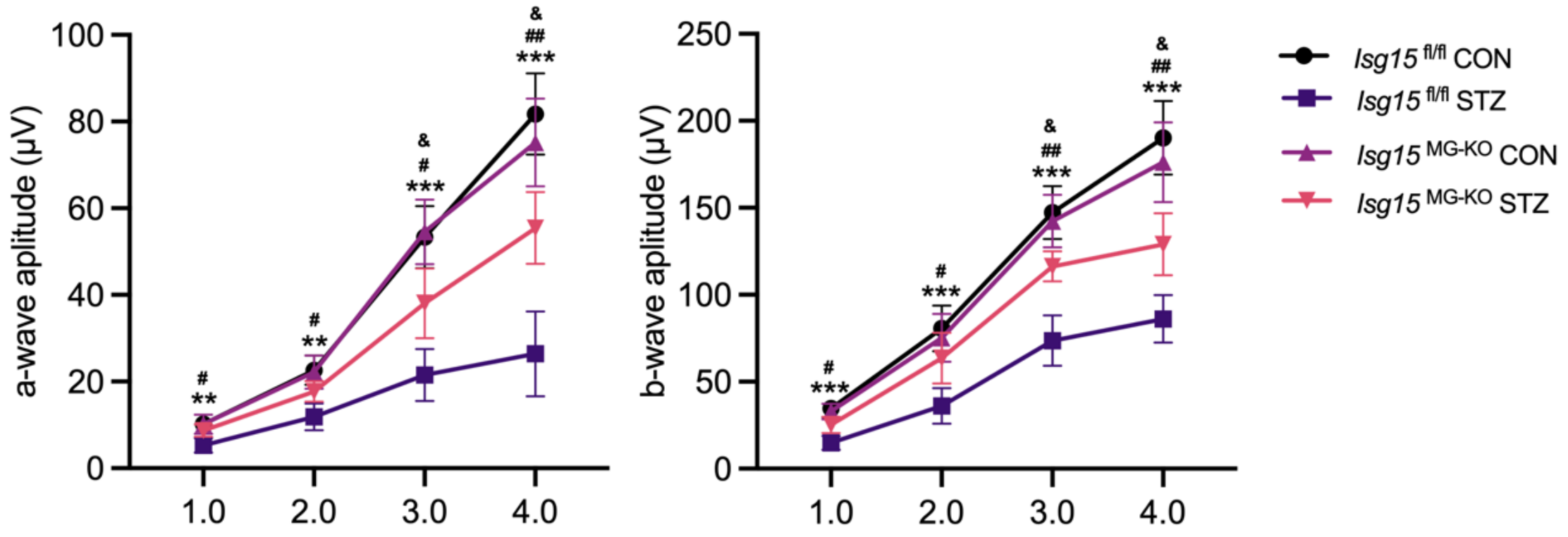

Supplemental Figure 6. Scotopic ERG analysis in *Isg15*$^{fl/fl}$ and *Isg15*$^{MG\text{-}KO}$ mice 6 months after STZ induction. Quantification of scotopic a-wave (left) and b-wave (right) amplitudes across stimulus intensities (1.0–4.0 $\log_{10}$ cd·s/m²) is shown for the indicated groups (n = 6).

Statistics: Data are mean ± SD. *P* values were determined by 2-way repeated-measures ANOVA with Šídák's multiple-comparisons test. * indicates *Isg15*$^{fl/fl}$ CON vs *Isg15*$^{fl/fl}$ STZ, # indicates *Isg15*$^{fl/fl}$ STZ vs *Isg15*$^{MG\text{-}KO}$ STZ, and & indicates *Isg15*$^{MG\text{-}KO}$ CON vs *Isg15*$^{MG\text{-}KO}$ STZ. Single, double, and triple symbols indicate $P < 0.05$, $P < 0.01$, and $P < 0.001$, respectively. Only significant comparisons are annotated.

Abbreviations: CON, control; STZ, streptozotocin; ERG, electroretinography.

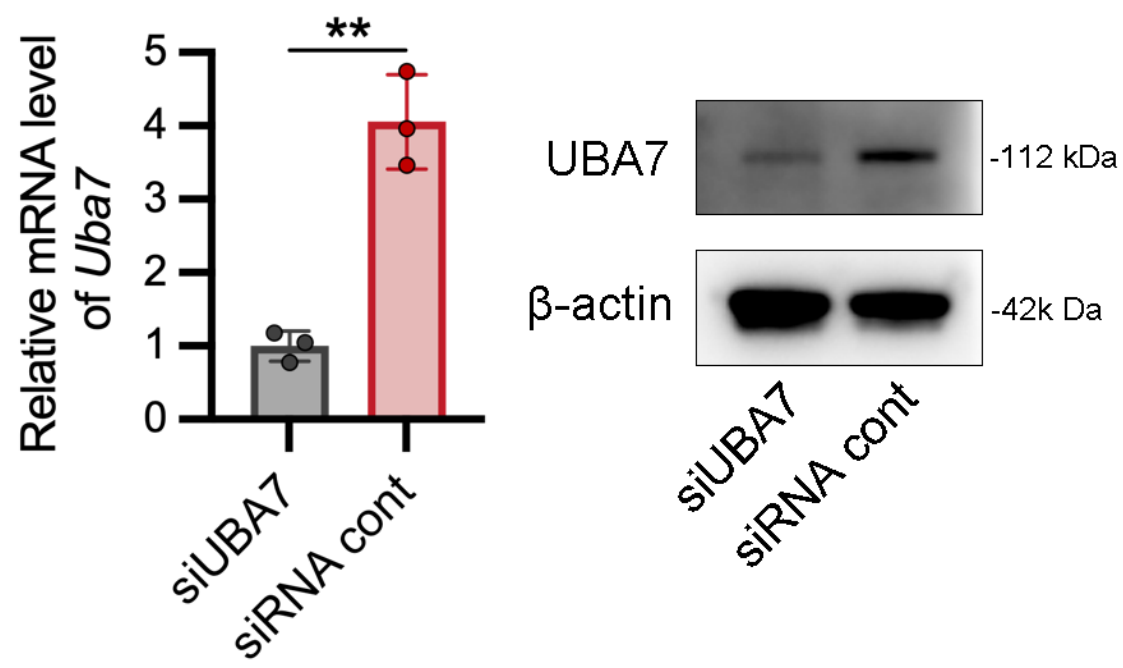

Supplemental Figure 7. Validation of UBA7 knockdown efficiency in primary Müller glia.

Primary Müller glia were transfected with UBA7 siRNA or control siRNA. RT-qPCR analysis of *Uba7* mRNA levels is shown on the left (n = 3), and representative immunoblot analysis of UBA7 protein abundance, with β-actin as the loading control, is shown on the right.

Immunoblots are representative of 3 independent experiments.

Statistics: Data are mean ± SD. *P* values were determined by 2-tailed unpaired Student's t-test. Significance as indicated (**P* < 0.05, ***P* < 0.01, ****P* < 0.001; ns, not significant).

Abbreviations: siRNA, small interfering RNA; UBA7, ubiquitin-like modifier activating enzyme 7.
