# Supplementary material for: ISG15+ Müller glial subtype contributes to early blood–retinal barrier breakdown in diabetic retinopathy
Source: J Clin Invest. 2026 Oct 15;136(20):e205800. doi: 10.1172/JCI205800 (PMC13668631; doi:10.1172/JCI205800)

Full unedited blot for Figure 2A

ISG15

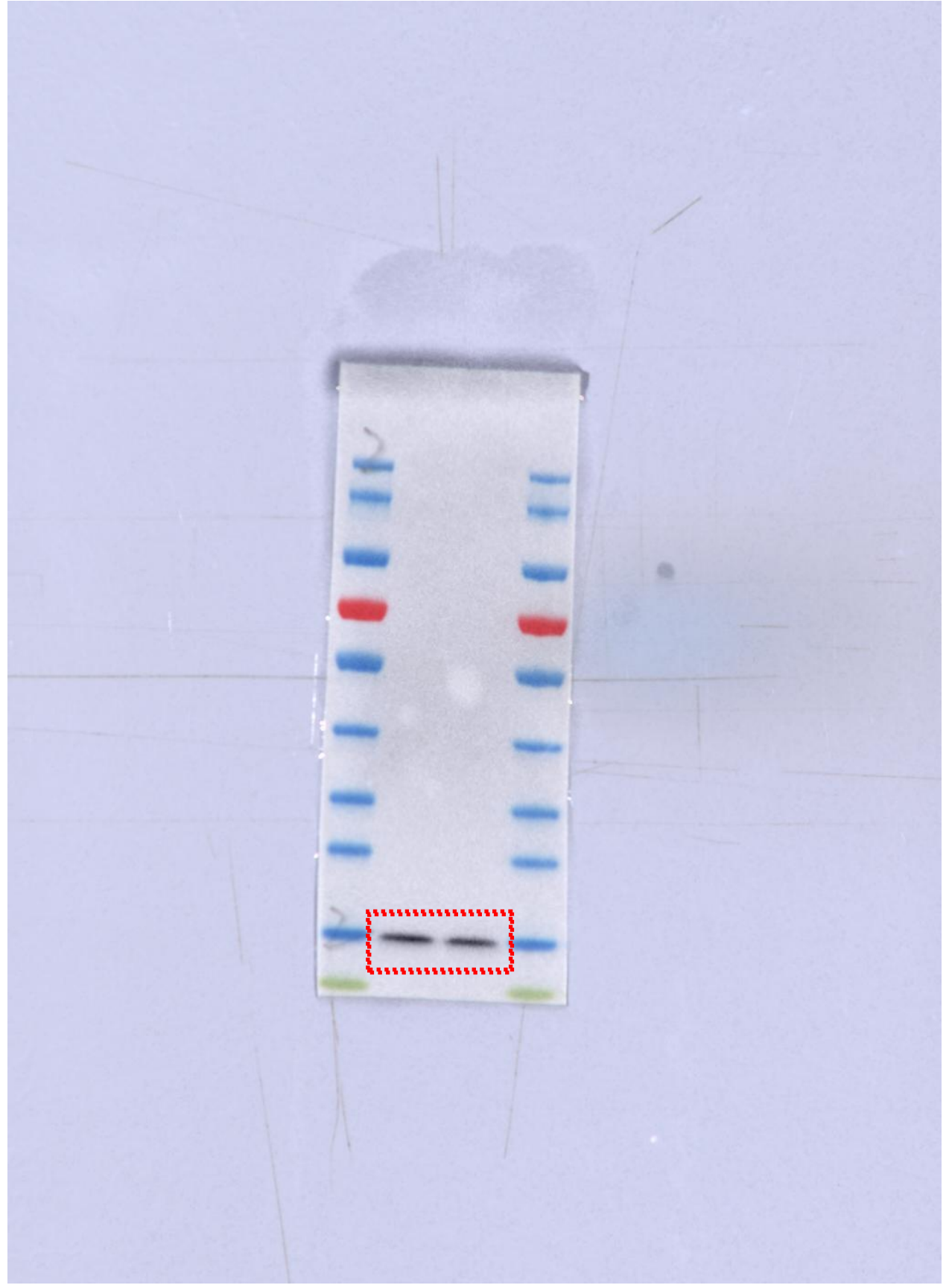

ISGylation

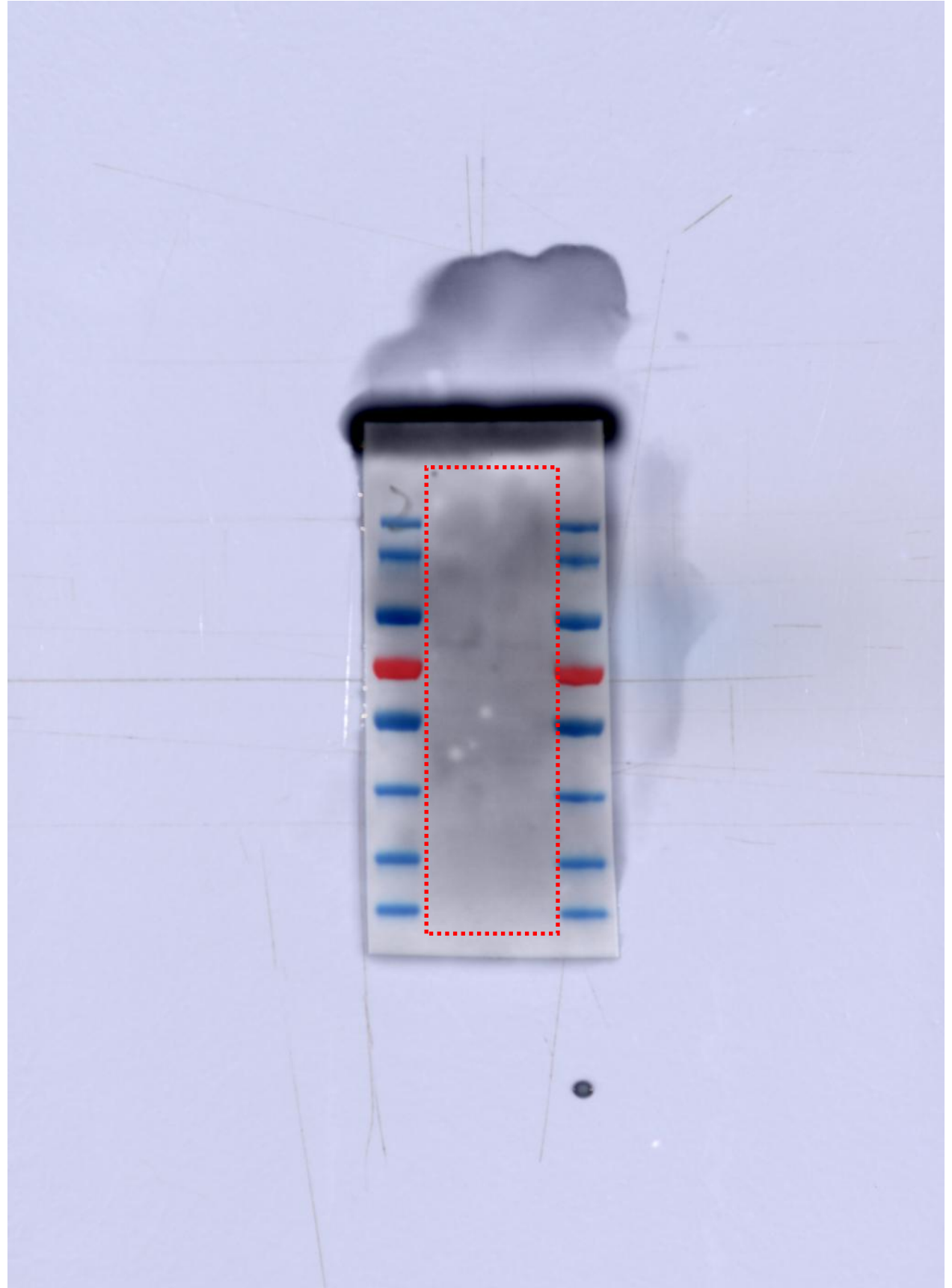

β–actin

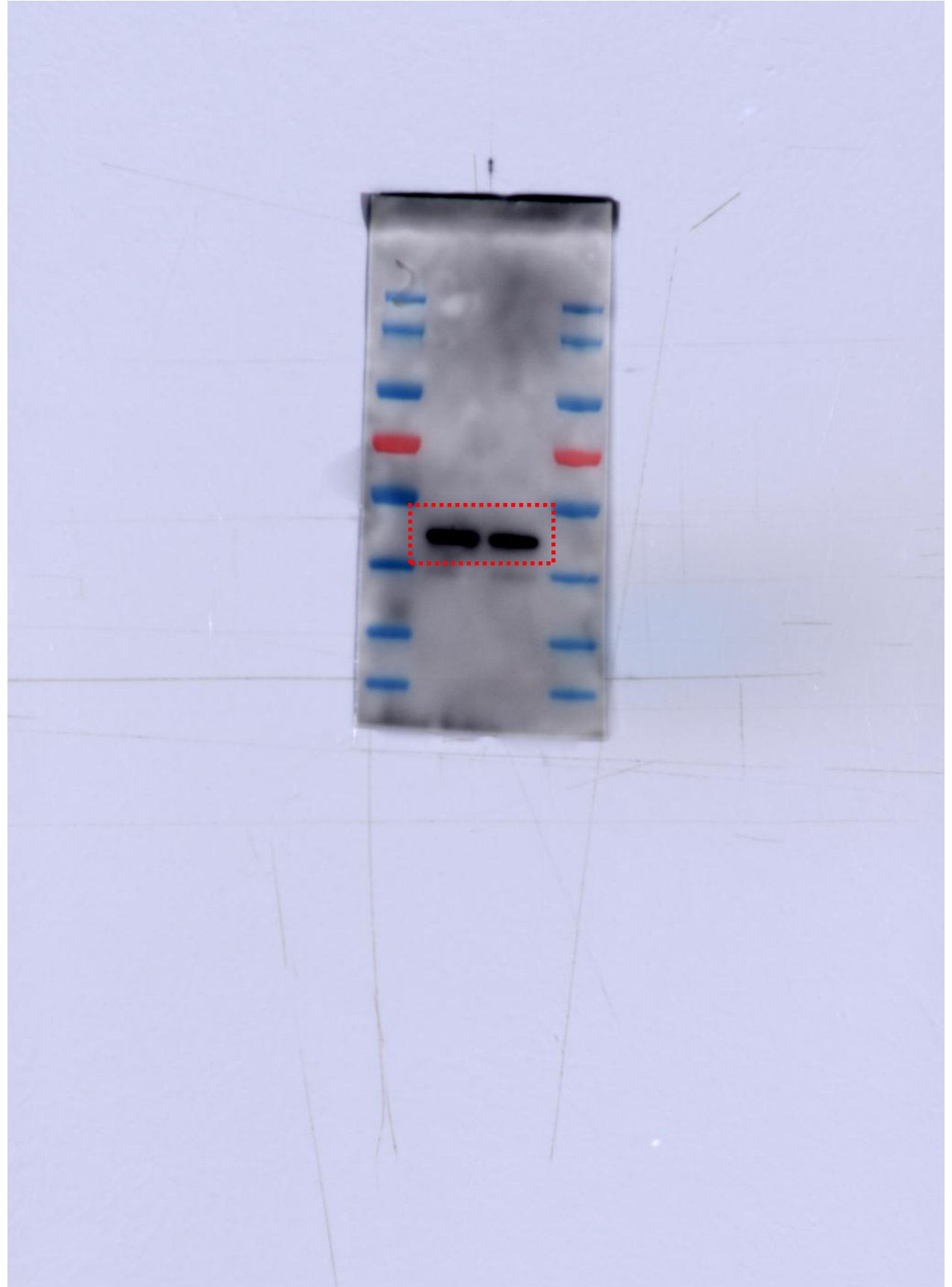

Full unedited blot for Figure 2B

ISG15

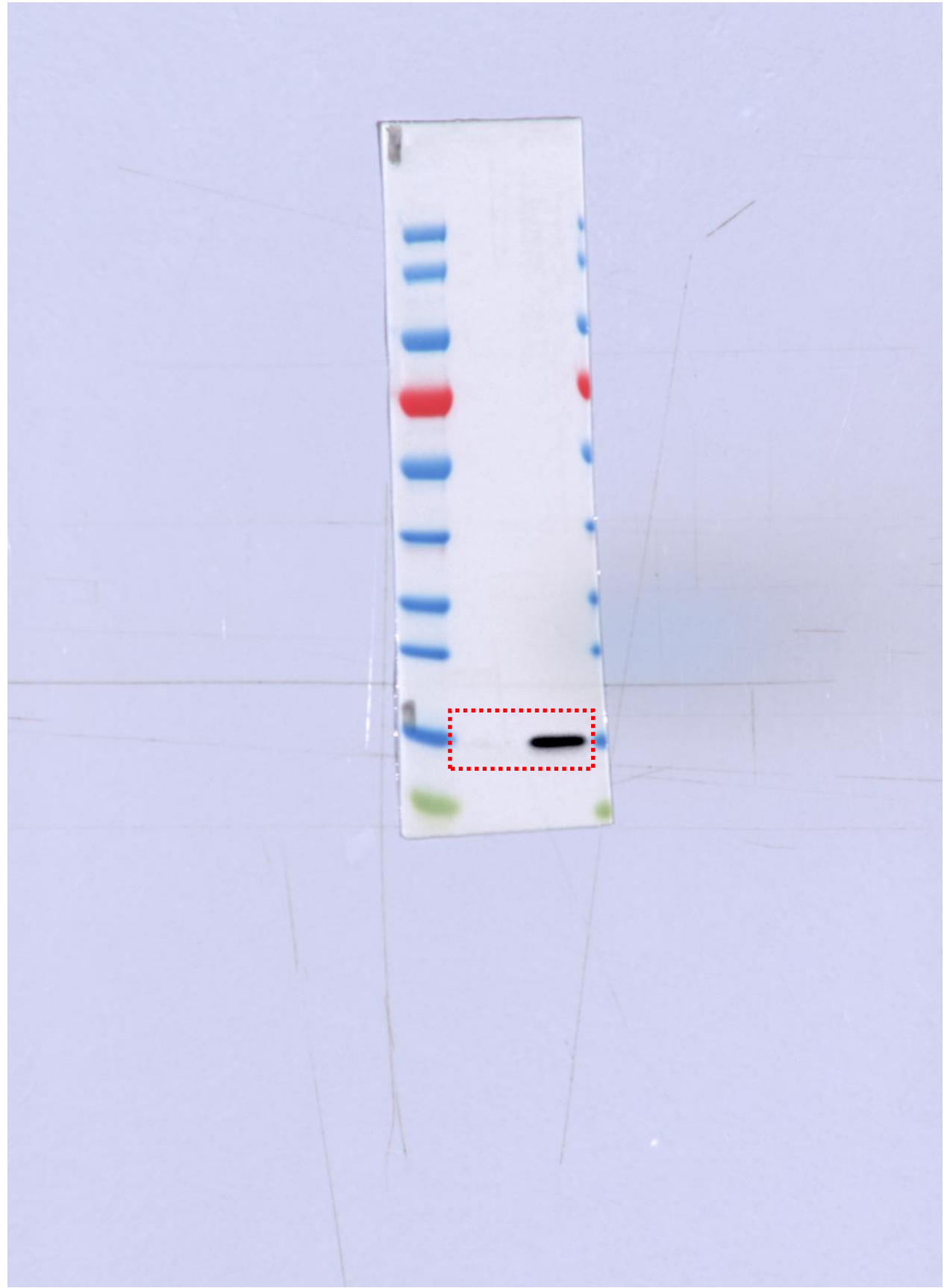

ISGylation

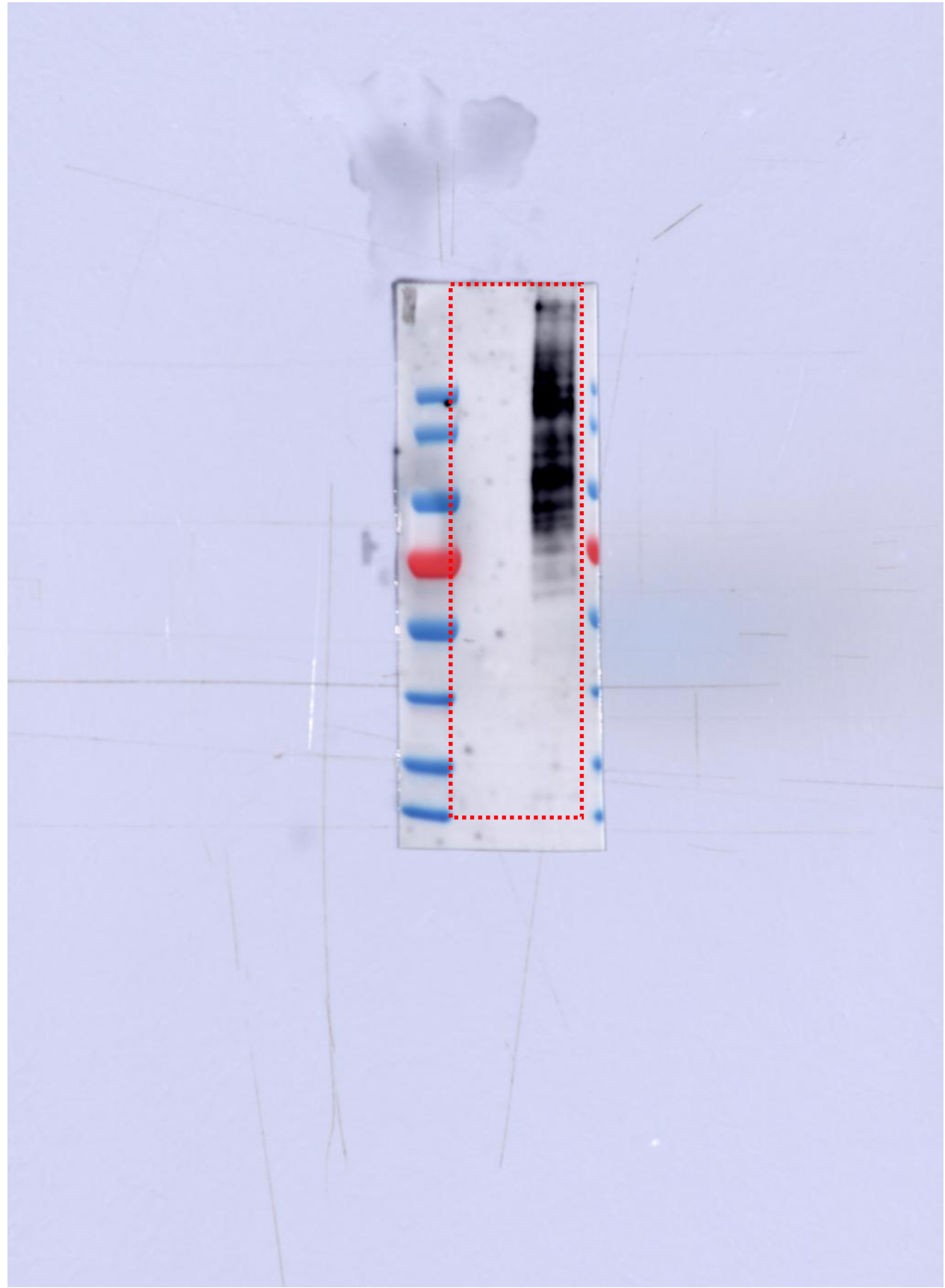

β–actin

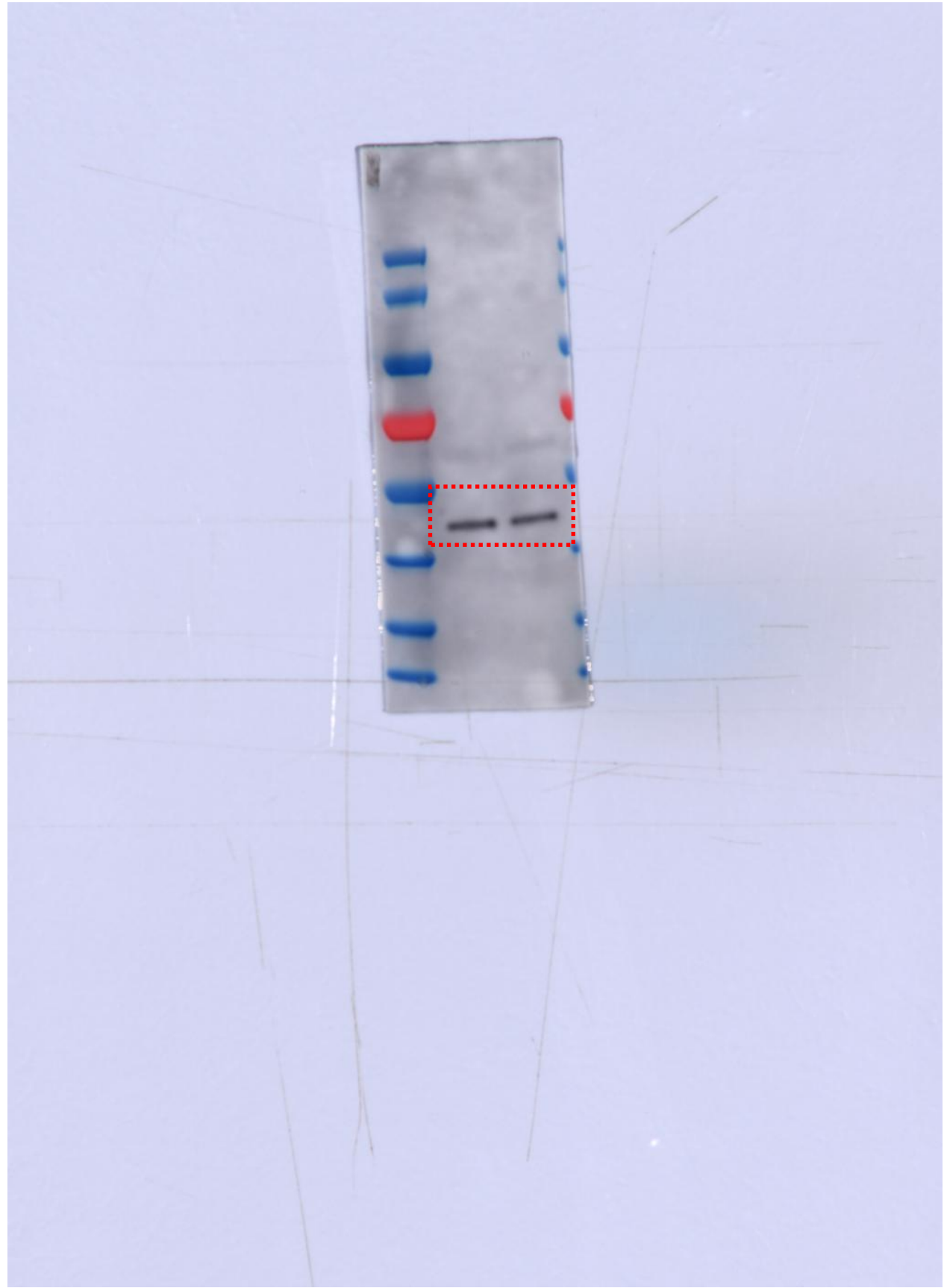

Full unedited blot for Figure 2D

ISG15

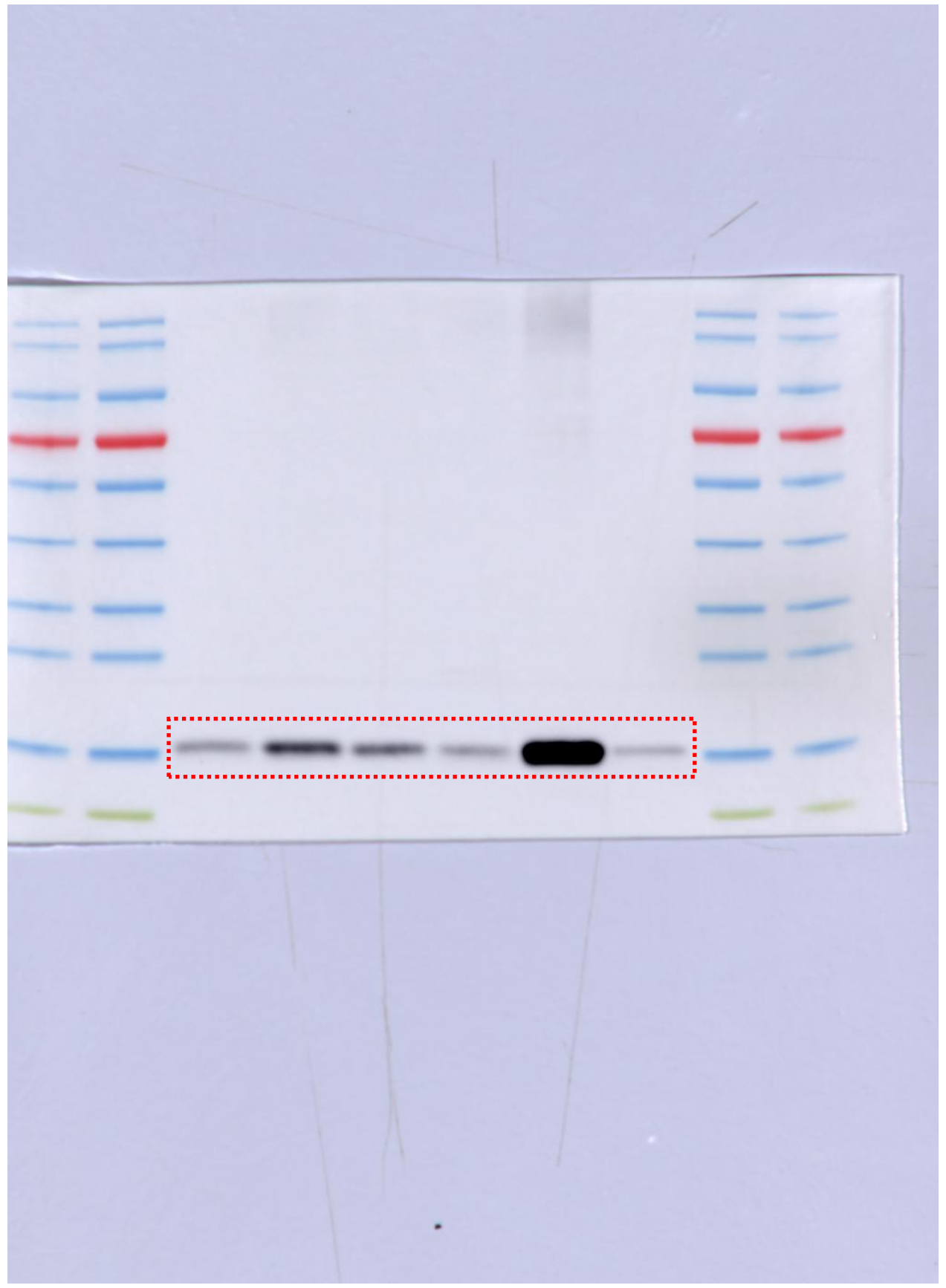

ISGylation

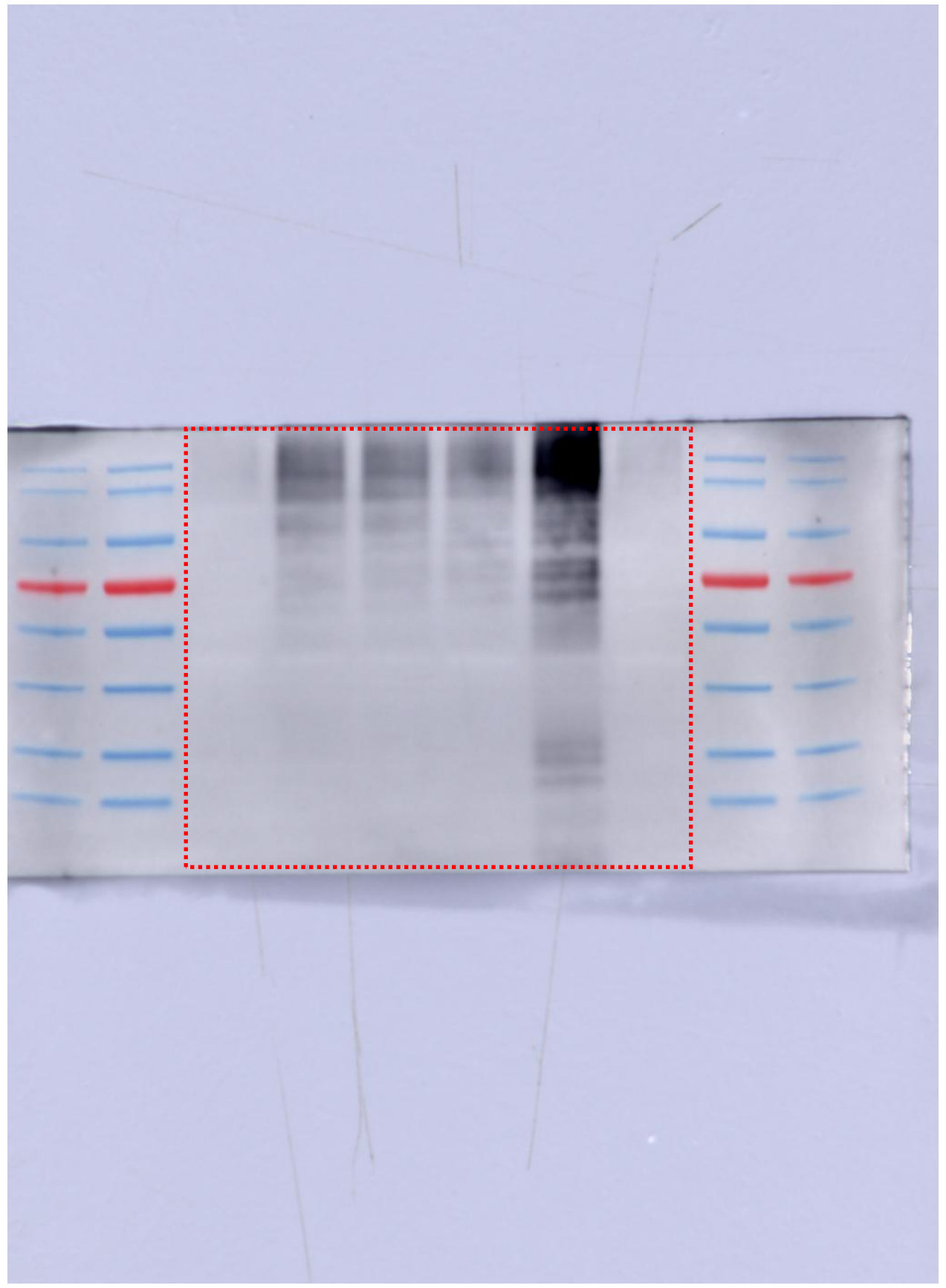

β–actin

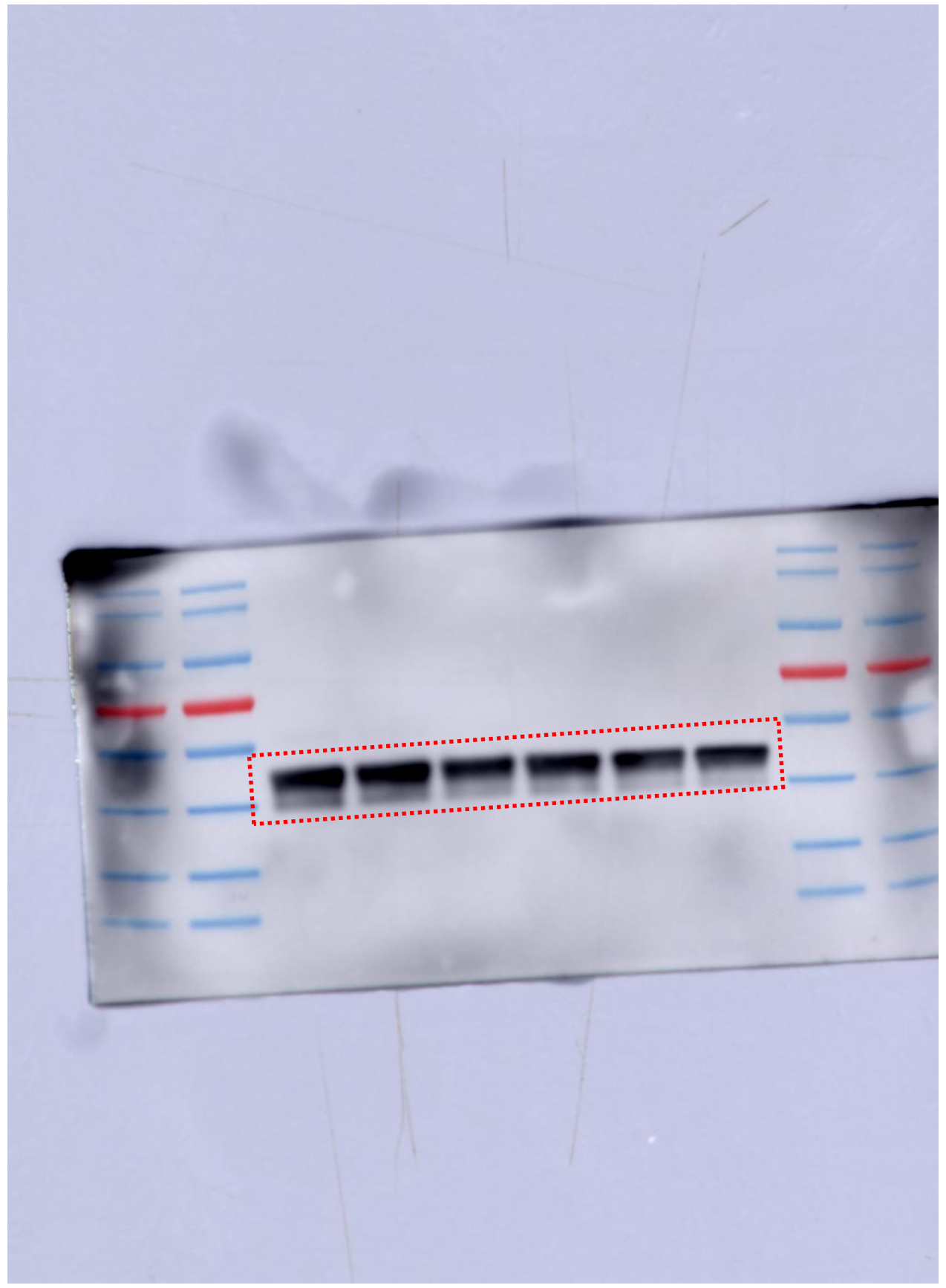

Full unedited blot for Figure 2E, F

ISG15

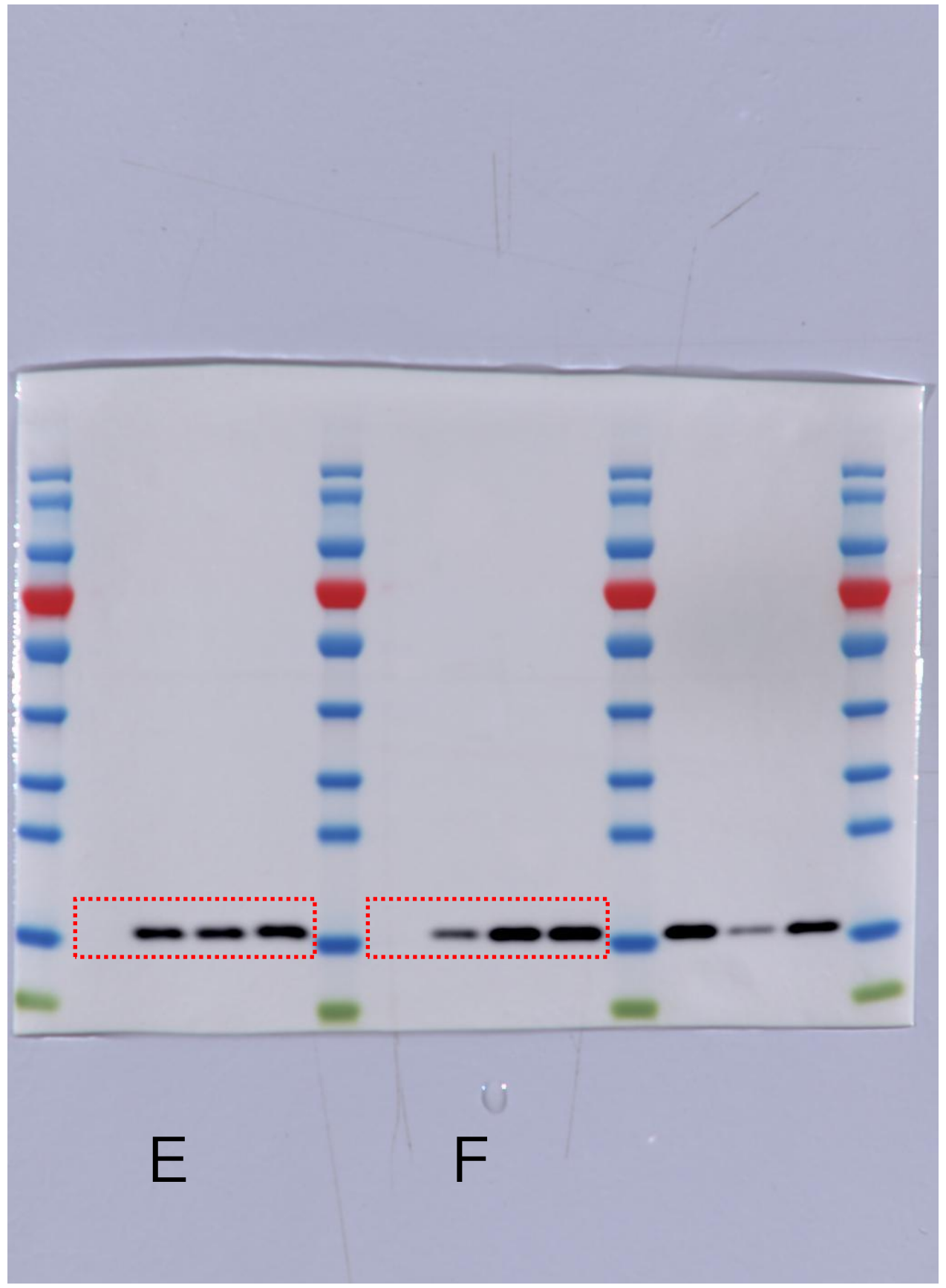

ISGylation

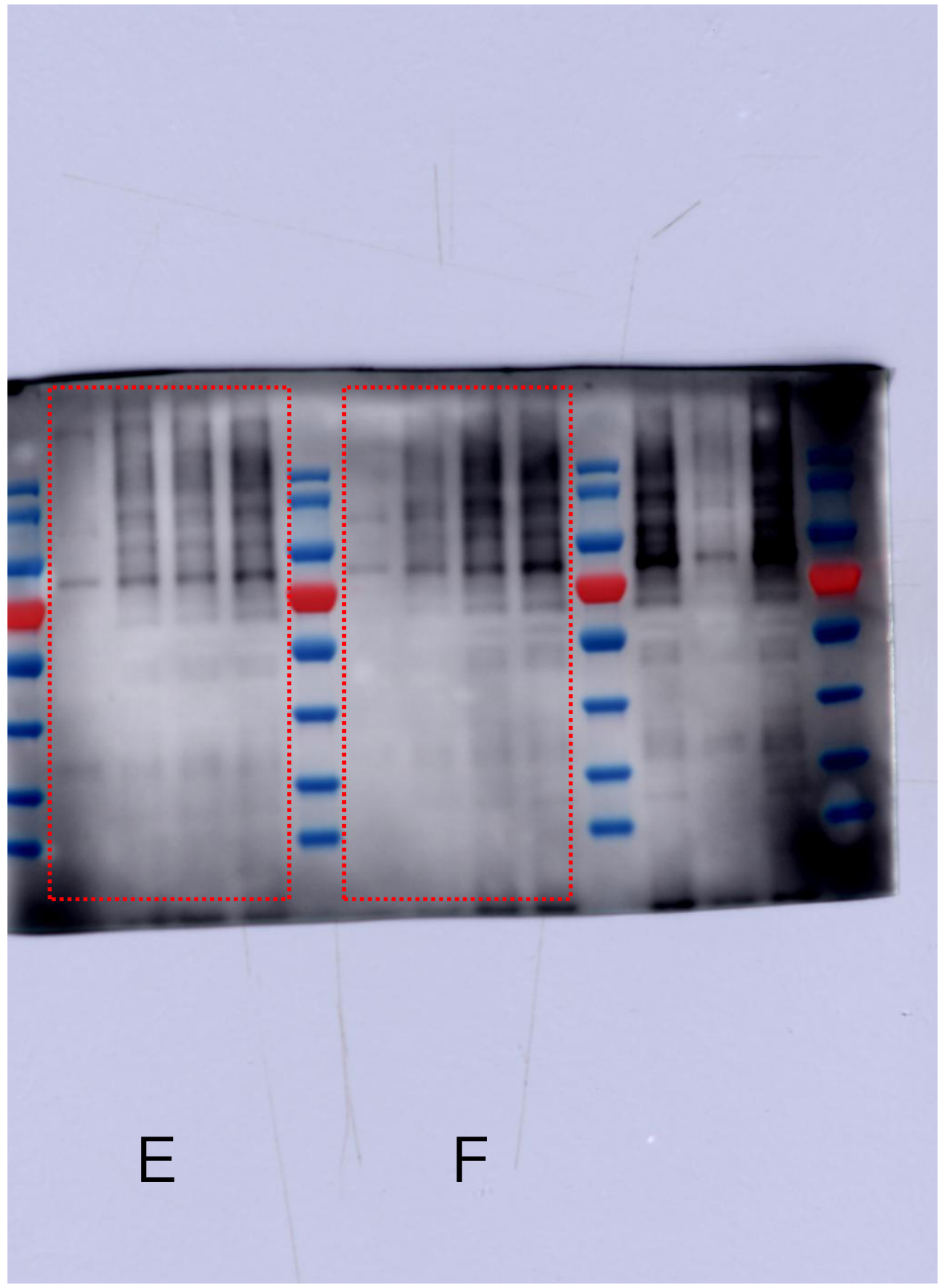

β–actin

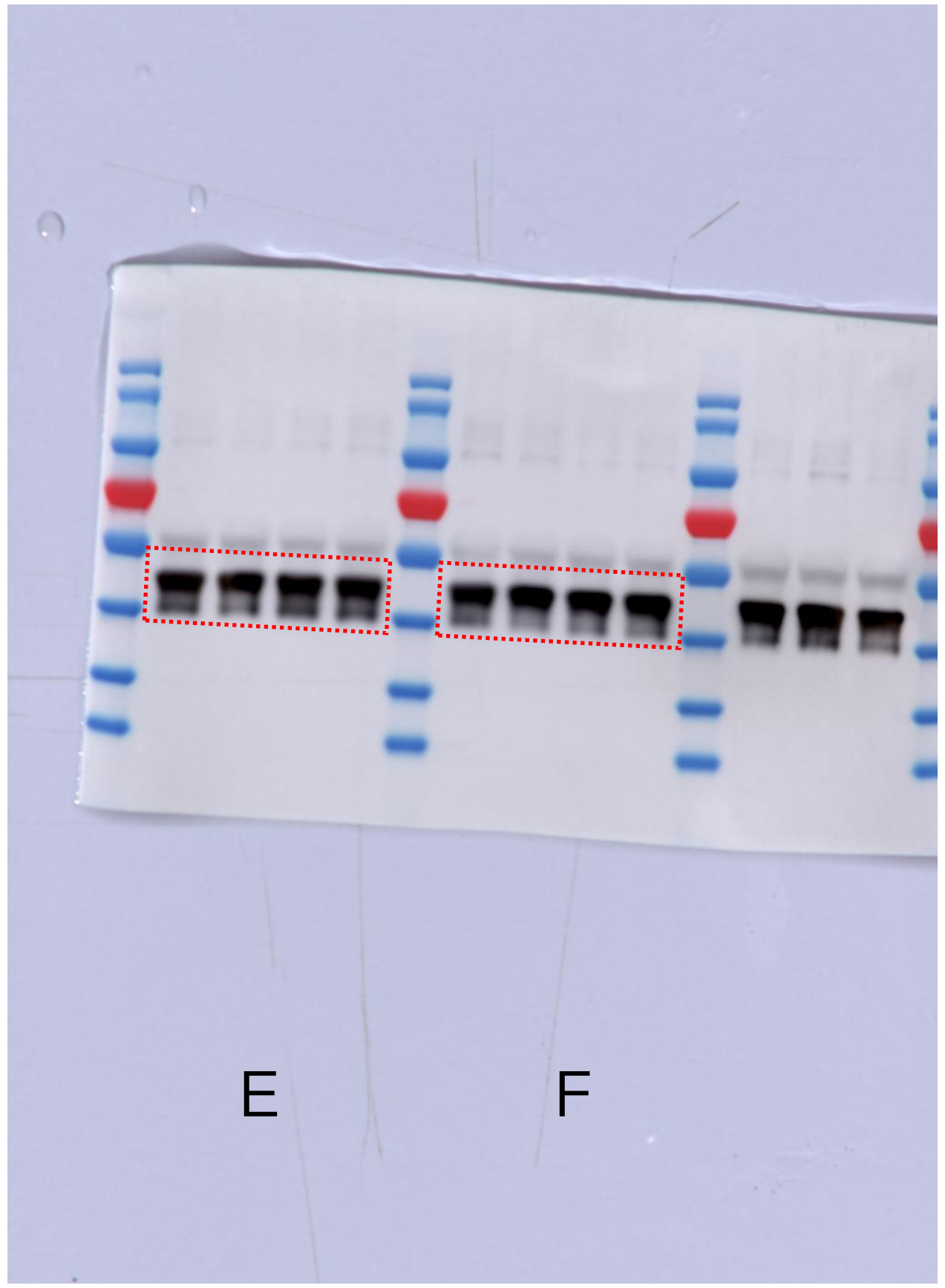

Full unedited blot for Figure 2G

ISG15

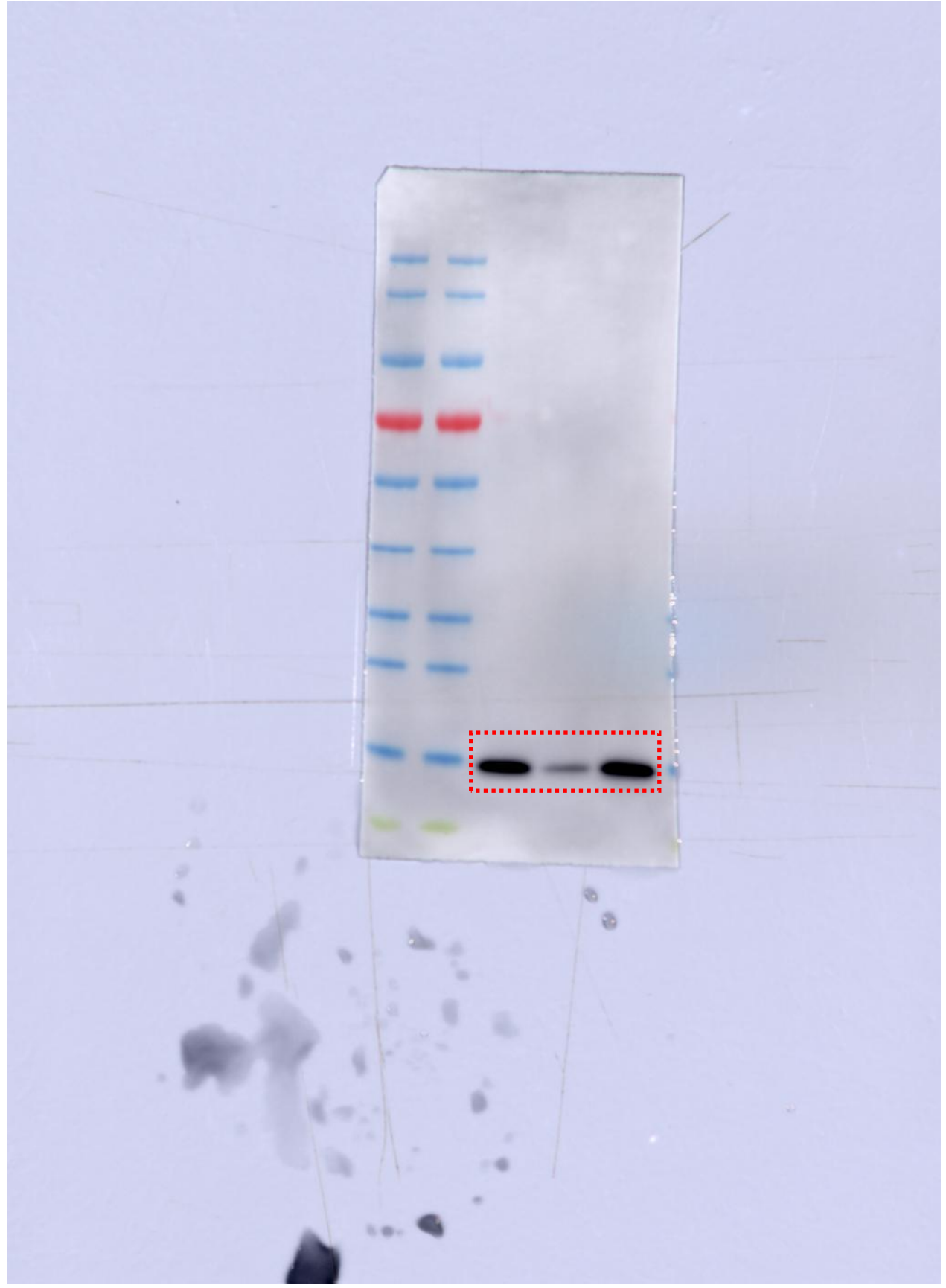

ISGylation

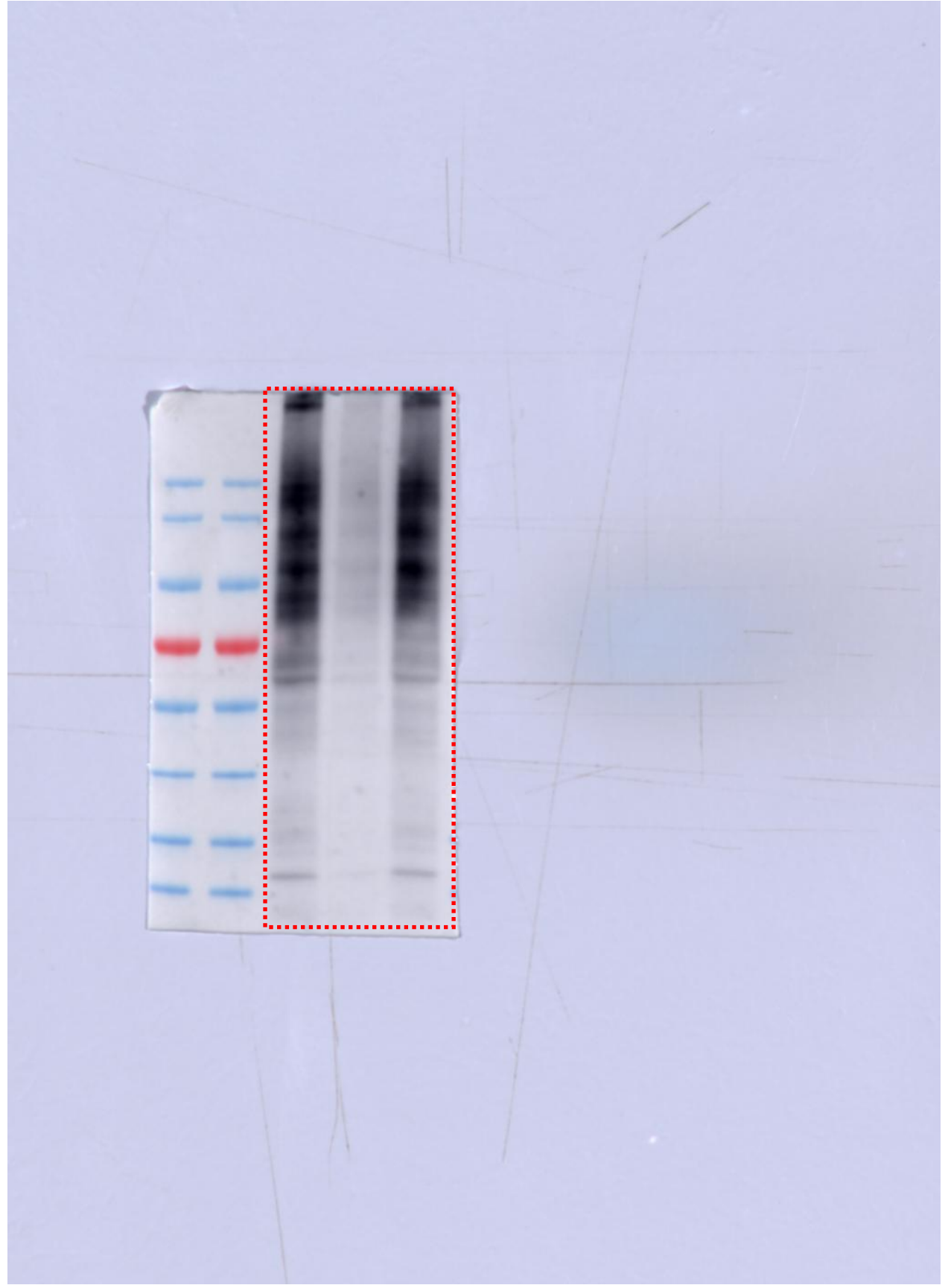

β–actin

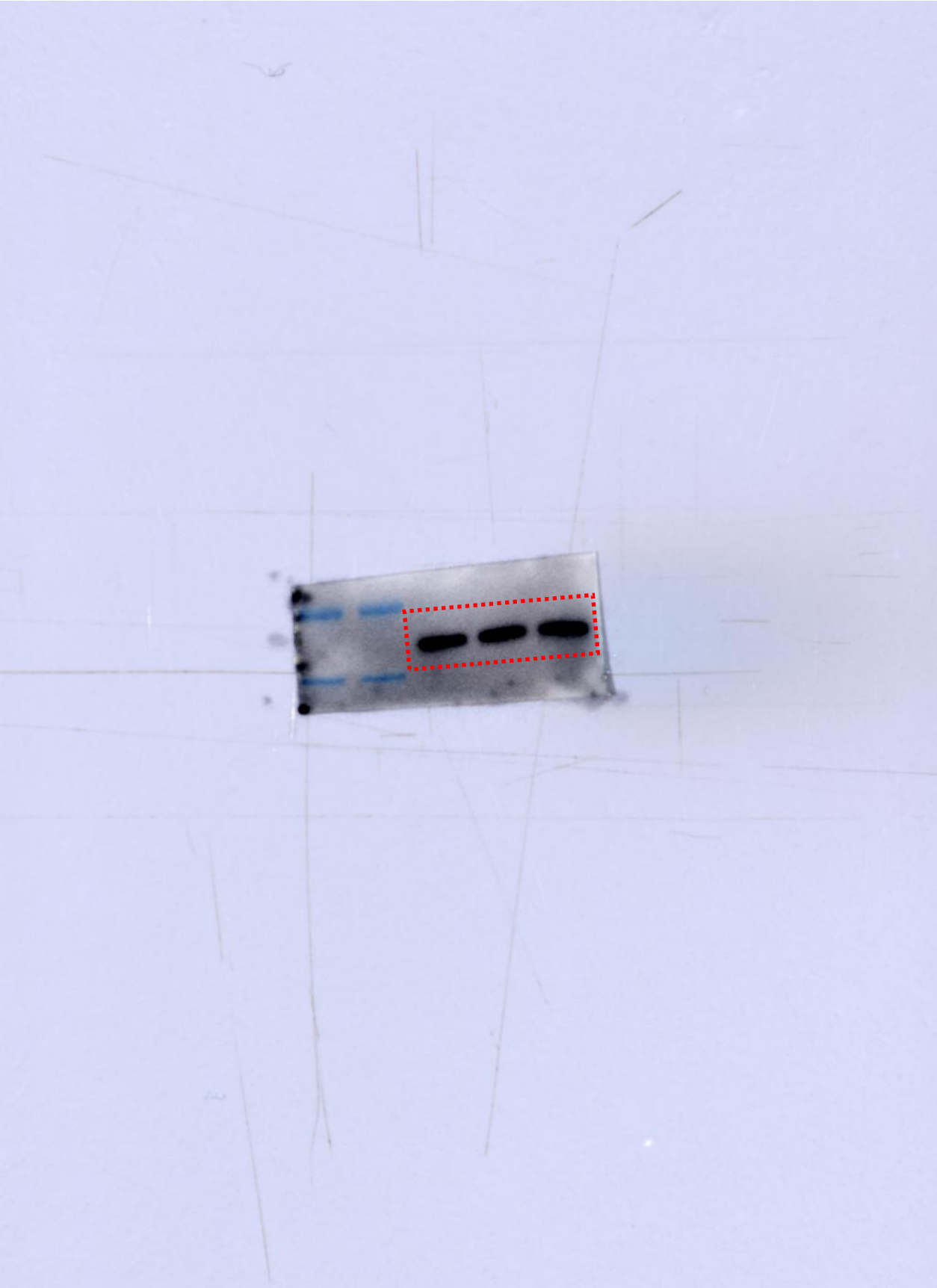

Full unedited blot for Figure 4A

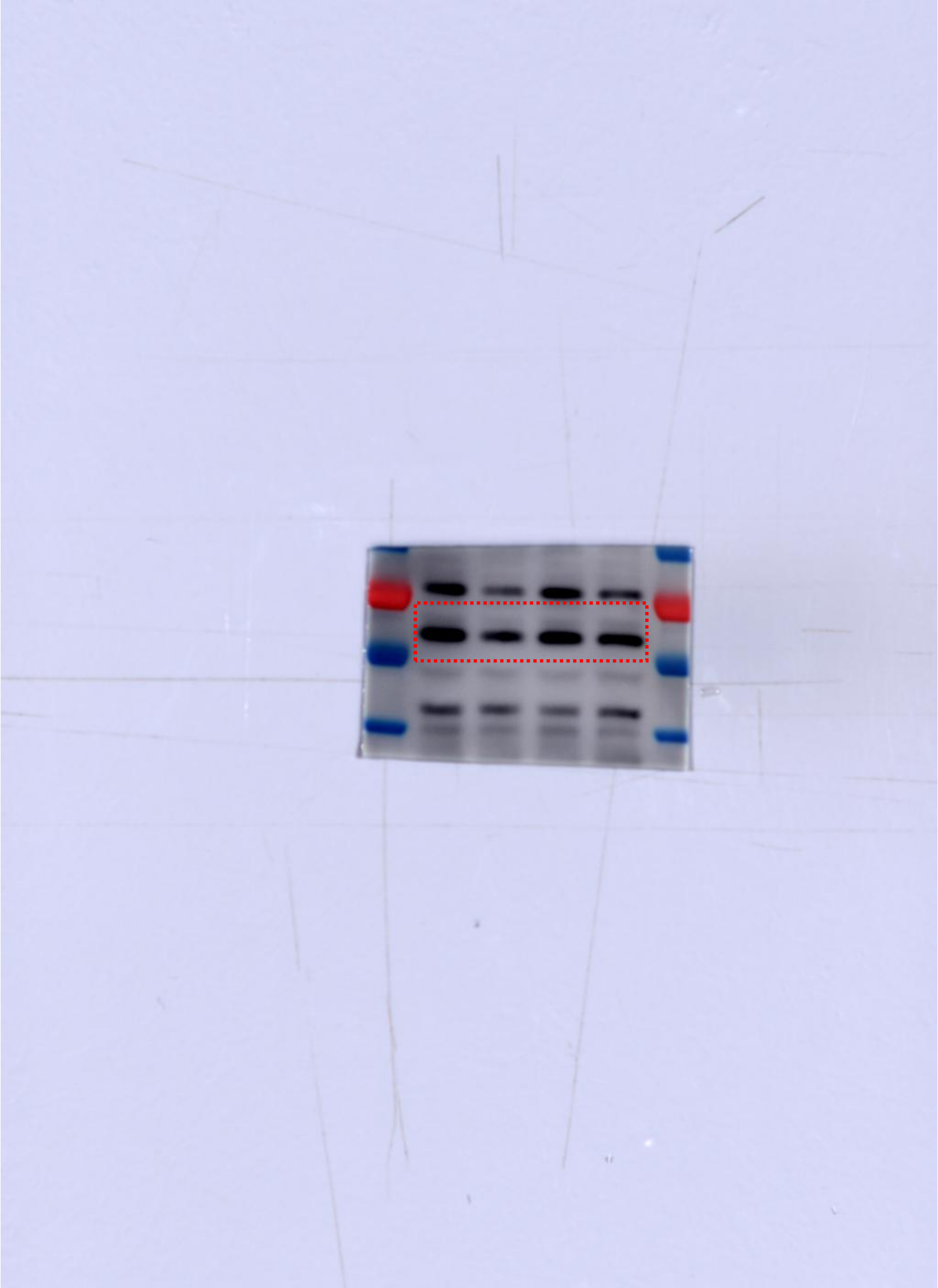

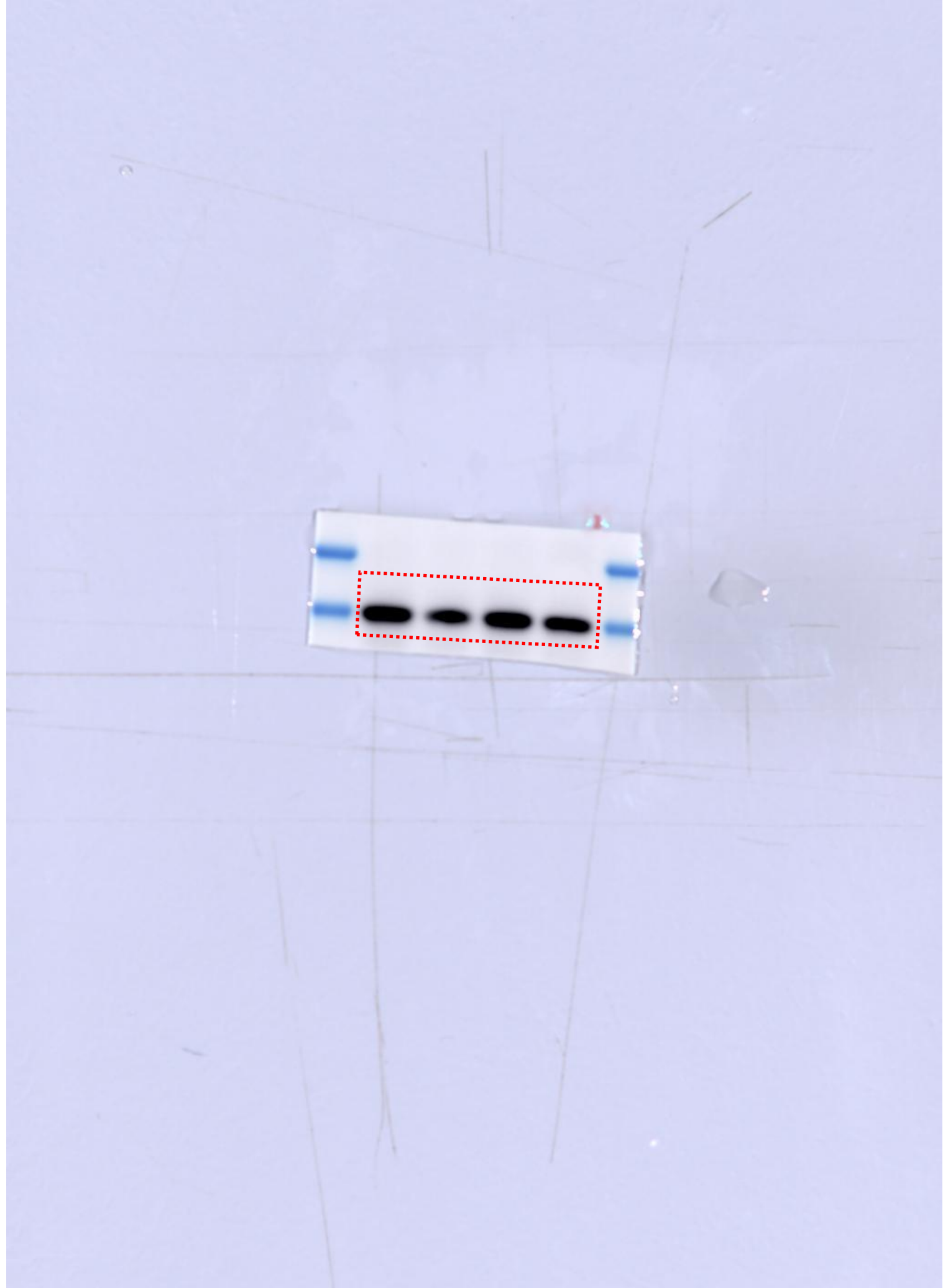

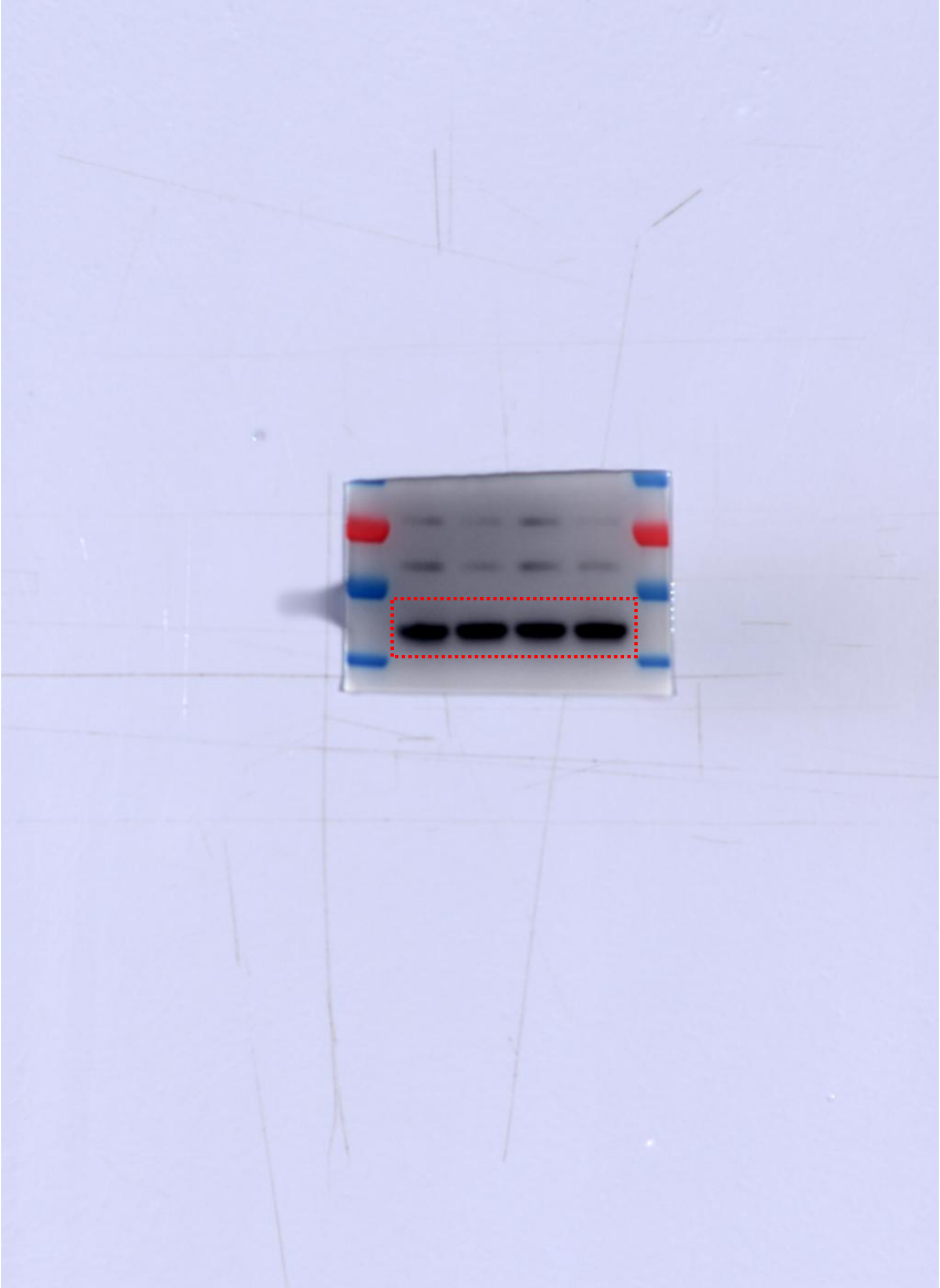

Full unedited blot for Figure 6A

4−HNE

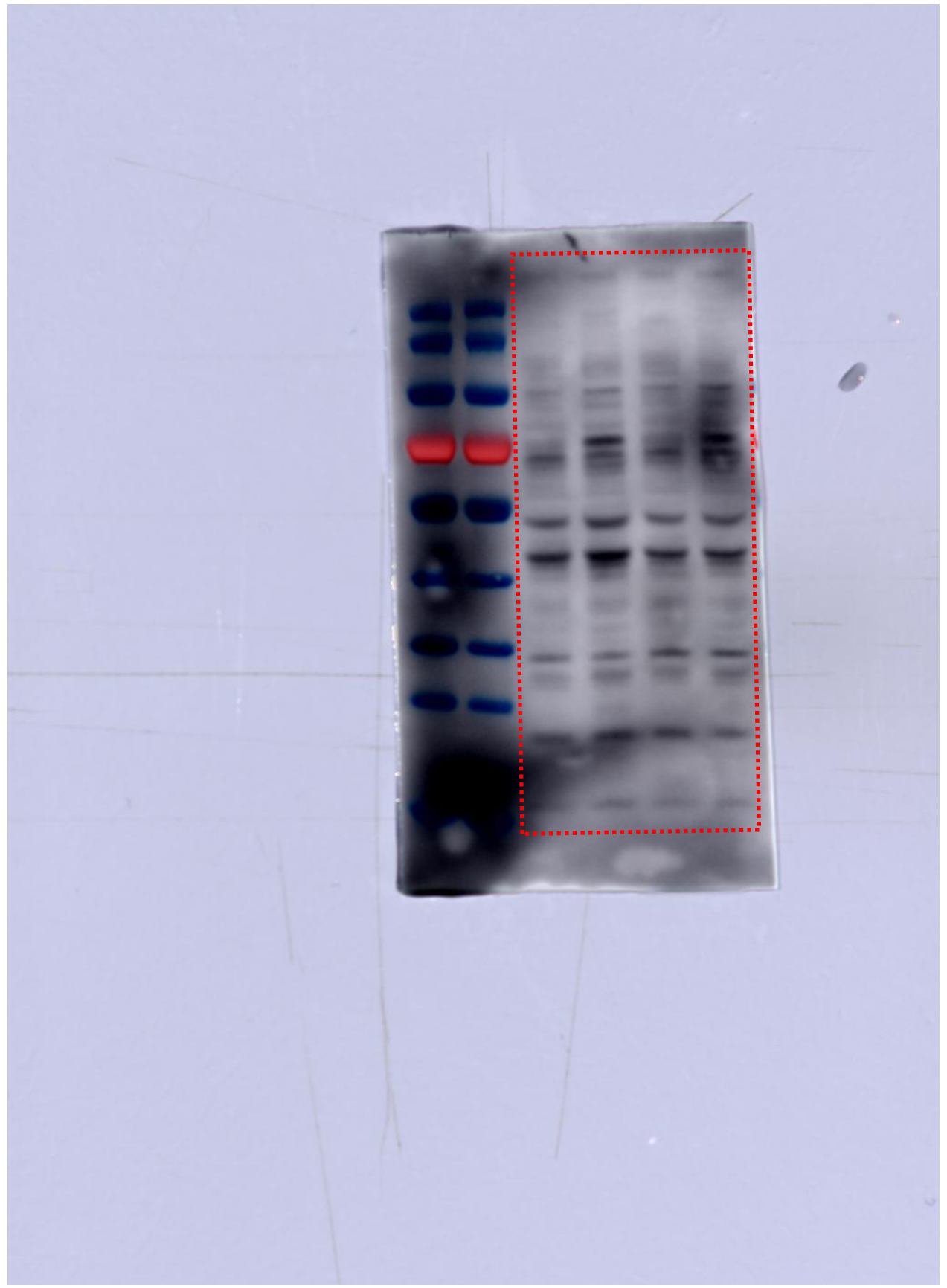

β−actin

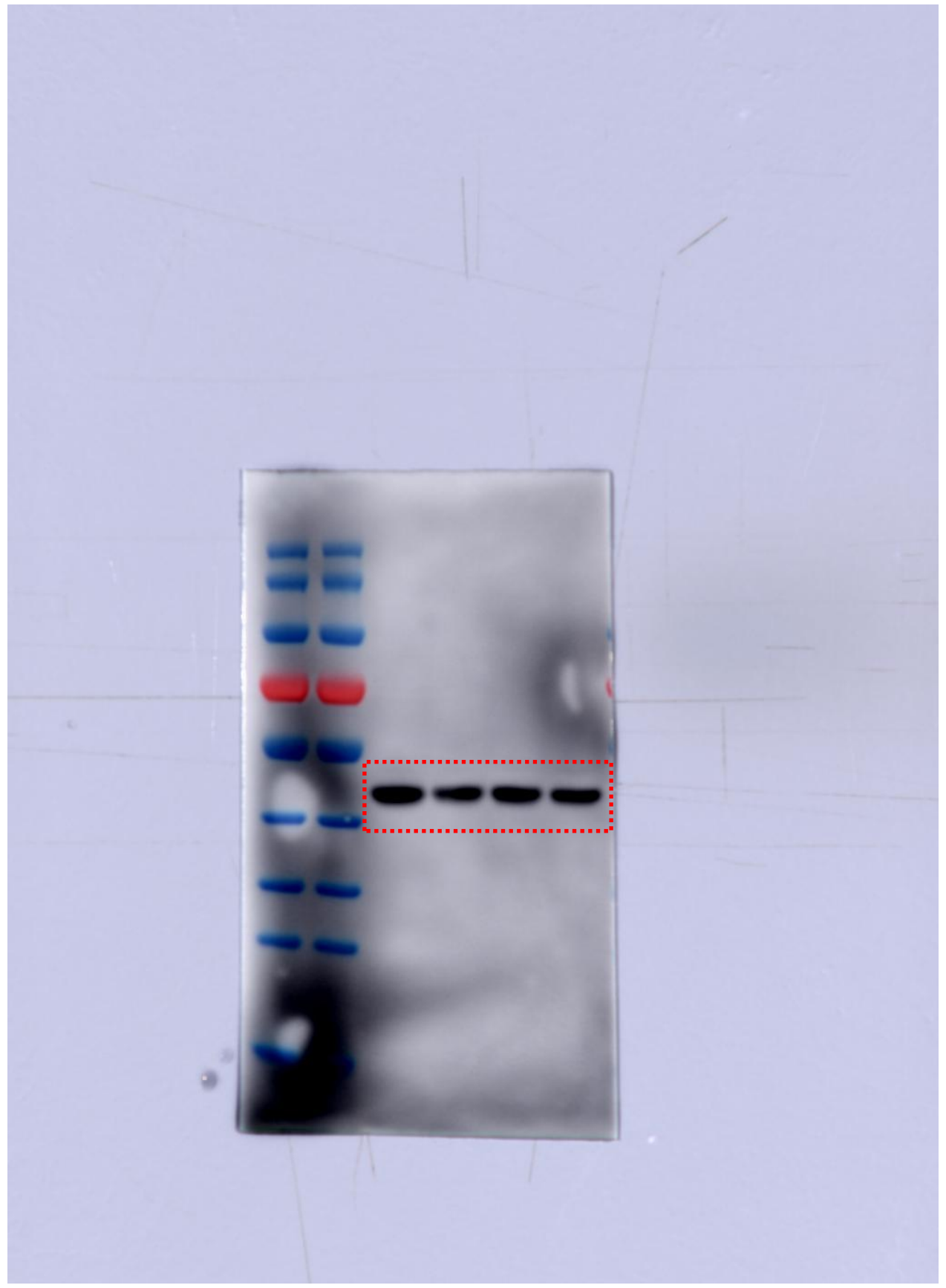

Full unedited blot for Figure 11A

PRDX6

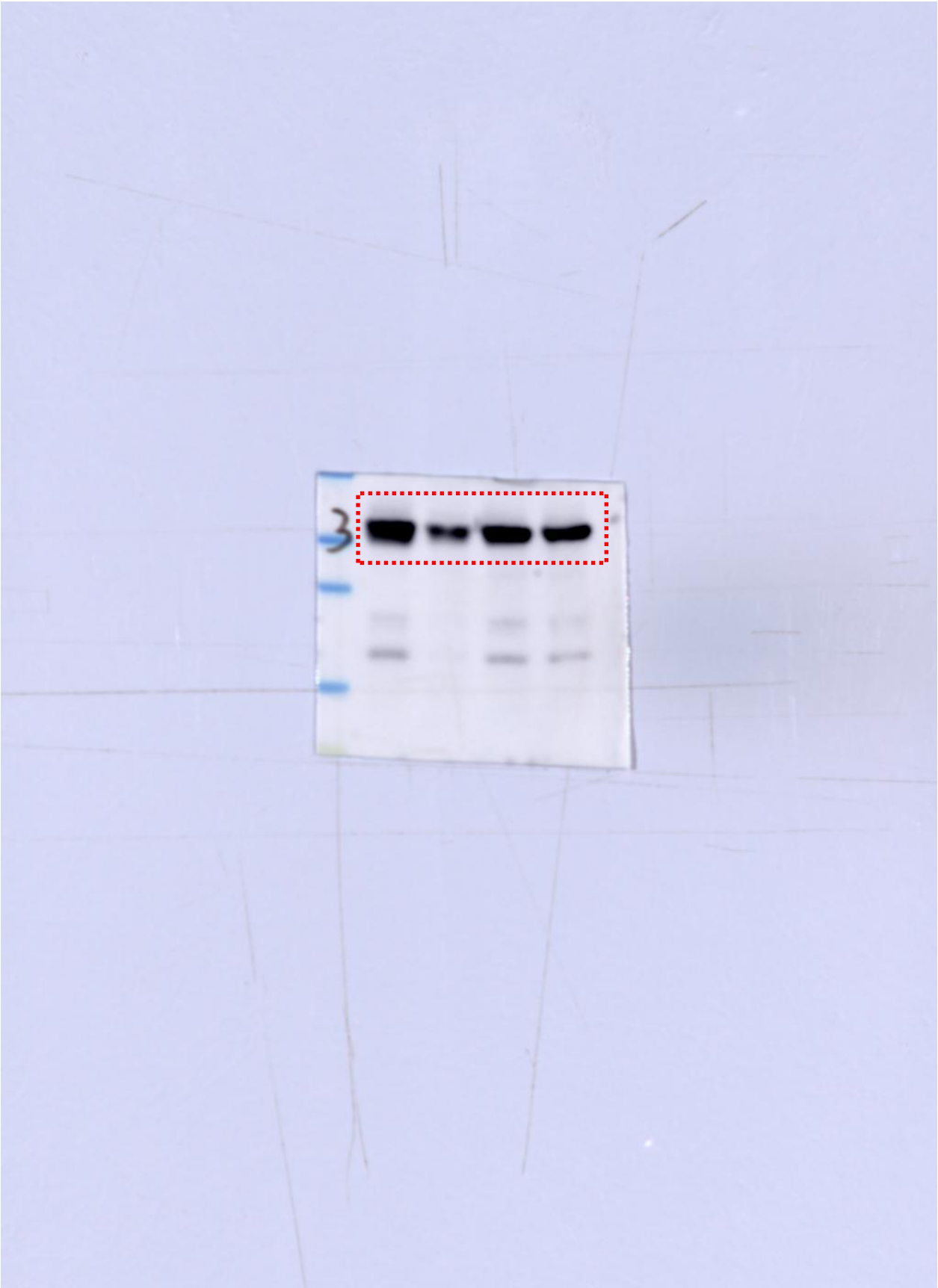

β–actin

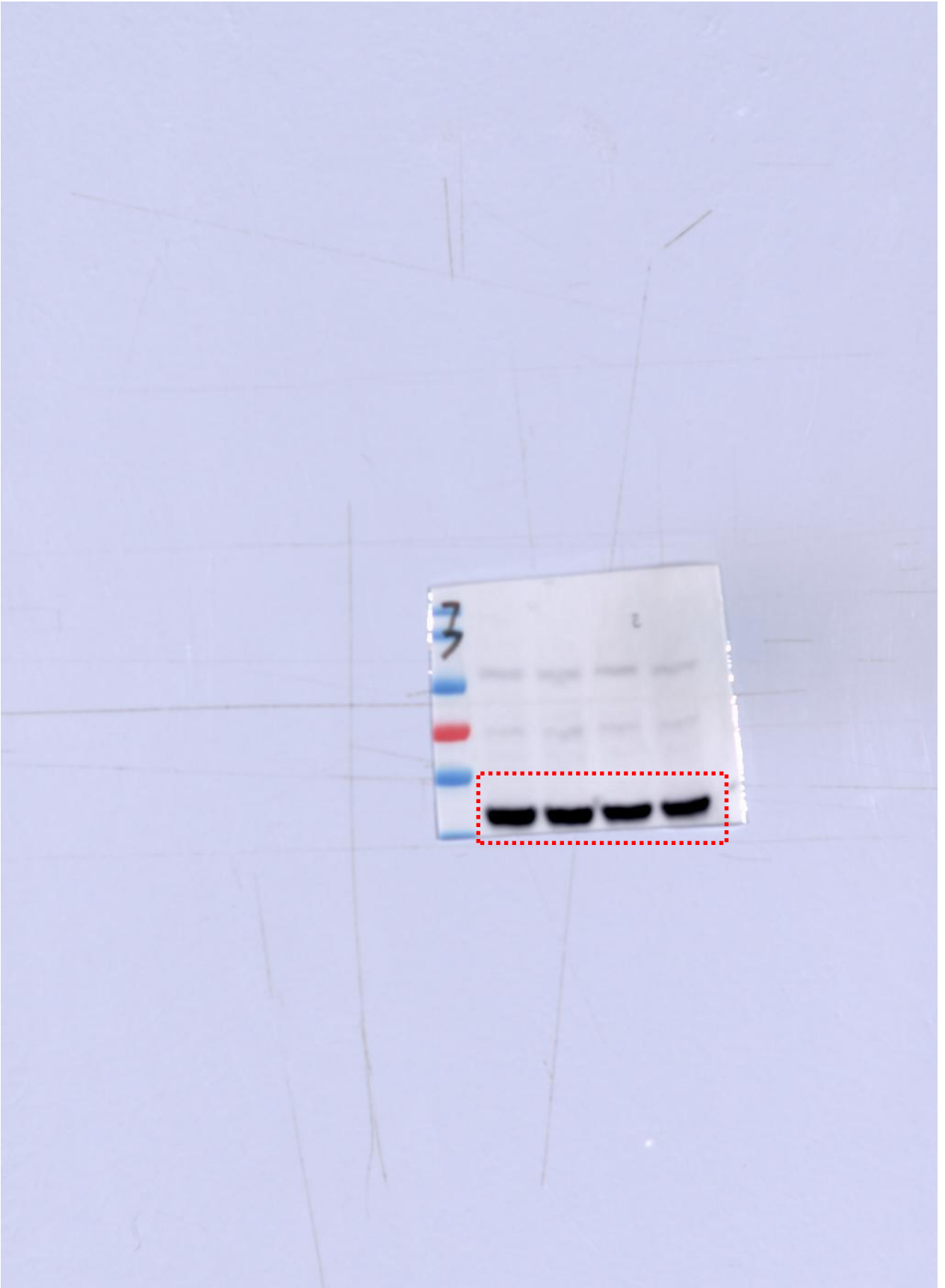

Full unedited blot for Figure 11B

PRDX6

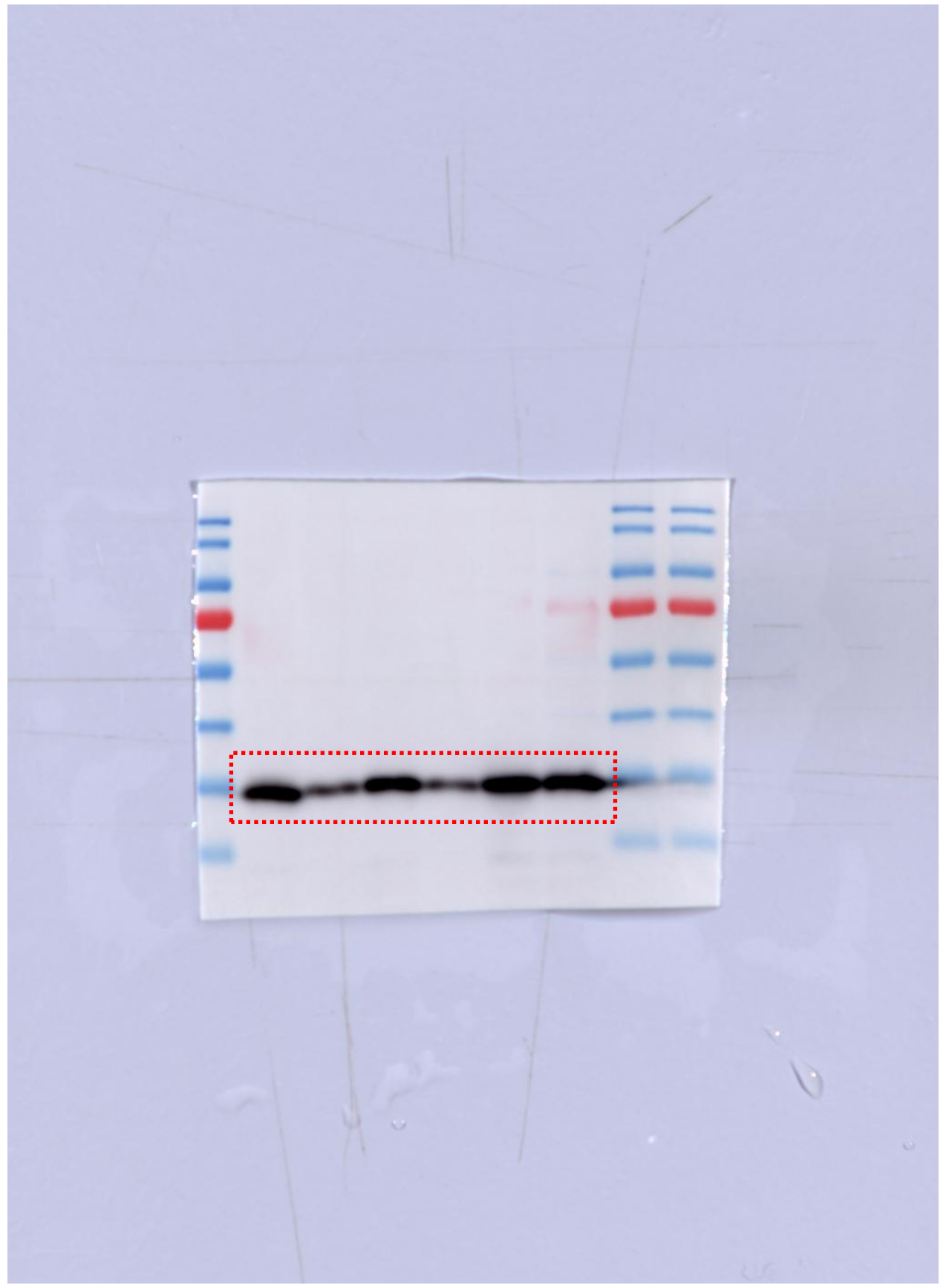

β–actin

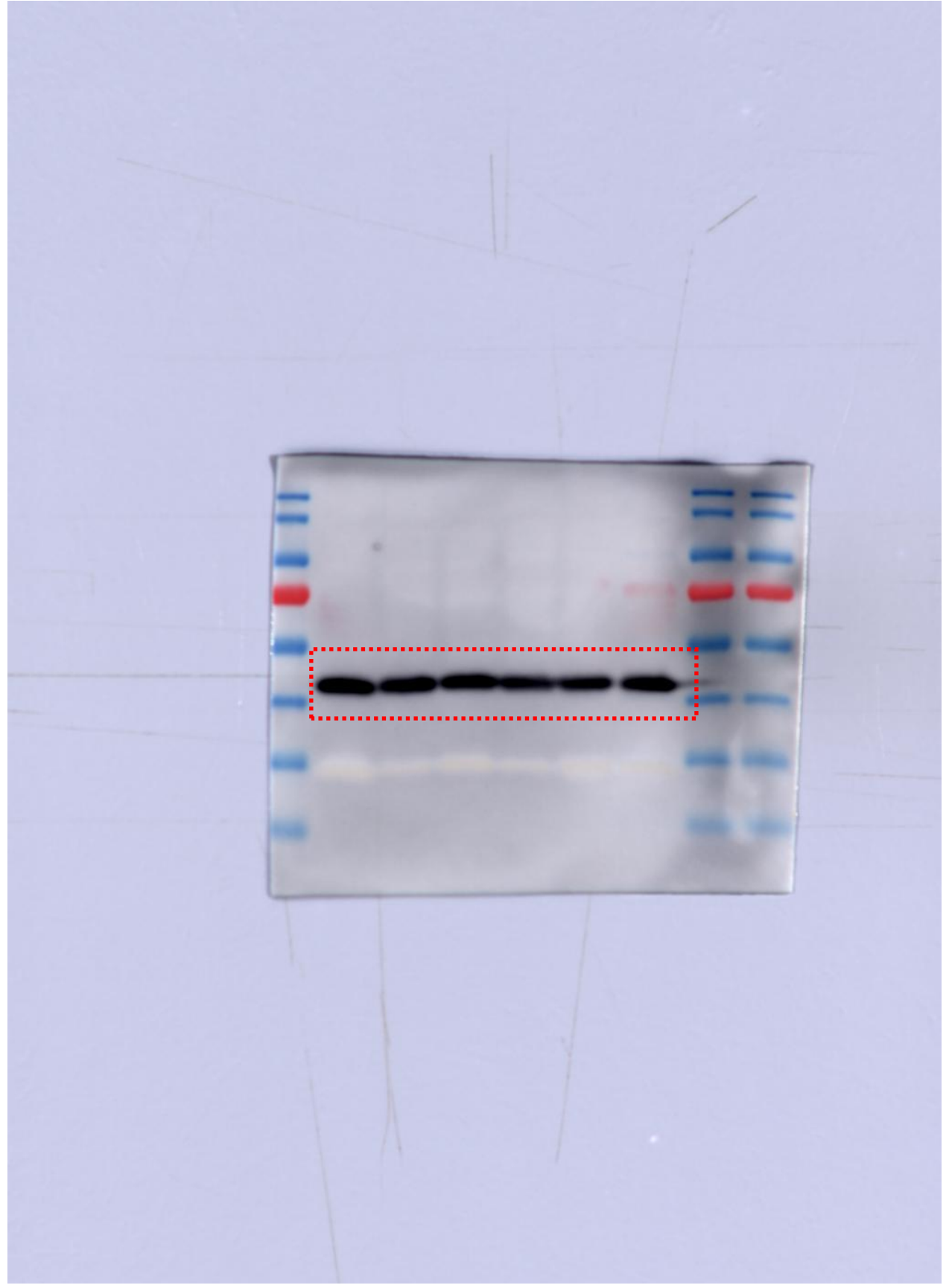

Full unedited blot for Figure 11F

PRDX6

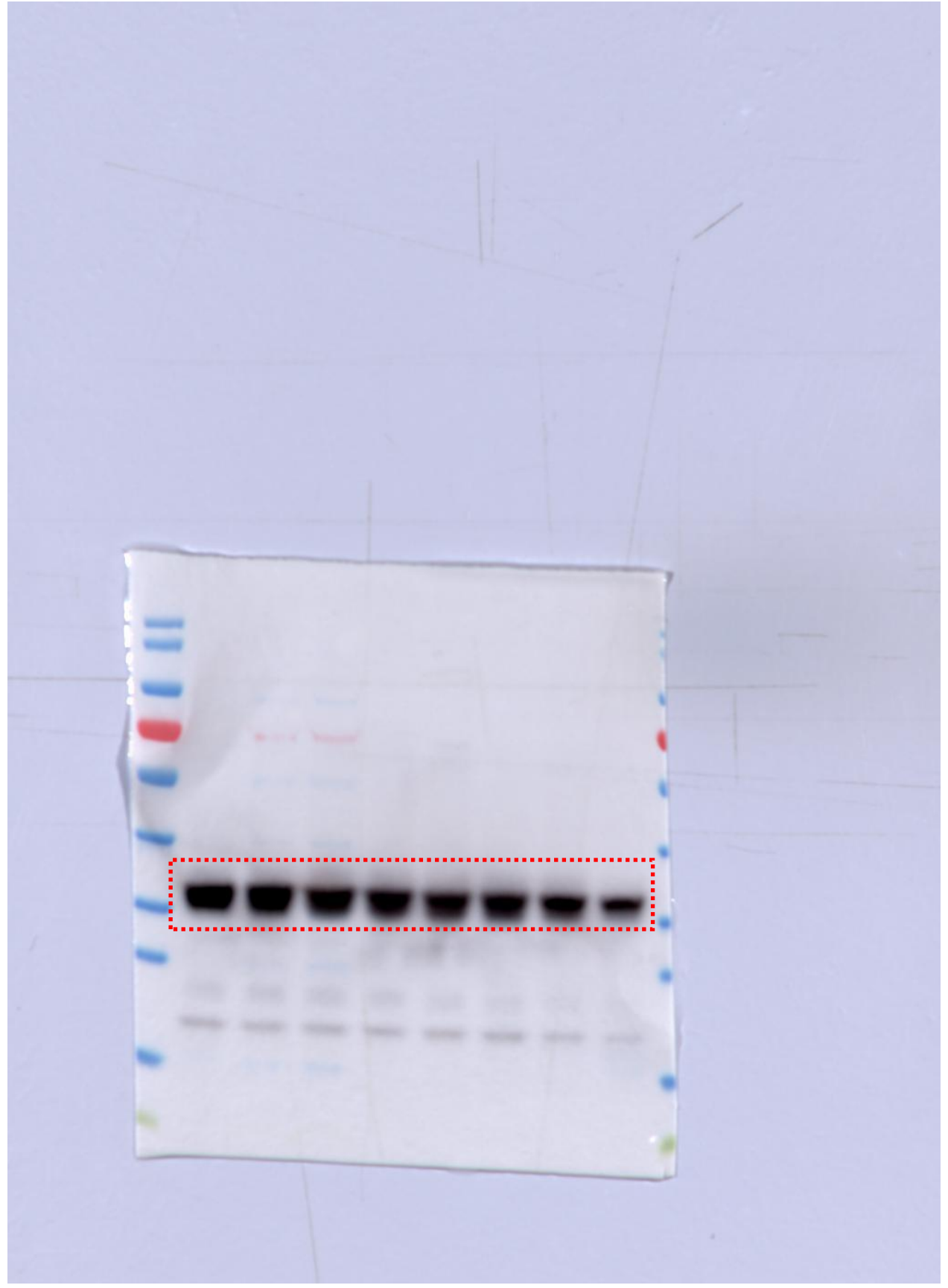

β−actin

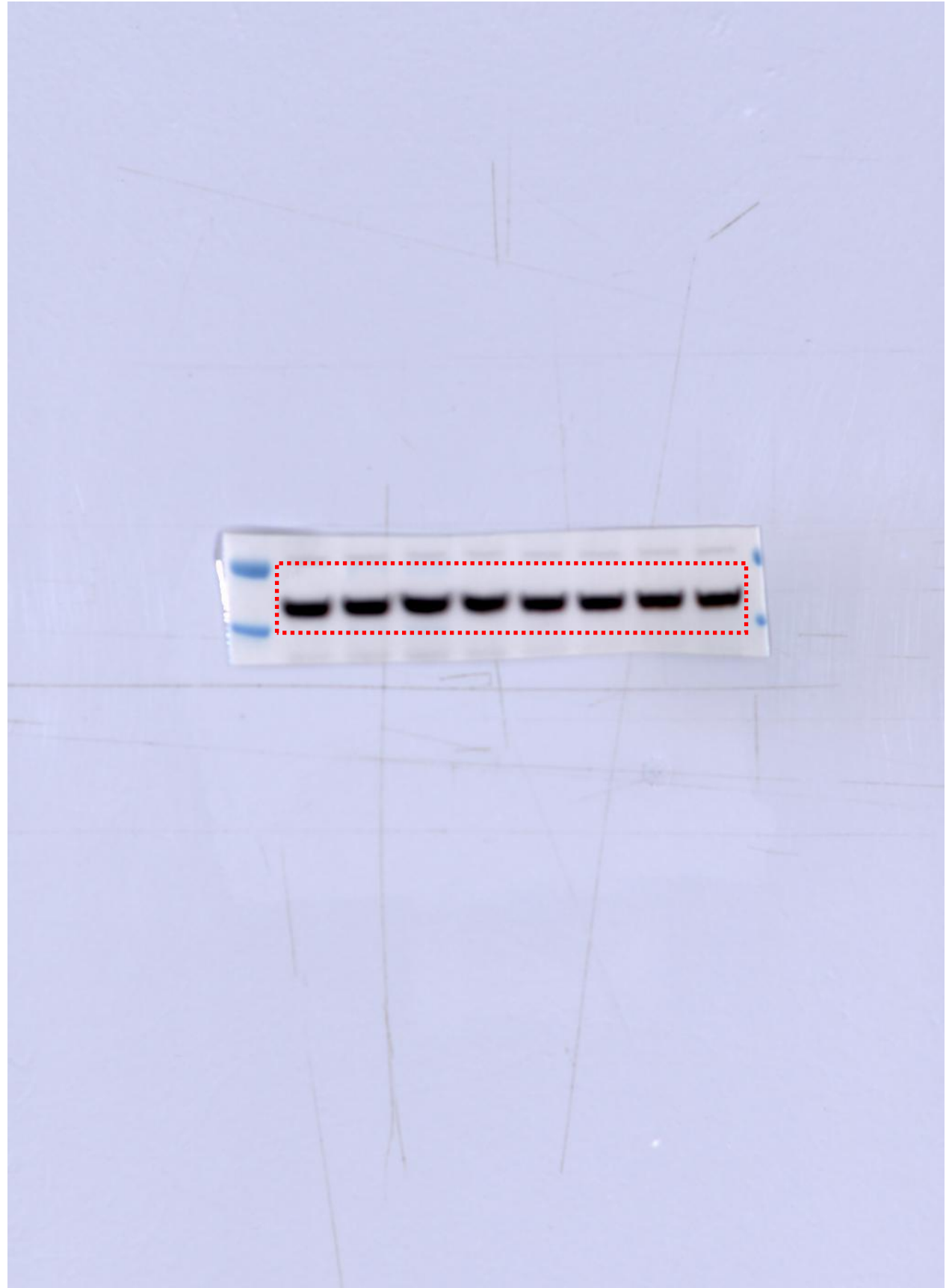

Full unedited blot for Figure 11G

PRDX6

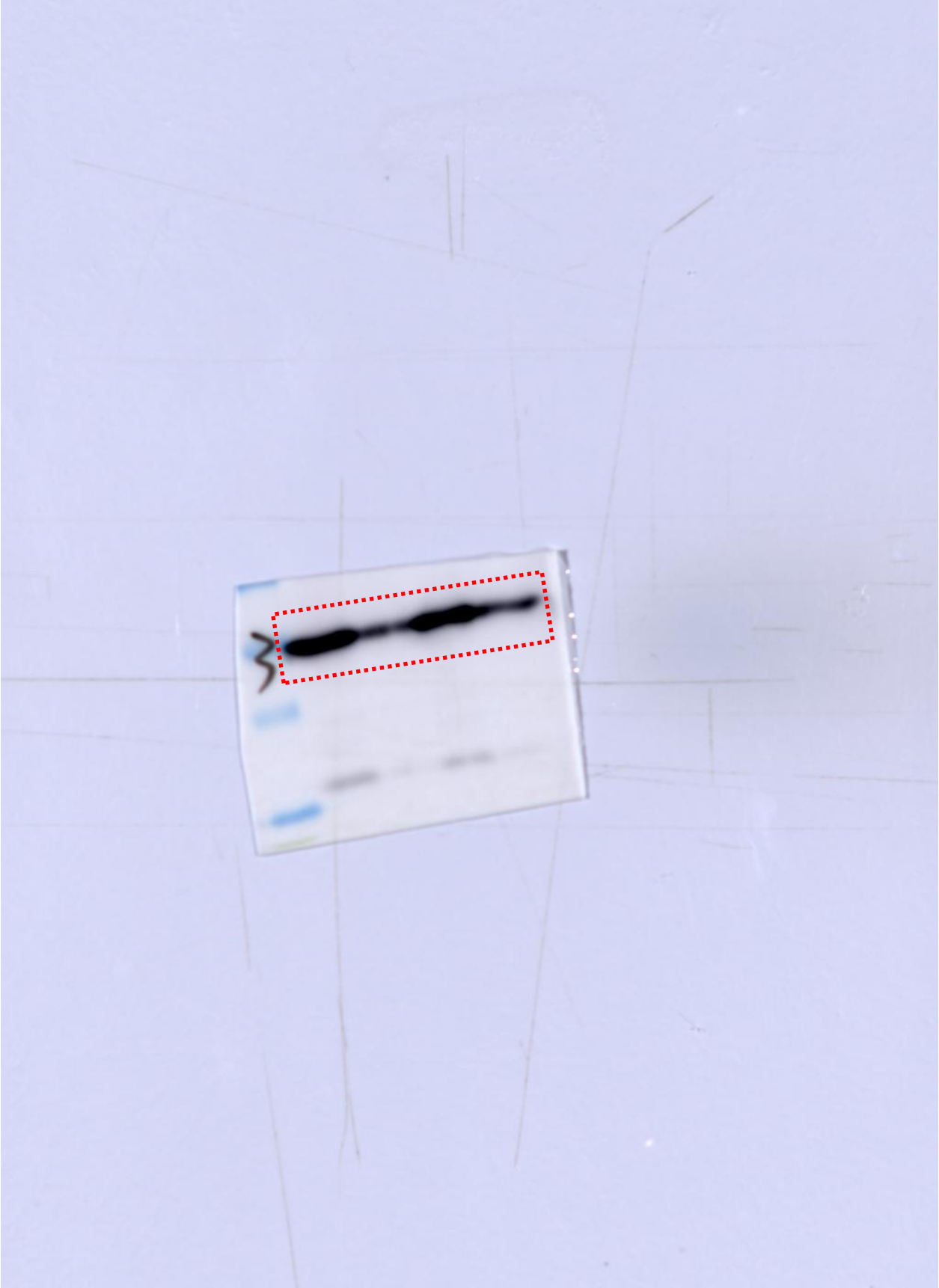

β-actin

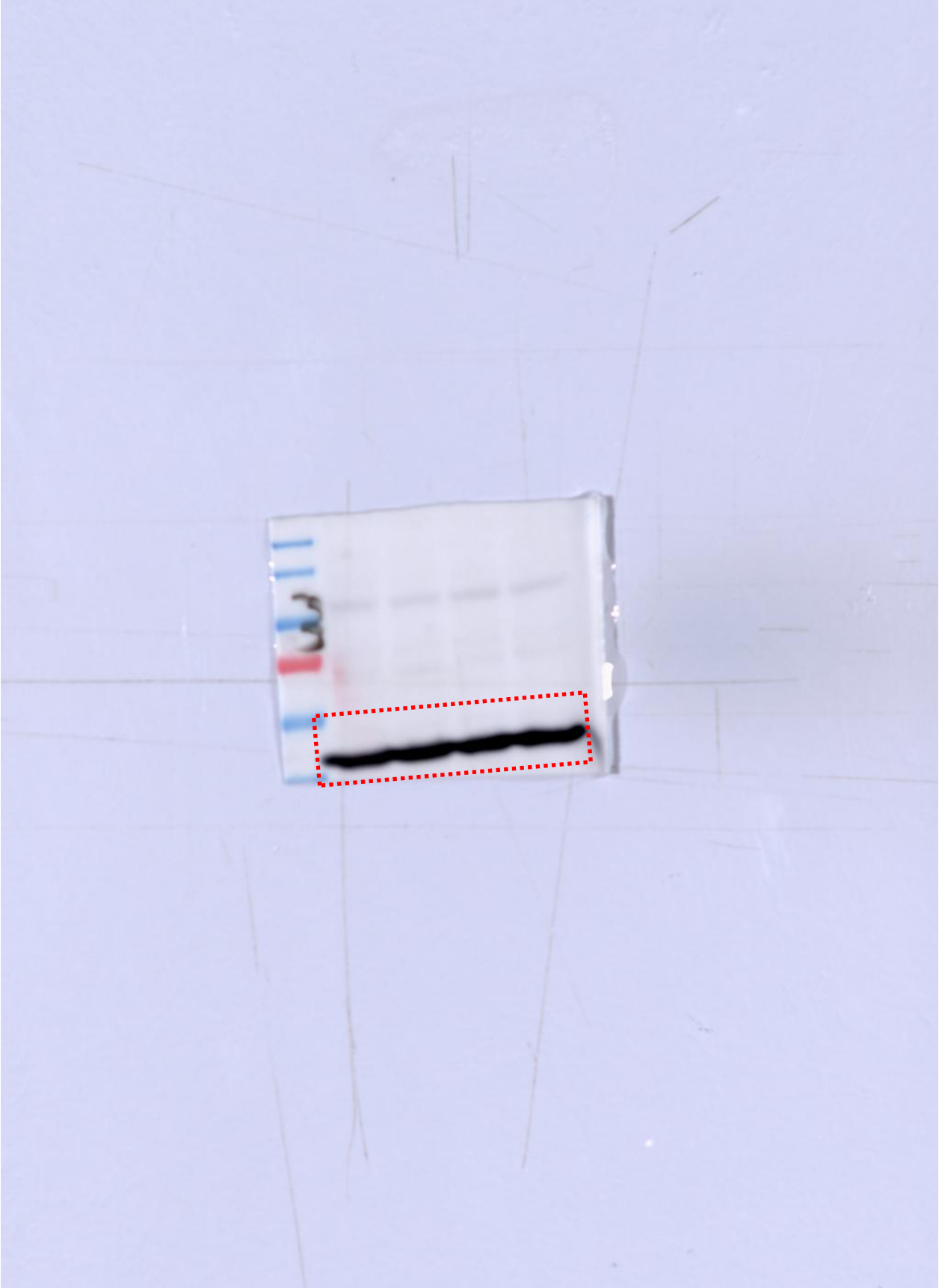

Full unedited blot for Figure 12A

IP: PRDX6 IP: ISG15 Input: PRDX6 Input: ISG15

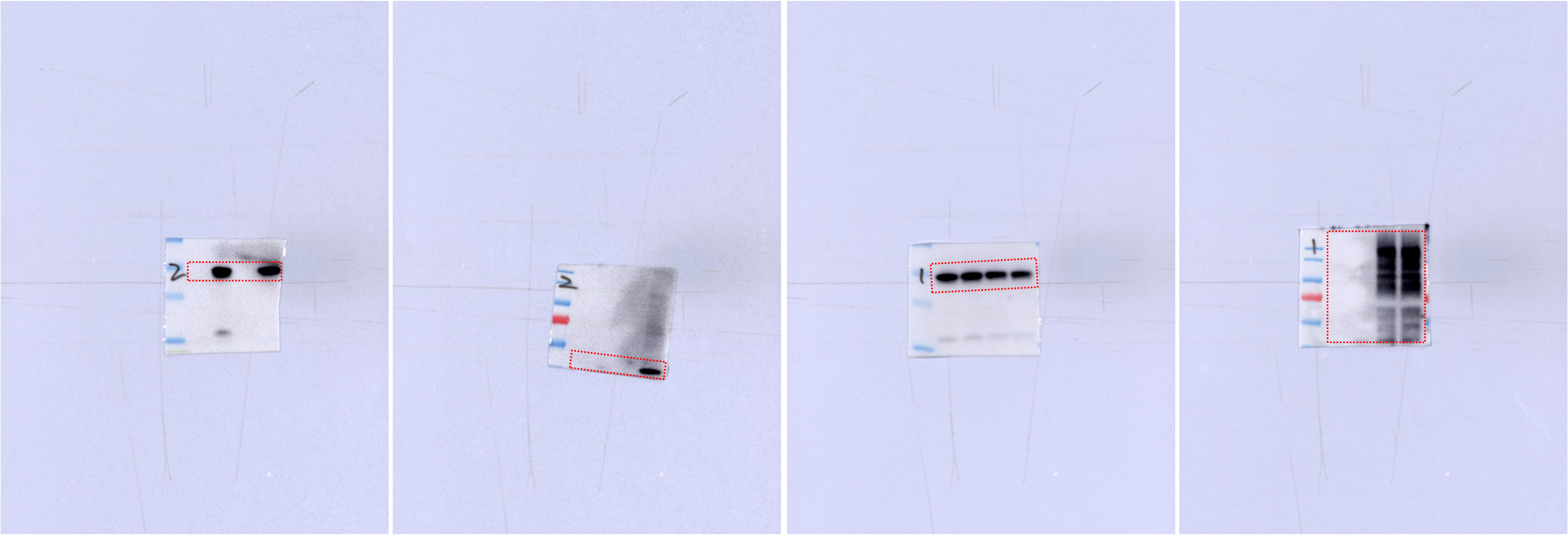

Full unedited blot for Figure 12B

IP: Flag

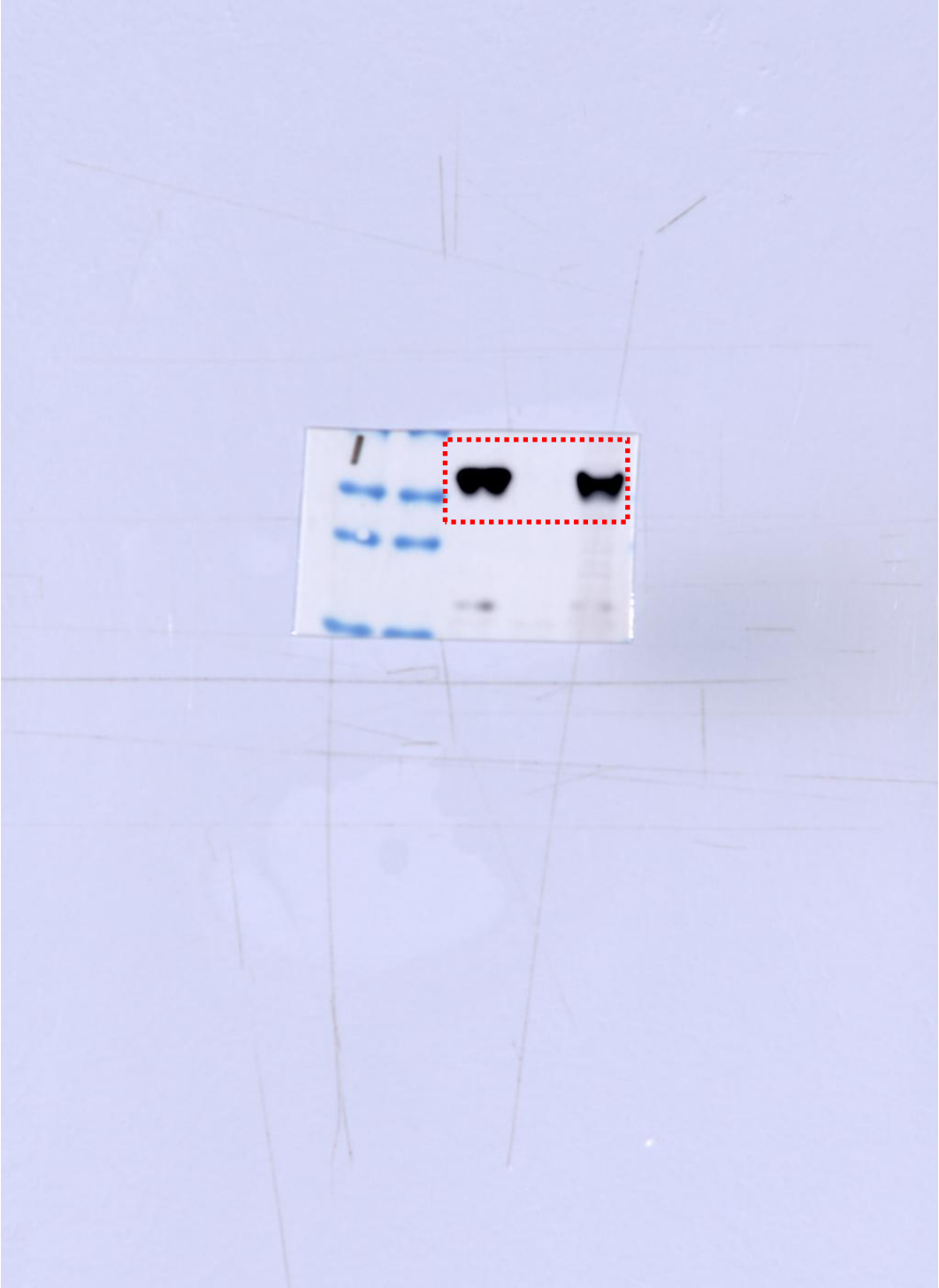

IP: HA

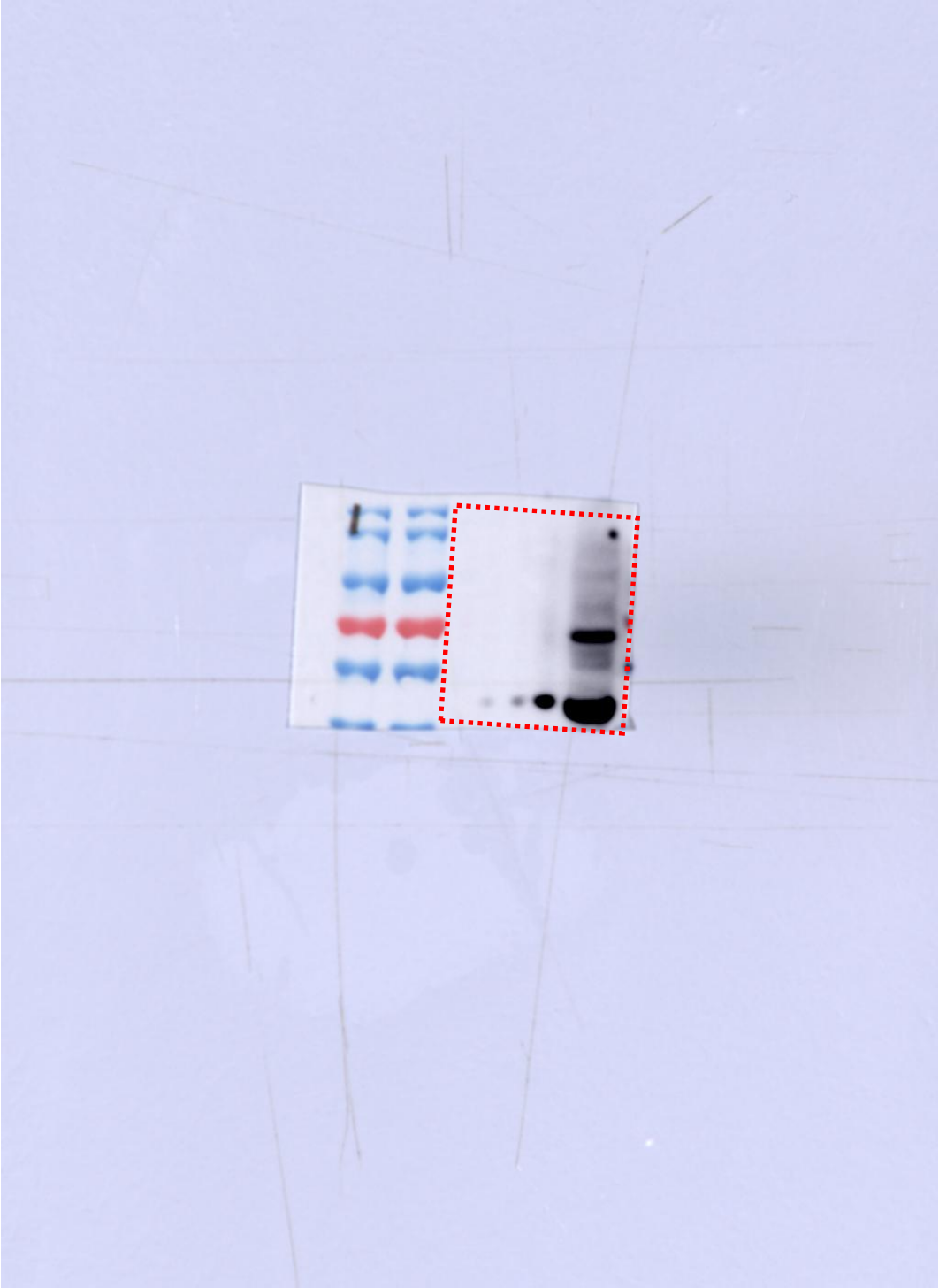

Input: Flag

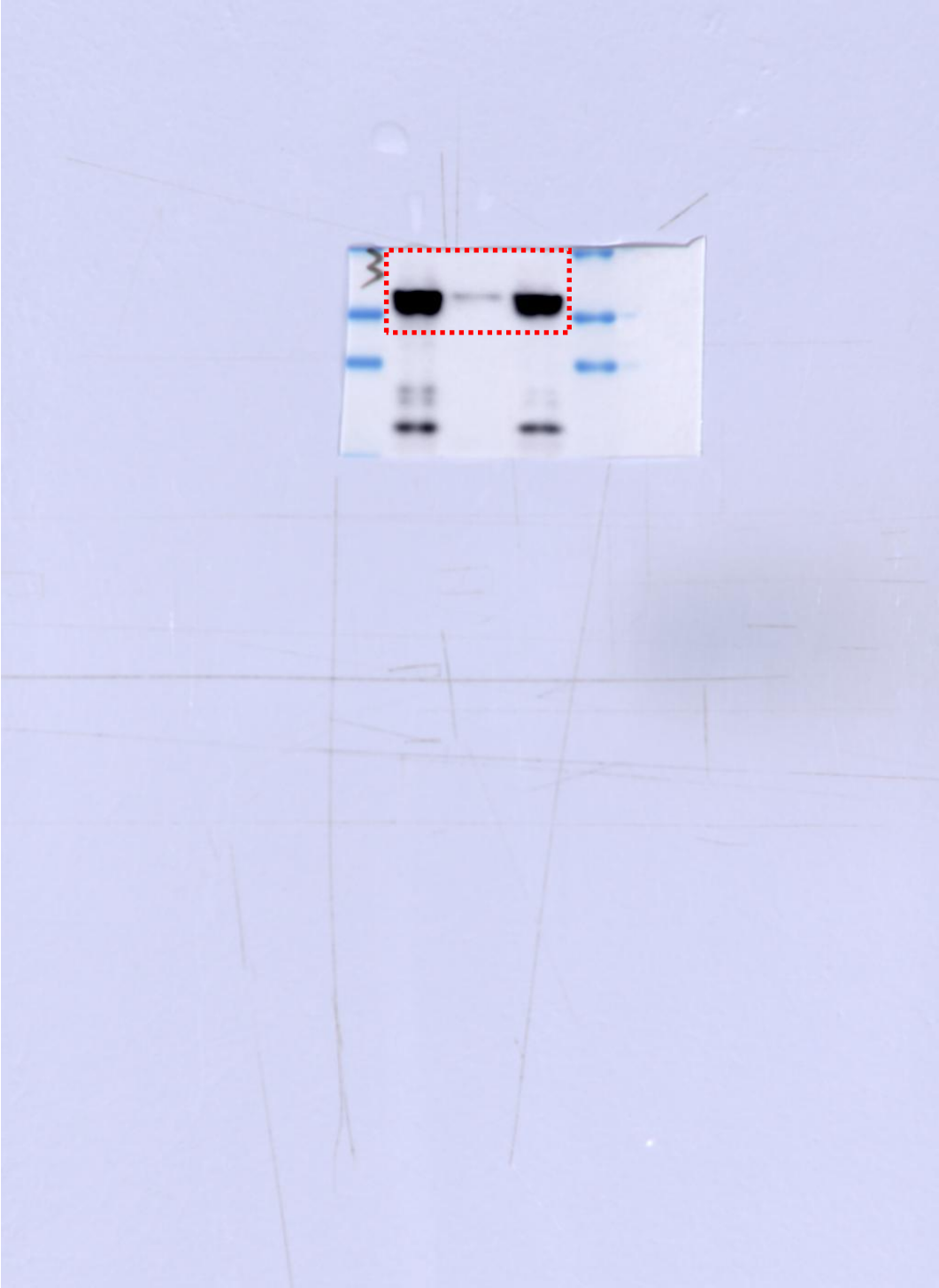

Input: HA

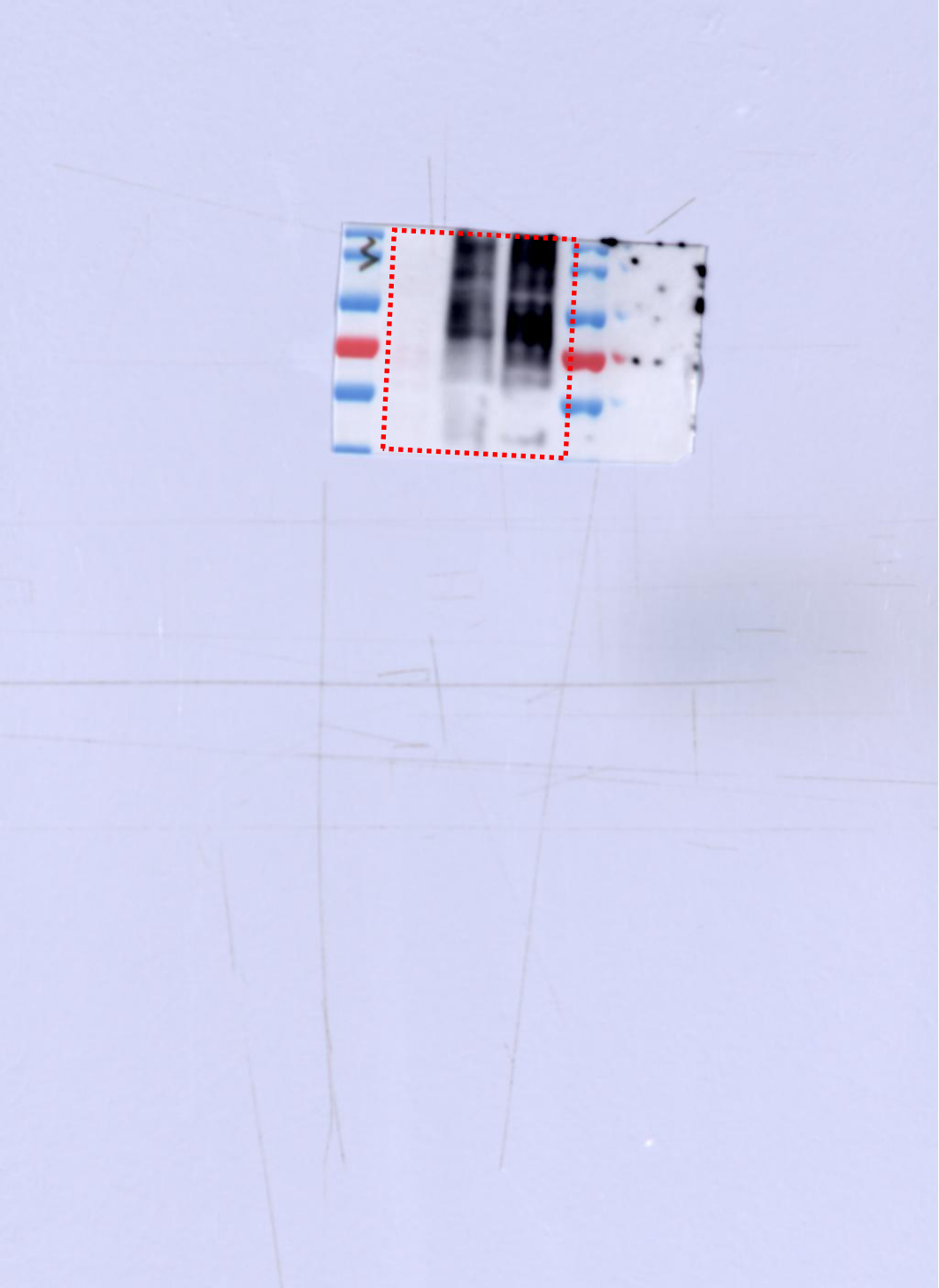

Full unedited blot for Figure 12C

IP: Flag

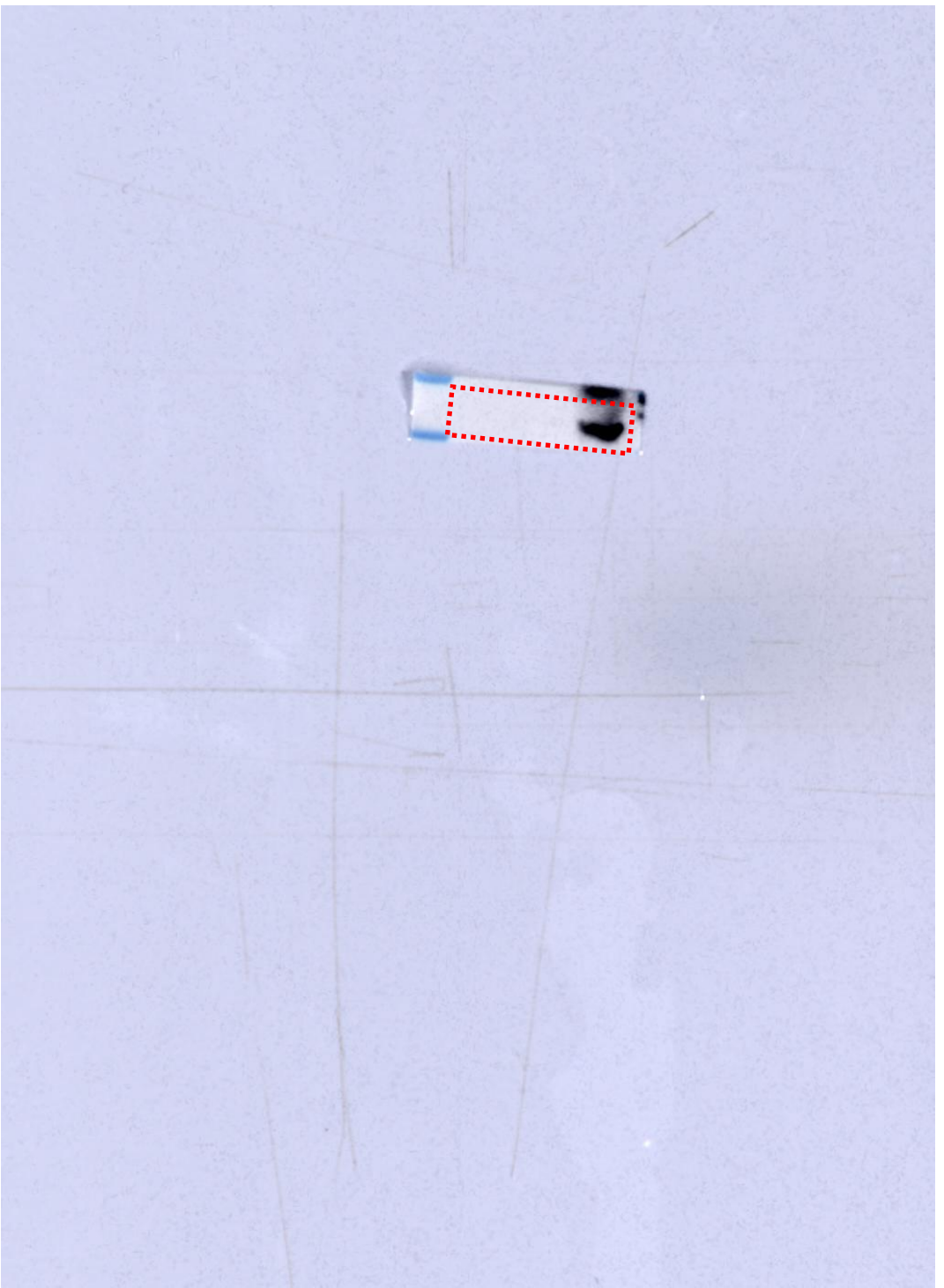

IP: HA

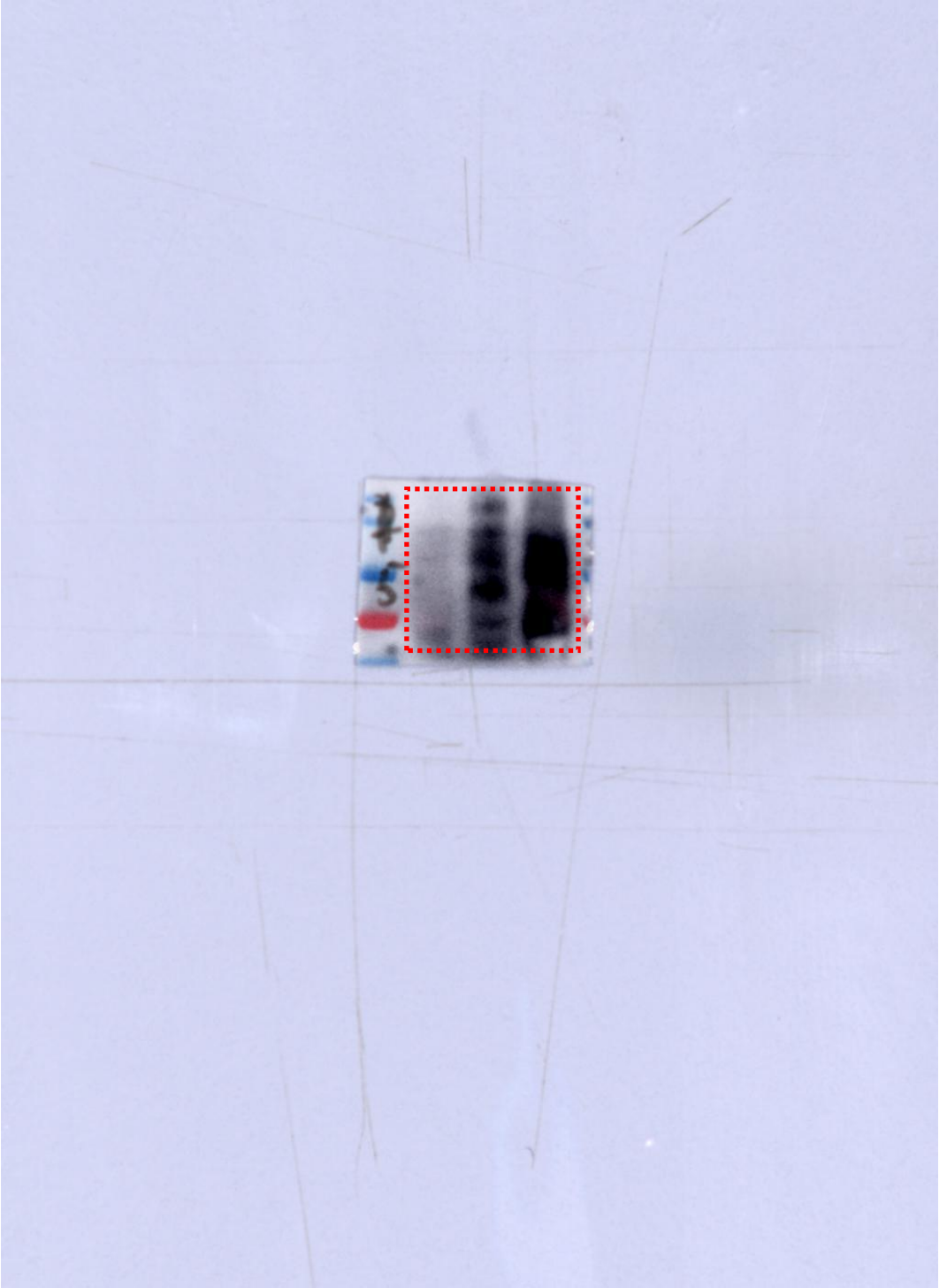

Input: Flag

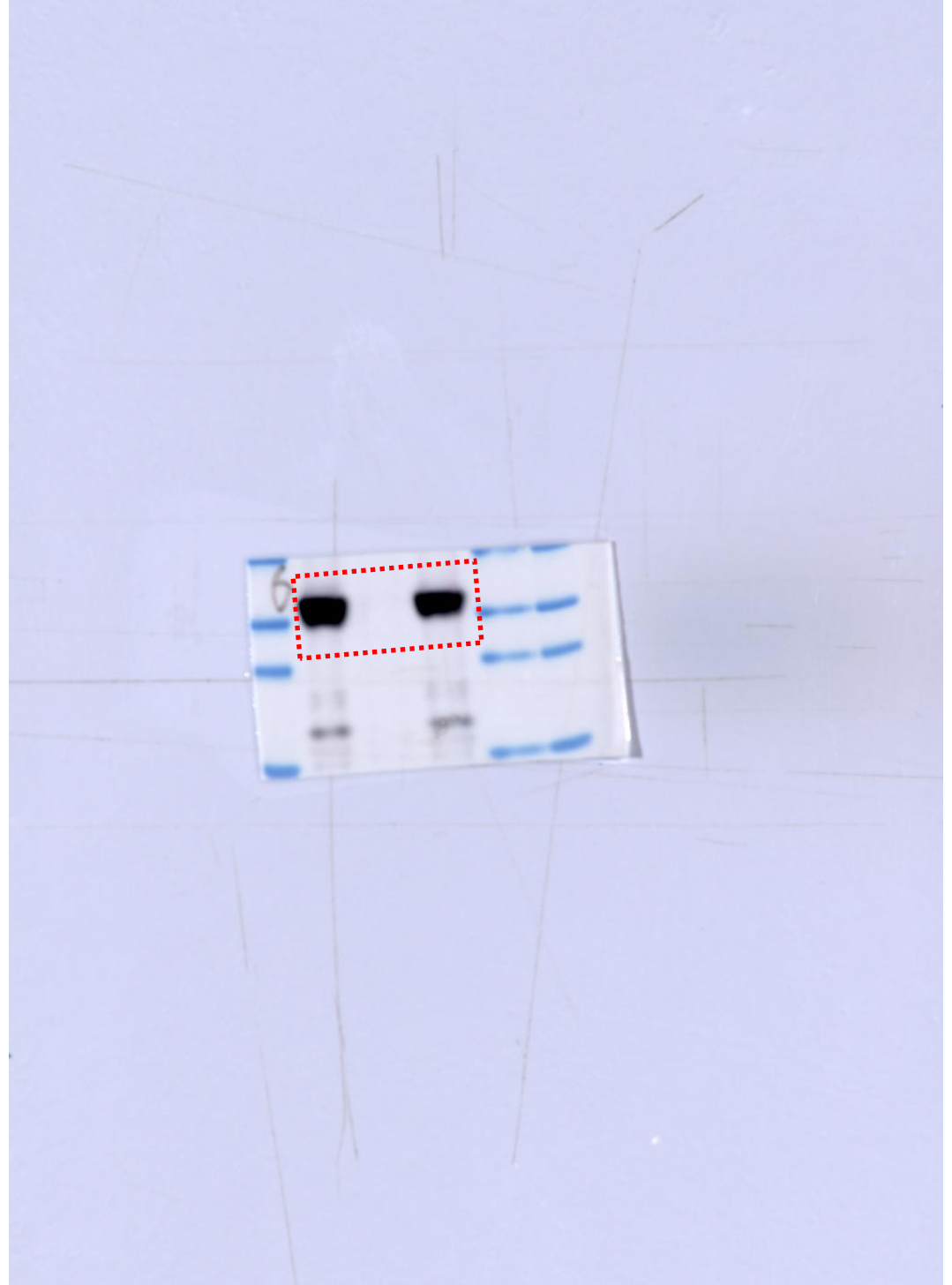

Input: HA

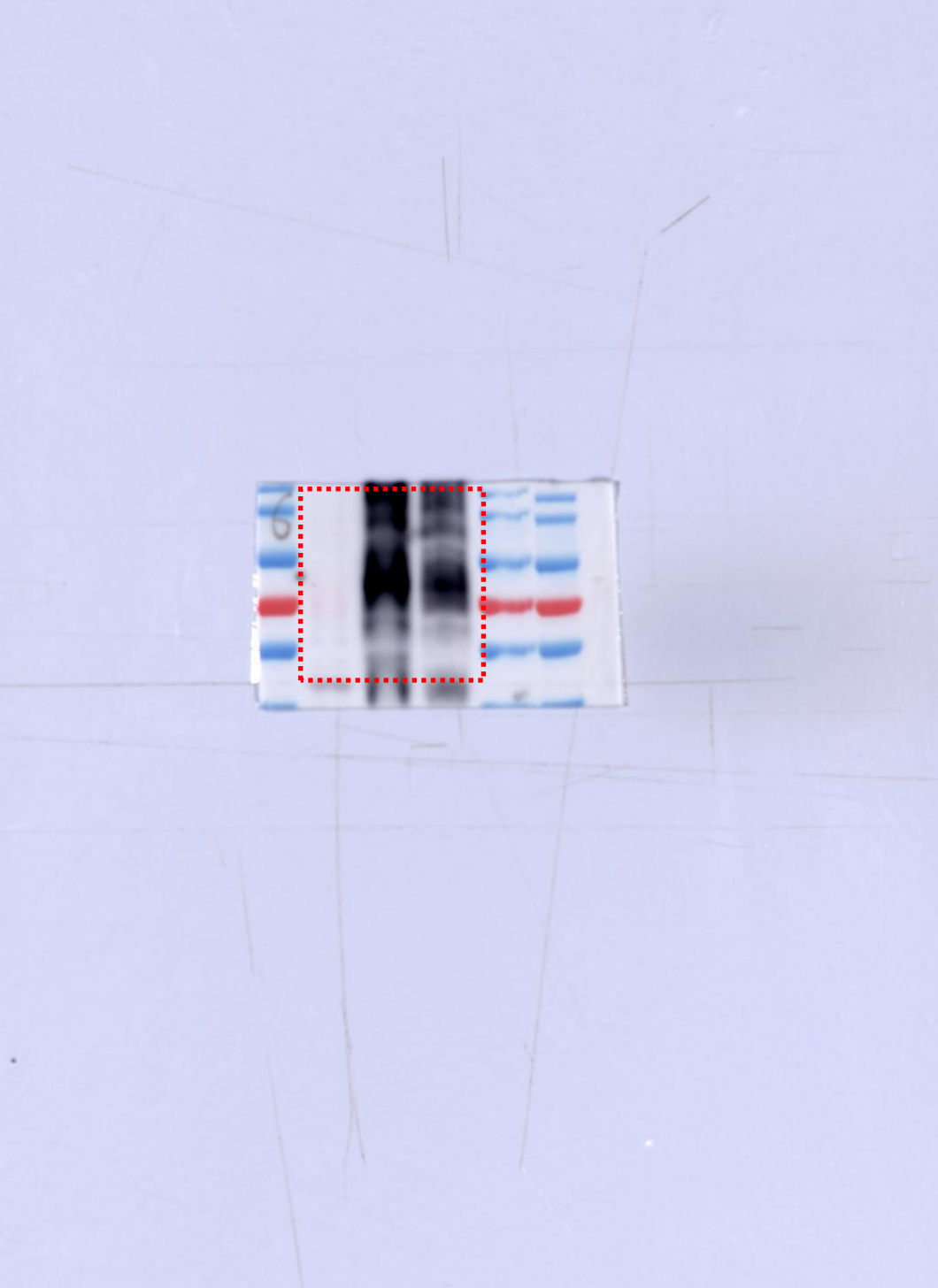

Full unedited blot for Figure 12D

Flag　　HA　　β–actin

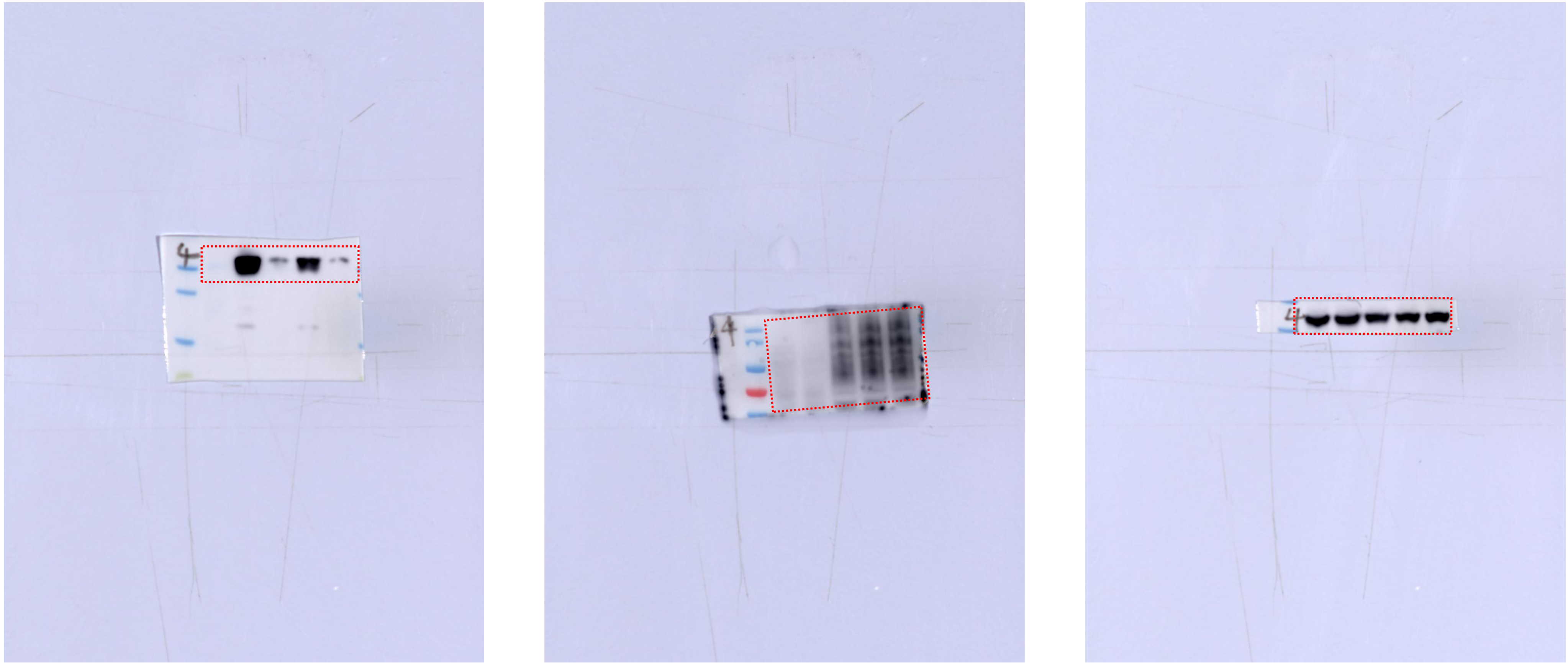

Full unedited blot for Supplemental Figure 7

UBA7

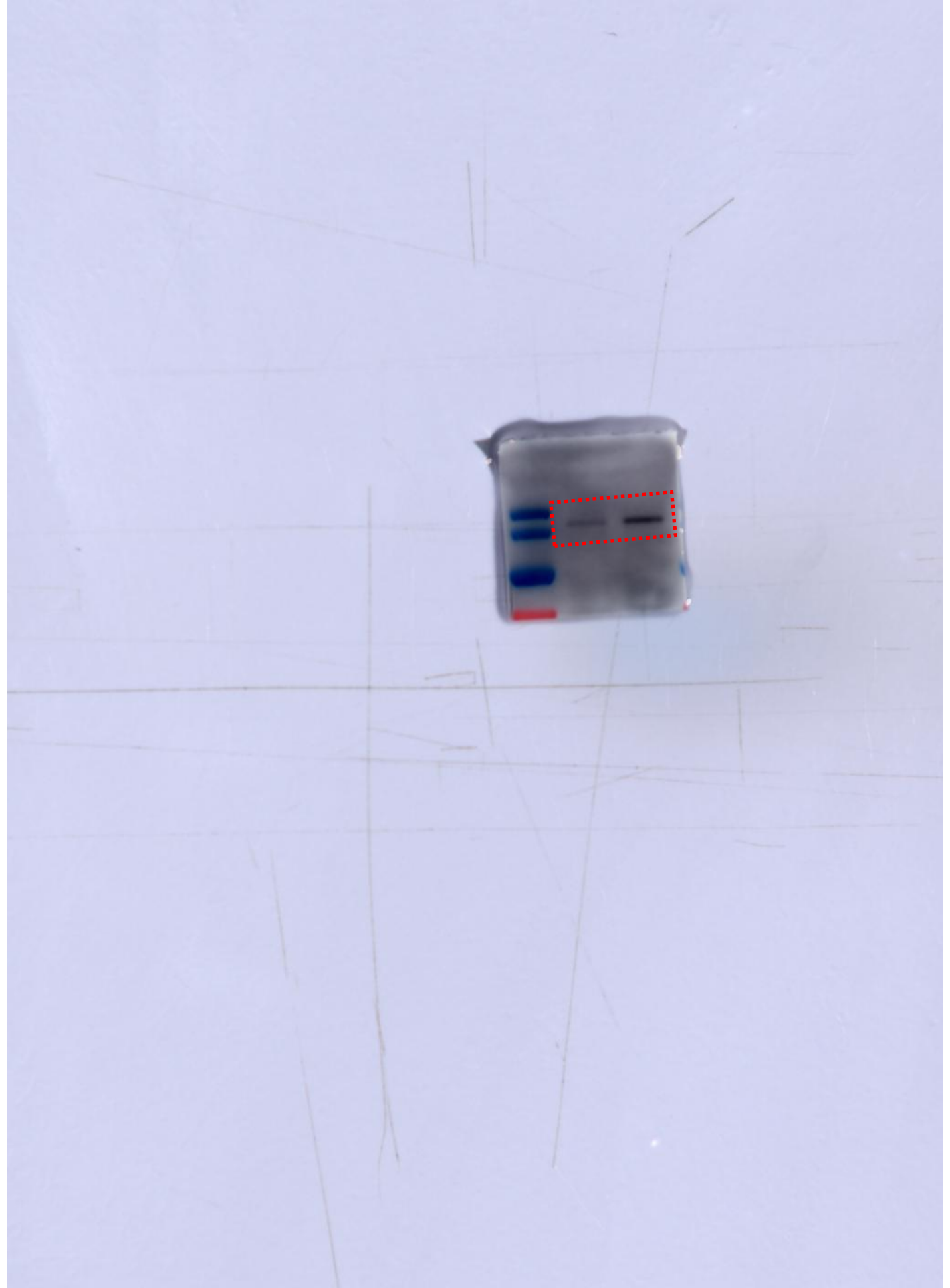

β–actin

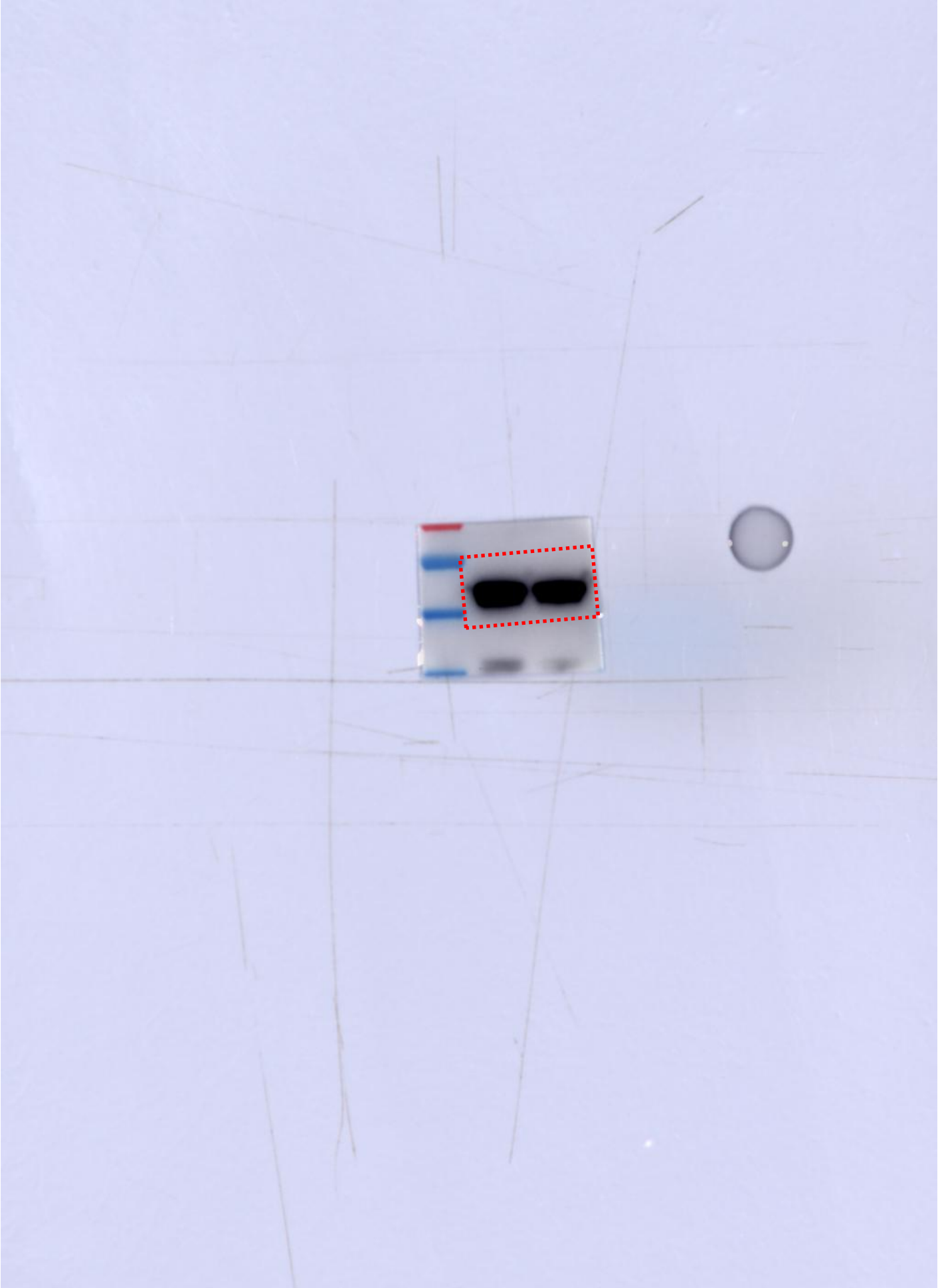

Supplement: Supplementary file 2 [file jci-136-205800-s014.pdf]
